# Integrated Exposomics/Metabolomics for Rapid Exposure and Effect Analyses

**DOI:** 10.1021/jacsau.2c00433

**Published:** 2022-11-07

**Authors:** Mira Flasch, Veronika Fitz, Evelyn Rampler, Chibundu N. Ezekiel, Gunda Koellensperger, Benedikt Warth

**Affiliations:** † Faculty of Chemistry, Department of Food Chemistry and Toxicology, University of Vienna, Währinger Straße 38-40, 1090 Vienna, Austria; ‡ Vienna Doctoral School of Chemistry, University of Vienna, Währinger Straße 42, 1090 Vienna, Austria; § Faculty of Chemistry, Department of Analytical Chemistry, University of Vienna, Währinger Straße 38-40, 1090 Vienna, Austria; ∥ Department of Microbiology, Babcock University, 121103 Ilishan Remo, Ogun State, Nigeria; ⊥ Exposome Austria, Research Infrastructure and National EIRENE Hub, 1090 Vienna, Austria

**Keywords:** exposome, metabolome, human biomonitoring, high-resolution mass spectrometry, exposure, biomarker

## Abstract

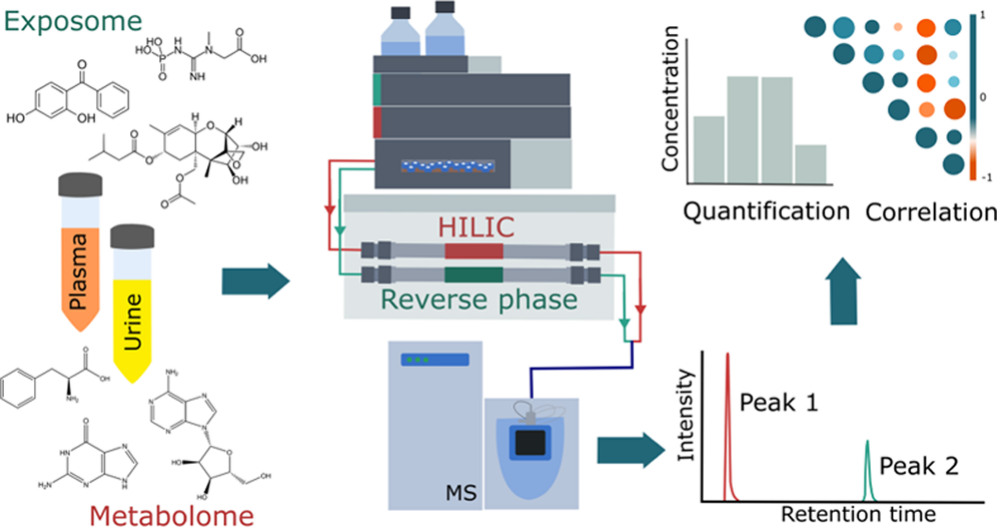

The totality of environmental exposures and lifestyle factors, commonly referred to as the exposome, is poorly understood. Measuring the myriad of chemicals that humans are exposed to is immensely challenging, and identifying disrupted metabolic pathways is even more complex. Here, we present a novel technological approach for the comprehensive, rapid, and integrated analysis of the endogenous human metabolome and the chemical exposome. By combining reverse-phase and hydrophilic interaction liquid chromatography (HILIC) and fast polarity-switching, molecules with highly diverse chemical structures can be analyzed in 15 min with a single analytical run as both column’s effluents are combined before analysis. Standard reference materials and authentic standards were evaluated to critically benchmark performance. Highly sensitive median limits of detection (LODs) with 0.04 μM for >140 quantitatively assessed endogenous metabolites and 0.08 ng/mL for the >100 model xenobiotics and human estrogens in solvent were obtained. In matrix, the median LOD values were higher with 0.7 ng/mL (urine) and 0.5 ng/mL (plasma) for exogenous chemicals. To prove the dual-column approach’s applicability, real-life urine samples from sub-Saharan Africa (high-exposure scenario) and Europe (low-exposure scenario) were assessed in a targeted and nontargeted manner. Our liquid chromatography high-resolution mass spectrometry (LC-HRMS) approach demonstrates the feasibility of quantitatively and simultaneously assessing the endogenous metabolome and the chemical exposome for the high-throughput measurement of environmental drivers of diseases.

## Introduction

Since the ‘exposome’ first emerged as a new paradigm in environmental health describing the entirety of all environmental exposures enclosing lifestyle factors throughout a human’s lifespan,^1^ its scope has expanded further. In recent definitions, endogenous metabolites involved in biological responses (i.e., the endogenous metabolome) that have been triggered by external exposures are typically included.^2,3^

Liquid chromatography high-resolution mass spectrometry (LC-HRMS)-based approaches promise to more comprehensively elucidate the exposome in future exposome-wide association studies (ExWAS). A broad spectrum of small molecules with diverse chemical properties ranging from endogenous metabolites to environmental xenobiotics can be determined with this technique. External stressors, including xenobiotics and environmental changes, are measured simultaneously as phenotypical changes in response to these exposures. Thus, LC-HRMS is an ideal platform for developing more holistic methods to study the exposome.^4^ Its applicability to investigate the impact of environmental toxicants on the endogenous metabolome has previously been showcased.^5,6^ Metabolomics has been applied in large metabolome-wide association studies (MWAS) to investigate the biological mechanisms of diseases, their diagnosis and treatment. Several studies succeeded in deriving biological effects from their data, although data interpretation remains a challenge.^7−11^ The approach also plays an essential role in biomarker discovery and personalized medicine.^12^ However, the study of external stressors and complex environmental exposures is clearly less explored and constitutes the next frontier in the current era of omic-scale exposure measurement and systems toxicology.

Targeted multi-analyte methods are commonly used for human biomonitoring (HBM) of xenobiotics, although most approaches assess only a relatively limited number of different exposure markers or chemical classes.^13−19^ However, recent initiatives aim to expand the coverage of such multi-analyte and multi-class HBM methods to a larger range of xenobiotics, e.g., Jamnik et al.,^20^ who simultaneously assessed more than eighty chemicals many of which have known affinity to the estrogen receptor in relevant biological specimens (blood, urine, and breast milk).

The vast physicochemical diversity of xenobiotics also implies widely varying toxicological effects on humans. The adverse impact of, e.g., mycotoxins, a group of fungal food toxins, range from liver carcinogenicity (aflatoxins), nephrotoxicity (ochratoxin A), and estrogenicity (zearalenone) to the inhibition of protein synthesis and mitochondrial function (trichothecenes).^21^ Xenoestrogens may immensely impact hormone homeostasis and endocrine disruption, especially in critical time windows, since they interfere with the endocrine system, partly even at extremely low concentrations.^22,23^ Estrogenic chemicals occur, for example naturally in plants (phytoestrogens) like genistein or daidzein and synthetic estrogens may be present in pharmaceuticals, insecticides, and plasticizers.^22,24^

Similar to meaningful population-based metabolome research, also exposomics requires large-scale studies for exposome-wide association studies to draw reliable conclusions. The suggested mean sample size for male fertility was estimated to be 2700 men.^25^ Hence, high-throughput methods are urgently needed as time is a limiting factor in large-scale epidemiological investigations. In metabolomics, efforts to increase time efficiency are a current priority.^26,27^ For example, the usefulness of a dual-column approach to gain more information about the metabolome and lipidome within a short run time was described by Schwaiger et al.^28^ Nevertheless, micro- and nano-flow LC-MS have increased in popularity due to increased sensitivity despite longer run times.^29^

The combined and comprehensive measurement of the metabolome and the exposome is challenging as the concentrations of metabolites, drugs, food constituents, and environmental contaminants span approximately ten orders of magnitude and highly diverse classes of chemicals.^3,30^ Here, we present a rapid, high-throughput workflow, combining the analysis of endogenous metabolites and multiple classes of xenobiotics in human urine and plasma. The approach utilizes a dual-column approach with a reversed-phase (RP) and a hydrophilic interaction liquid chromatography (HILIC) column being operated in parallel to cover polar compounds as well as the mostly nonpolar xenobiotics. The exposome coverage was evaluated based on more than 200 highly diverse analytes comprising endogenous metabolites and xenobiotics to prove the power of the new method. The applicability to real-life samples was demonstrated by the analysis of urine samples of test sub-populations from Nigeria and Austria.

## Materials and Methods

### Chemicals

A multi-analyte stock solution contained endogenous human metabolites (145 analytes) at 50 μM (median of 9300 ng/mL) and xenobiotics, including estrogenic compounds and human estrogens (106 analytes) at a concentration between 5 and 5000 ng/mL (median 100 ng/mL) was prepared in ACN/water (50:50; v/v) (Figure S1). In the context of this paper, the estrogens are evaluated together with the xenobiotic substances and mentioned accordingly. In addition, 15 different isotopically labeled standards of xenobiotics and a separate ^13^C- labeled yeast extract (ISOtopic solutions, Vienna) were used as internal standards. A complete list of all analytes is available in the Supporting Information (Table S1). A 24 h pooled urine sample obtained from a healthy female volunteer collected in one day after three days of a low-xenoestrogen/polyphenol diet was chosen as a model matrix in this study since urine is frequently used for assessing chemical exposure. Moreover, as a second model matrix, pooled human Li-heparin plasma was acquired from Innovative Research (Novi). Arylsulfatase/β-glucuronidase from *Helix pomatia* was purchased from Sigma-Aldrich (Vienna, Austria). All materials were stored at −80 °C prior to extraction. The concentrations of all analytes (xenoestrogens, mycotoxins, endogenous estrogens, and other endogenous metabolites) in the calibration standards (8 levels) are listed in the Supporting Information (Table S2).

### Samples

For the optimization of the eluent/column combination, a solvent standard and a matrix-matched standard at a medium concentration range (Level 6; Table S2) were used. SRM1950 (Metabolites in Frozen Human Plasma) and SRM3672 (Organic Contaminants in Smoker’s Urine) were purchased from the National Institute of Standards & Technology (NIST, Gaithersburg). In addition, 24 h urine samples from a food intervention study performed in 2021 with four individuals (two females and two males) collected at three different time points (three different days) were tested. For details, kindly refer to Oesterle et al. (2022).^55^ Furthermore, urine samples from Nigerian women sampled in 2016 were investigated. This longitudinal sample set was already analyzed before on biomarkers of mycotoxin exposure.^31^ Therefore, not all samples from the original study were available due to limited sample volumes. The sample set included 77 spot urine specimens from four time points (morning and evening over two days) of 23 mothers. All samples were stored at −80 °C until analysis. The Nigerian study was approved by the Ethics committee of Babcock University in Nigeria (BUHREC294/16) and the Austrian samples were collected following approval by the University of Vienna ethics committee under authorization number #00650.

### Sample Preparation

During the whole sample preparation procedure, the samples were kept on ice. At first, 200 μL of urine or plasma was mixed with 20 μL of the internal standard mix (Table S3) and 20 μL of the ^13^C-labeled yeast metabolite extract. For the experiment to determine the best solvent/column combination no internal standard was used. Therefore, only 40 μL of H_2_O was used instead of the internal standard mix. The samples were then vortexed. Afterward, the samples were mixed with 760 μL of the extraction solvent (ACN/ MeOH (1:1, v/v)). After thoroughly vortexing and sonication in an ice bath (10 min), the samples were placed at −20 °C for 2 h and centrifuged at 18,000*g* and 4 °C (10 min), and 960 μL of the supernatant was transferred to a new tube. Then, the samples were evaporated in a vacuum concentrator (Labconco). The residues were reconstituted in 192 μL of solvent (ACN/ water, 50:50, v/v), vortexed, and centrifuged at 4 °C for 10 min. Finally, the supernatants were transferred to HPLC vials and stored at −80 °C until analysis. Moreover, matrix-matched calibration standards for urine and plasma were prepared by reconstituting matrix extraction according to the sample preparation protocol with respective solvent standard solutions. SRM3672 was additionally treated with β-glucuronidase/arylsulfatase enzymes from *Helix pomatia* for 12 h at 37 °C prior to extraction as the NIST-certified reference values are given for deconjugated samples. This additional step was not performed for the other samples because the heat treatment would have disturbed the measurements of endogenous metabolites. A nondeconjugated sample was prepared for the SRM material for comparison as well.

### Quality Control Measures

As quality control samples, SRM1950 in one to ten dilutions and a solvent QC comprising all target analytes at 1 μM (median of 186 ng/mL, metabolites) and approximately 100 ng/mL (xenobiotics) were analyzed throughout the sequence. Moreover, 10 μL of each urine sample was pooled together for the Austrian and Nigerian samples separately. This pooled urine sample from the Austrian study was measured every nine injections while measuring these samples. The Nigerian pooled urine sample was injected repeatedly with a maximum of nine individual samples in between to check the instrument performance. Solvent blanks (pure reconstitution solvent) and system blanks (200 μL of water extracted according to the sample preparation protocol) were measured to correct for contaminations in the system and during sample preparation.

### LC-HRMS(/MS) Analysis

A Vanquish Duo UHPLC system with two independent pumping systems and two different columns was used to optimize chromatographic separation for our highly diverse set of endogenous and exogenous analytes. Different column/eluent systems were examined. The RP gradient was derived from Jamnik et al.^20^ and the HILIC gradient was based on Schwaiger et al.,^28^ Narduzzi et al.^32^ and Galvez et al.^33^ The NH_4_F concentration in the aqueous RP eluent was increased to 0.6 mM NH_4_F to ensure at least 0.3 mM NH_4_F after dilution with the effluent from the other column before introduction to the MS. If NH_4_F was used in both eluents, the modifier’s concentration was increased to in total 2 mM NH_4_F as Narduzzi et al.^32^ used this concentration for the HILIC gradient. As a reverse-phase column (RP), the Acquity HSS T3 (Waters, 1.8 μm, 100 mm × 2.1 mm) was used. For HILIC chromatography, two different hydrophilic interaction liquid chromatography columns (HILIC) were used, namely, a SeQuant ZIC-pHILIC (Merck, 5 μm, polymeric, 150 mm × 2.1 mm) and an Acquity BEH Amide (Waters, 1.8 μm, 100 mm × 2.1 mm). Eluent B was in all cases 100% ACN. The aqueous eluent (solvent A) was changed as stated in Table 1. The selection of the NH_4_F concentration in the aqueous HILIC eluent was based on Narduzzi et al.^32^ at either 1 or 2 mM. The injection volume was 5 μL for all columns and experiments.

**Table 1:** Optimization Design of the Tested Column/Eluent Systems for the Dual-Column Approach

	combination 1	combination 2	combination 3	combination 4
	**RP**			
column	Acquity HSS T3			
aqueous eluent	0.6 mM NH_4_F in H_2_O	0.3 mM NH_4_F in H_2_O	1 mM NH_4_F in H_2_O	0.6 mM NH_4_F in H_2_O
organic eluent	ACN			
	**HILIC**			
column	SeQuant ZIC-pHILIC			Acquity BEH amide
aqueous eluent	10 mM NH_4_HCO_3_ (pH 9.2) in H_2_O/ACN (9:1, v/v)	2 mM NH_4_F in H_2_O	1 mM NH_4_F in H_2_O	50 mM CH_3_COONH_4_ (pH 6) in H_2_O
organic eluent	ACN			

**Figure 1:**
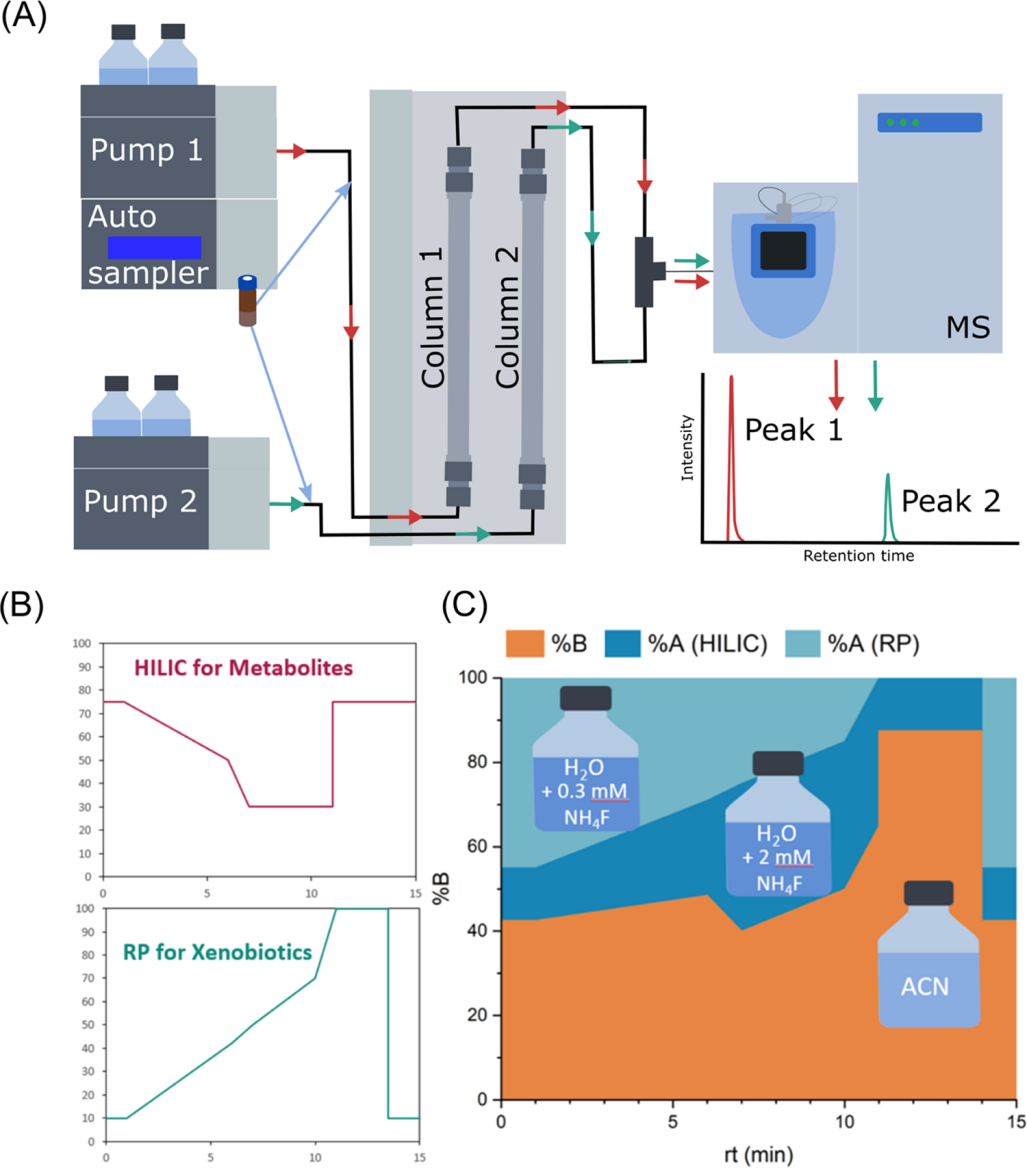
Dual-column setup (A) LC-HRMS system comprising two separate LC-pumps and columns combined by a T-piece before entering the mass spectrometer. (B) Gradients of both individual columns. (C) Eluent composition entering the mass spectrometer after the effluents were mixed.

For the RP measurements, the gradient was as follows: 0–1 min, constant flow at 10% B; 1–10 min, increase to 70% B; 10–11 min, increase to 100% B, and 13.5–15 min, equilibration at 10% B. A hydrophilic interaction liquid chromatography (HILIC) column, SeQuant ZIC-pHILIC (5 μm, polymeric, 150 mm × 2.1 mm), was operated with the following gradient: 0–1 min, constant flow at 75% B; 1–6 min, linear decrease to 50%; 6–7 min, decrease to 30% B; 7–11 min, constant flow at 30%; 11–15 min, equilibration at 75% B. Both columns were at 40 °C and a flow rate of 0.3 mL/min was set. The Acquity BEH Amide was operated with a slightly different gradient as follows: 0–2 min, constant at 80% B; 2–8 min decrease to 40% B; 8–10 min, constant at 40% B and 10–15 min, equilibration at 80% B. Both capillaries were connected with a T-piece to mix the effluents before introduction into the ESI source of the mass spectrometer (Figure 1A). As needle wash, 75% ACN was used for the HILIC measurement, while H_2_O:ACN: MeOH (2:1:1,v/v/v) was applied for the RP run.

Measurements were conducted in fast polarity-switching full scan mode on a Q Exactive HF quadrupole-Orbitrap mass spectrometer. The settings of the ESI interface were as follows: sheath gas, 48 au; auxiliary gas, 11 au; sweep gas flow, 2 au; capillary voltage, 3.5 kV (positive), 2.8 kV (negative); capillary temperature, 260 °C; auxiliary gas heater, 410 °C. The scan range was from 65 to 900 *m*/*z*. For full scan-only measurements, the resolution was set to 60,000 with an AGC target (automatic gain control) of 1 × 10^6^ and a maximum injection time of 200 ms. The instrument was calibrated before analysis.

Following the optimization of chromatographic conditions, the most suitable RP/HILIC combination was selected. Consequently, the parameters mentioned above for combination two (Figure 1B) were applied for the sample measurement. A mixture of both effluents entered the mass spectrometer via the ESI source (Figure 1C). For analyses with data-dependent MS2, a resolution of 60,000 with an AGC target of 1 × 10^6^ and a maximum injection time of 100 ms were chosen for the full scan. The settings for the MS2 collection were as follows: resolution, 30,000; AGC target, 1 × 10^5^; maximum injection time, 50 ms, loop count, 10; isolation window, 1.0 *m*/*z*; normalized collision energy, 30 eV; minimum AGC, 8 × 10^3^; dynamic exclusion, 4 s. Iterative exclusion lists were generated with IE-Omics.^34^ The urine samples from Nigeria and Austria were analyzed in a randomized order.

### Data Analysis

Skyline (version 20.2.0.286,^35^) was used for targeted analysis and quantification. The peak retained on the HILIC column was used for the quantification of endogenous metabolites (excluding estrogens) while for xenobiotics the RP peak was chosen in this work. The respective peak was selected based on the retention time derived from injecting only on one column. The other peak (usually the first, less retained one) was not used for our evaluation. An internal standard correction was performed. If no internal standard was included for the specific analyte, the internal standard with the closest retention time was selected as a surrogate standard for normalization (Table S7). The linear calibration curves (Table S7) were 1/*x* weighted. Matrix-matched calibration for urine and plasma was performed and the corresponding calibration curves were used for the quantification of xenobiotics and human estrogens. However, endogenous metabolites were, as expected, frequently highly abundant in the matrices. Therefore a solvent calibration was applied for endogenous metabolites (excluding estrogens). The creatinine levels were quantitated by one-point calibration based on the creatinine level in SRM3672 (734 mg/L). The limits of detection (LODs) were determined based on the EURACHEM guideline^36^ as three times the standard deviation of multiple injections of a less-concentrated standard (*n* = 6) divided by the square root of the number of replicates. For the limit of quantification (LOQ), the tenfold standard deviation was used. The recoveries were calculated by dividing the spiked concentration by the measured concentration multiplied by 100. The expected concentration level in the spiked samples used for recovery calculation corresponded to standard level 6.

Superman correlation was calculated for compounds positive in at least 20% of all Nigerian samples with R and plotted using the corrplot package (version 0.90).^37^ MetaboAnalyst 5.0 Pang et al. (2021) was used for pathway analysis.^56^ A hypergeometric test was selected as an enrichment method and for topology analysis relative-betweenness centrality. The pathway library was *Homo sapiens* (KEGG).

The pooled Nigerian urine sample, including MS2 data measured in negative and positive ionization mode with iterative exclusion lists (*n* = 4), was used to screen for potential additional xenobiotics not covered by the targeted evaluation using authentic reference standards. Suspect screening was performed in R, applying the patRoon package.^38^ Solvent process blanks (only in the corresponding polarity) were defined as blank measurements. The raw data files were converted to mzML files and centroided with ProteoWizard.^39^ For peak picking and grouping, the “openms” algorithm was set with the following parameters: noise threshold: 4E3, chromFWHM: 3, minFWHM: 1, maxFWHM: 30, chromSNR: 5, and mzPPM: 3. Only features with a minimum absolute feature intensity of 3E5, a minimum feature intensity above blank of 10, present in at least 60% of replicates, were kept and blank analyses were removed after this step. A suspect list from the ENTACT trial^40^ based on the EPA’s ToxCast library, including >4000 substances, was adopted for this experiment. Analytes, which had already been included in the targeted list were removed to avoid redundancy. For suspect screening, an *m*/*z* window of 0.002 was set. MS peak list data were extracted with the mzr algorithm (precursor *m*/*z* window: 0.5) and filtered (relative intensity threshold: 0.02, top 10 MS/MS peaks). Then molecular formulas were generated considering [M + H]^+^ and [M – H]^−^ adducts, and the elements C, H, N, O, P, S, Cl, Br using genform. Chemical compounds were annotated with metfrag and the comptox database. The suspect screening results were refined using annotateSuspects and the generated peak lists, formula, and compound data. An identification level was assigned depending on the rank and scores (isoScore, individualMoNAScore) of formula/compound candidates based on Schymanski et al.^41^ The default settings in the annotateSuspects algorithm were applied. As no retention time data were available, level one identifications were not possible. The other identification levels were: level 2a (good MS/MS library match, top-ranked in compound results, individualMoNAScore ≥ 0.9, no MoNA library score for other candidates), level 3a (fair library match, individualMoNAScore ≥ 0.4), level 3b (known MS/MS match, at least three fragment match), level 3c (good in silico MS/MS match, annotation MS/MS similarity (annSimComp) ≥ 0.7), level 4a (good formula MS/MS match, top-ranked formula candidate, annSimForm ≥ 0.7, isotopic match (isoScore) ≥ 0.5, both scores at least 0.2 higher than next best-ranked candidate), level 4b (good formula isotopic pattern match, top-ranked formula candidate, isoScore ≥ 0.9, score at least 0.2 higher than the next best-ranked candidate) and level 5 (nothing of the abovementioned criteria match).

## Results and Discussion

### Establishing a Dual-Column Approach for Combined Exposure and Effect Analysis

#### Selection of Columns and Eluents

**Figure 2:**
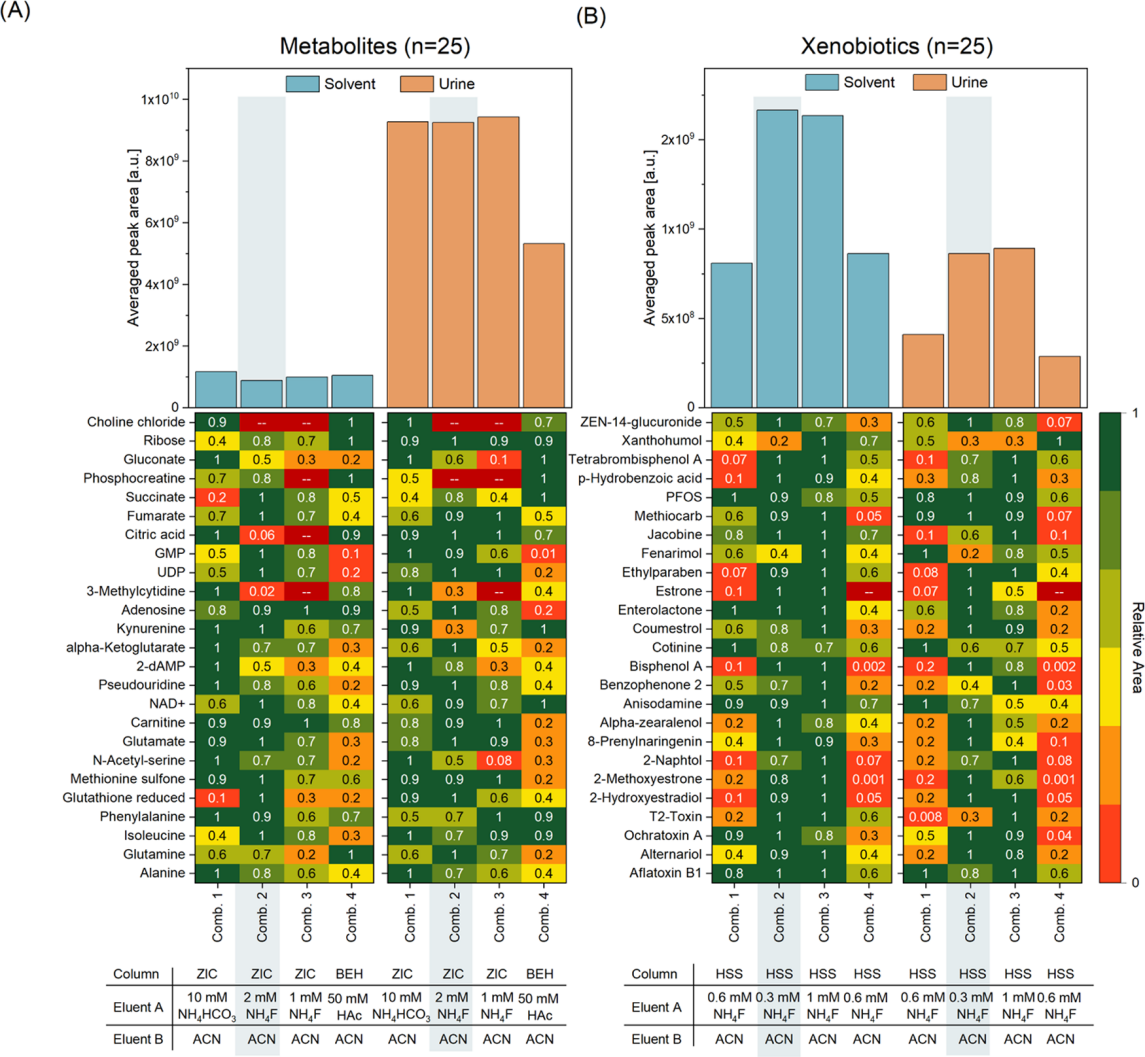
Comparison of the averaged peak areas of selected representative endogenous metabolites (*n* = 25) (A) and xenobiotics, including estrogen hormone metabolites (*n* = 25) (B) in solvent and urine, including a detailed overview of the averaged peak areas) relative to the highest peak area of the individual molecule from replicate injections of the samples areas (*n* = 4 (Comb. 4, Comb. 1); *n* = 6 (Comb.1, Comb. 2). The best overall combination is shaded in gray. Different columns, namely Acquity HSS T3 (HSS), SeQuant ZIC-pHILIC (ZIC), Acquity BEH Amide (BEH), and eluents, namely 0.3/0.6/1/2 mM NH_4_F in H_2_O (0.3/0.6/1/2 mM NH_4_F), 10 mM NH_4_HCO_3_ (pH 9.2) in H_2_O/ACN (9:1, v/v) (10 mM NH_4_HCO_3_), and 50 mM CH_3_COONH_4_ (pH 6) in H_2_O (50 mM HAc) were tested.

In optimization experiments, different eluent–column combinations were tested for the best overall performance and compatibility. Only eluents with a basic (pH 9.2) to slightly acidic pH (pH 6) were combined with NH_4_F to avoid the formation of hydrofluoric acid. For a representative selection of selected compounds (25 metabolites on the HILIC column and 25 xenobiotics and human estrogen metabolites on the RP column), the averaged peak area (*n* = 4–6) in a matrix-matched standard (urine) and a solvent standard both at a medium concentration level (level 6) were compared between the combinations (Figure 2 and Table S4). Peak areas were normalized to the best combination for each analyte to simplify the comparison. Endogenous metabolites are frequently naturally present in urine at high abundances. Therefore, the peak areas in the standard-spiked urine increased compared to the solvent standards. The observed signal was mostly decreased for xenobiotics due to signal suppression in the urine matrix. The averaged peak areas of the selected metabolites showed that the combination of 2 mM NH_4_F/ACN on a SeQuant ZIC-pHILIC column (HILIC) and 0.3 mM NH_4_F/ACN on an Acquity HSS T3 column is favorable (highest peak area) in the solvent. However, the difference between the three others is only minor, with combination 2 reaching about 85% of the average peak area of combination 1. In urine, the average peak area of combination 4, which uses an Acquity BEH amide column instead of a SeQuant ZIC-pHILIC column, is only about half of the others. Except for combination 4, all eluent–column combinations performed similarly, but with combinations 2 and 3, both using NH_4_F as an additive, up to four metabolites (choline, phosphocreatine, citric acid, 3-methylcytidine) were not detectable at the chosen concentration level. Therefore the SeQuantZIC-pHILIC column with a basic NH_4_CO_3_ buffer based on Schwaiger et al.^28^ seemed to be the best choice regarding endogenous metabolites excluding estrogen hormones. When xenobiotics and endogenous estrogen metabolites were investigated, however, the additive NH_4_F clearly outperformed the basic buffer. The average peak area in urine and solvent of this group nearly doubled when NH_4_F was used. Since xenobiotics are generally less concentrated than metabolites in real-life samples, we decided to use this additive to boost their sensitivity and finally selected the combination with 2 mM NH_4_F as the aqueous HILIC eluent and 0.3 mM NH_4_F as the aqueous RP column eluent due to better overall performance.

### Long-Term Stability of the LC-MS Setup

**Figure 3:**
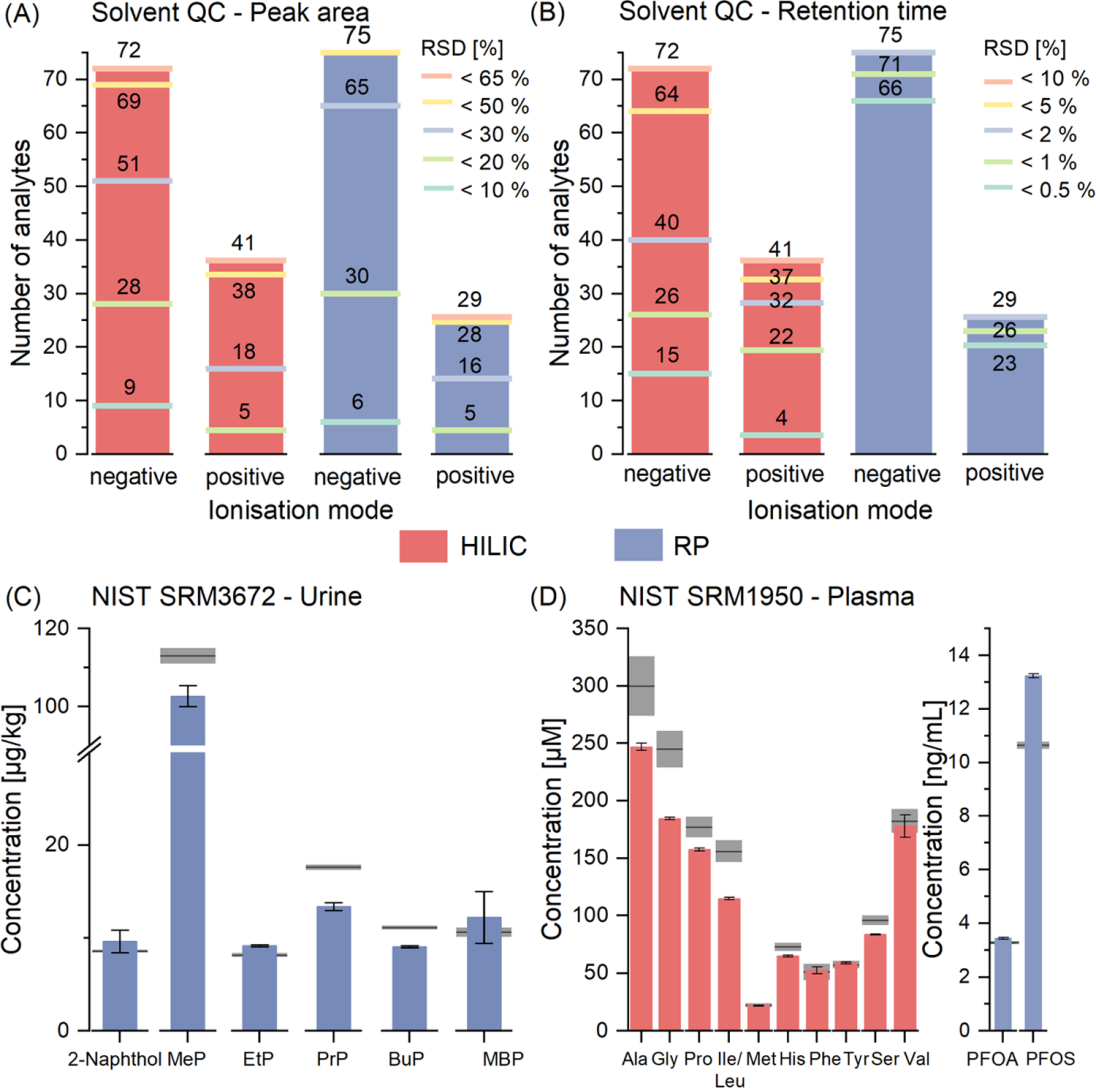
Repeatability and accuracy in a solvent QC sample (1 μM for metabolites and xenobiotics at approximately 100 ng/mL) are reported as the number of analytes within a certain relative standard deviation of the (A) peak area and (B) retention time. The lines mark the thresholds and the labels indicate how many analytes fall below a respective RSD percentage. Only the most abundant ion species was considered for the respective column. (C) Analytes reported in SRM3672 and (D) in SRM1950 with the gray line indicating the certified reference value and the expanded uncertainty according to the certificate of analysis.

A solvent QC sample containing endogenous metabolites at 1 μM (median of 186 ng/mL) and xenobiotics including human estrogens at approximately 100 ng/mL was injected throughout the sequence (*n* = 11), spanning over a period of 40 h. Only the most abundant ion species were considered. The peaks from the HILIC column were evaluated for assessing endogenous metabolites and the peaks from the reversed-phase column for evaluating xenobiotics and human estrogens. At the chosen concentration level, 113 out of the 146 metabolites (77%) were retained with a retention time bigger than 1 min on the HILIC column and 104 out of the 106 xenobiotics and human estrogens (98%) with retention on the RP column (retention time > 0.8 min) were detectable. Four analytes were not detectable even at higher levels. The relative standard deviation (RSD) was calculated for the peak area and the retention of all detectable molecules at this level (Figure 3A,B). The median RSDs of the area were 22 and 31% in the negative and positive ionization modes, respectively. The difference between peaks from the HILIC and RP separation was minor, but in the positive ionization mode, the relative standard deviation of the area was generally bigger. Regarding retention time, the median RSD was 0.4% for the negative mode and 0.7% for the positive mode. Xenobiotics’ times were more stable with a median of 0.3 compared to 1.3% for the endogenous metabolites. The retention time of some endogenous phosphates and acids, analyzed mainly in the negative ionization mode, was shifted significantly during the sequence as the buffer capacity of the NH_4_F eluent was low. The variation of the retention time and area of the most abundant ion species for the respective analytes is also presented in quality control charts (Figure S12).

Pooled quality control samples of both sample sets were measured several times throughout the measurement (Figure S2). The relative standard deviation of the retention time and the normalized peak area was calculated for highly diverse analytes. This included bisphenol A (BPA), daidzein, enterodiol, glycitein, methylparaben, and triclosan in the pooled urine of the Nigerian samples (*n* = 9) and BPA, daidzein, nonylphenol and p-hydroxybenzoic acid in the pooled urine of the Austrian samples (n=5). In addition, four metabolites (tryptophan, uracil, fumaric acid, gluconate) were investigated. The relative standard deviation of the retention time was <1.5% for all analytes except for fumaric acid (about 2.8%). The relative standard deviation of the normalized area was <20% for all six analytes except for BPA (22%), which was present at a low concentration around the LOD in the Austrian pooled urine sample. The variation tended to be lower if the corresponding ^13^C-labeled compound was available for compound-specific internal standardization (e.g., for methylparaben) as compared to surrogate internal standardization. A PCA plot of the Austrian and Nigerian pooled QC sample respectively shows clustering of the respective pooled urine sample (Figure S13).

### Quantification of Reference Materials

**Figure 4:**
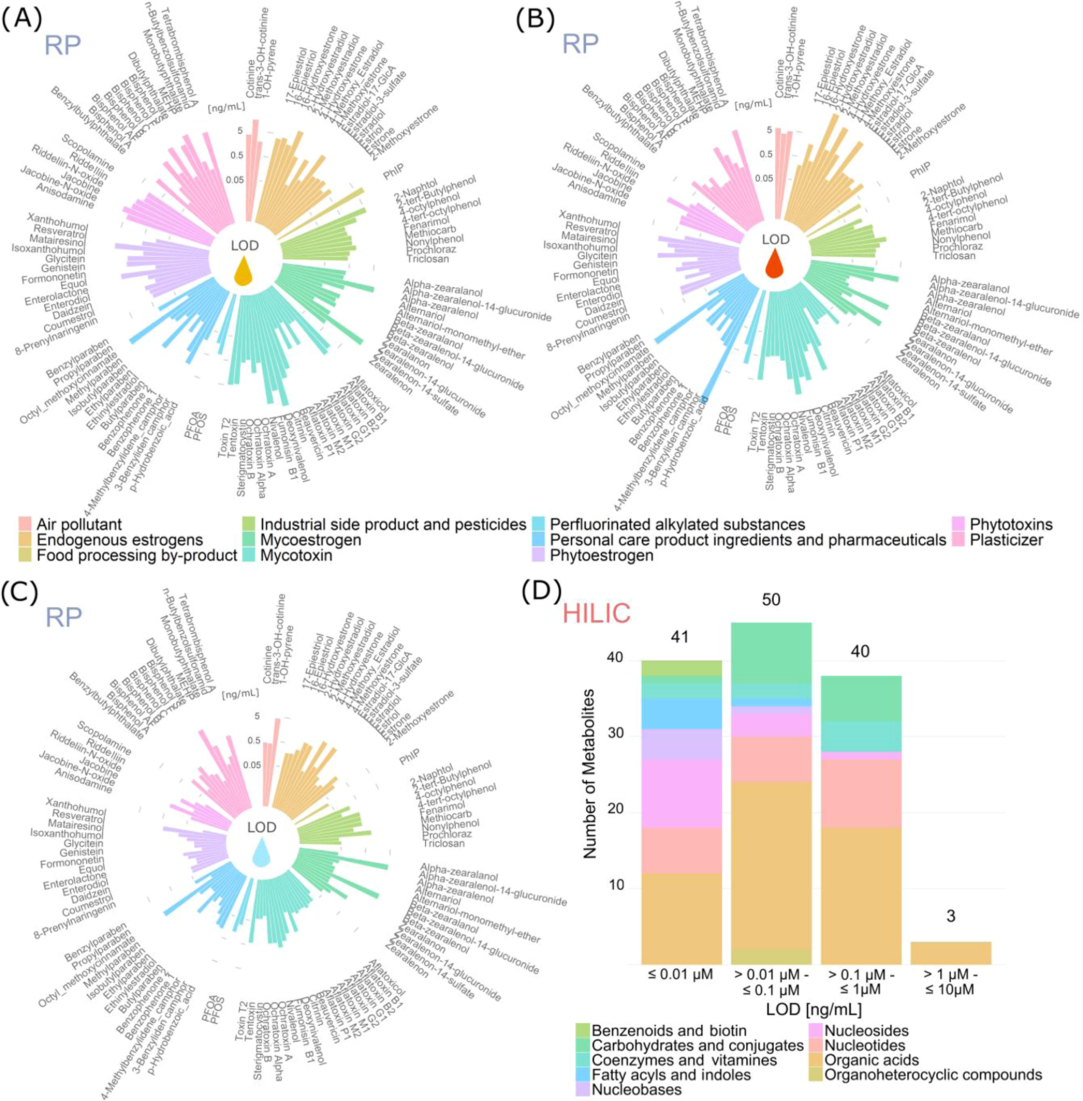
Limit of detection (LOD) of representative xenobiotics and estrogens in urine (A), plasma (B), and neat solvent (C) sorted by compound classification. The values were plotted on a logarithmic scale. (D) Number of endogenous metabolites in a specific LOD range. The color indicates the category to which the analytes can be classified to.

The limits of detection for the xenobiotics and human estrogens ranged from 0.01 to 5.7 ng/mL with a median of 0.08 ng/mL in the solvent (Table S5 and Figure 4C). In the matrix, the median LOD increased to 0.7 (urine, Figure 4A) and 0.5 (plasma, Figure 4B) most likely due to matrix effects and matrix interferences. Phytoestrogens like daidzein and genistein and personal care product ingredients, e.g., parabens, exhibited the lowest LODs. The LODs were, for most xenobiotics and human estrogens, sufficient to detect them in averagely contaminated samples considering concentration levels from published human biomonitoring studies (Table S21).

Six analytes for which certified reference values are available in SRM3672 (Organic Contaminants in Smoker’s Urine) were detected and quantified (Table S13). As the values stated in the certificate were total analyte concentrations after hydrolysis, β-glucuronidase/arylsulfatase-treated reference urine was analyzed. The relative error compared to the reference value was <20% for all of these (Figure 3C). 1-OH-pyrene and MEHP were not detected, although they were present in smokers’ urine, due to their low concentration below method’s quantification limits. In addition to several amino acids, PFOA and PFOS were quantified in SRM1950 (Metabolites in Frozen Human Plasma) and compared to the certificate (Figure 3D and Table S14). Threonine was not evaluated as it coeluted with homoserine; therefore, only a combined value for both amino acids was available. Isoleucine and leucine were not baseline separated as well, but for both molecules, reference values were given. Therefore the sum of both concentrations was compared. The relative error was <26% for all amino acids, whereby the recoveries were in general lower for the high-abundance amino acids (glycine (75%), alanine (82%), isoleucine/leucine (74%)), possibly due to saturation effects as the values were outside of the calibration range (Figure 3D). The samples were injected undiluted to ensure high detectability of less-concentrated xenobiotics despite possible saturation issues for the endogenous metabolites at higher concentrations. The relative errors of PFOA and PFOS were even <5 and <20%, respectively. In general, the determined values were in good agreement with the reference values of both SRMs, especially given the extensive scope of the new workflow with recoveries ranging from 74 to 124% in SRM1950 and recoveries between 81 and 115% in SRM3672 (Figure 3C). These recoveries are similar to values reported in the literature for SRM3672 by Karthikraj et al.^42^ (80.5–105%) and Zhu et al.^43^ (80–111%).

In the reference materials, several additional metabolites and xenobiotics were quantified (Figure S3 and Table S16). In SRM3672, 18 xenobiotics and two estrogens were detected. SRM1950 was contaminated with 17 xenobiotics, with nine of them being detected in both reference materials, including personal care product ingredients (methylparaben, propylparaben, *p*-hydroxybenzoic acid, benzophenone-1), phytoestrogens (genistein, daidzein), smoking markers (cotinine, trans-3-OH-cotinine), and an industrial side product (2-naphthol). In SRM3672, additional phytoestrogens (e.g., enterolactone, enterodiol, daidzein, glycitein), a plasticizer (monobutyl phthalate), and butylparaben were observed, whereas, in SRM1950, scopolamine, a phytotoxin, perfluorinated substances (PFOA, PFOS), industrial side products (nonylphenol, 4-*tert*-octylphenol) and other plasticizers (BPS, BPA) were found. The quantified metabolome comprised 48 (SRM1950) and 61 (SRM3672) additional compounds, respectively. Smoker’s urine was not enzyme-treated for this evaluation. Mainly amino acids, nucleobases, and nucleosides were quantified in these samples. The concentrations ranged over four orders of magnitude for metabolites in the urine plasma reference material. This range spanned even over six orders of magnitude when expanding to exogenous contaminants. In the urine reference material, concentrations were less variable covering three orders of magnitude.

### Analytical Parameters

The linear dynamic range was highly variable and depended on the investigated molecule (Table S19). About 10% of the xenobiotics and human estrogens had a linear range spanning five orders of magnitude. Half of them covered a range of four and approximately a third a range of three orders of magnitude in the solvent. Matrix matching typically reduced the dynamic range by about one order of magnitude due to matrix effects, as the LOD of the xenobiotics and human estrogens in the matrix was generally lower.

The accuracy in a solvent standard compared to the expected concentration at level 6 ranged in solvent between 83 and 123% with an average of 104% for xenobiotics including human estrogens and 81 and 119% with an average of 101% for endogenous metabolites (Table S19/Table S20). Furthermore, the extraction recoveries were evaluated in spiked urine and plasma samples (Table S19/Table S20). The median recovery of xenobiotics and human estrogens was 97% in urine and 103% in plasma, whereas for metabolites, these figures were determined to be 97 and 99%, respectively. Only 10% of the analytes in urine and 14% in plasma had a recovery below 80%. The recoveries were above 120% for about 1% of compounds in urine and 3% in plasma. Several analytes, especially endogenous metabolites, were already present in high quantities in the nonfortified urine and plasma. Hence, the extraction recovery was not estimated for all compounds.

The solvent LODs of endogenous metabolites ranged from 0.001 μM (0.2 ng/mL) to 6.6 μM (1068 ng/mL) with a median of 0.04 μM (8 ng/mL) (Table S6 and Figure 4D). The LOD values were estimated for 134 analytes, including several jointly evaluated isomers. Due to their high natural abundance, the LOD of several endogenous metabolites was not estimated in the human matrices. About one-third of all compounds showed LODs <0.01 μM (approximately 1.9 ng/mL) and about two-thirds <0.1 μM (approximately 19 ng/mL) allowing for the straightforward (semi-) quantitative assessment of the metabolome in most biological systems. The reference standards spanned five orders of magnitude, from 0.001 to 10 μM. However, most metabolites were not detectable at the lower calibration level (0.001–0.01 μM), limiting the linear dynamic range to four (45%), three (29%) or even less (8%) orders of magnitude (Table S20).

### Limitations

Out of the 145 endogenous metabolites in the standard mix, five (1-methylnicotinamide, thiamine, choline, spermine, and spermidine) were not detectable even at the highest concentration level (10 μM, approximately 1860 ng/mL). Isomers were not separated in some cases. These included 2-/3-phosphoglyceric acid, citric/isocitric acid, homoserine/threonine, and isoguanosine/guanosine. The hexoses fructose, galactose, mannose, glucose, and inositol were not distinguishable, as well as their phosphates (fructose-6-phosphate, glucose-1-phosphate, and glucose-6-phosphate) as they were coeluting and therefore the peaks were not baseline separated. Pentose-phosphates, ribose-5-phosphate, and ribulose-5-phosphate were not baseline separated too. For arginine and palmitic acid, no satisfactory linear regression was achieved, most likely due to severe carryover effects. The setup of the dual-column approach is simple but requires two independent pumps. Thus, our dual-column approach might not be transferable to another laboratory without acquiring specific equipment before. A limitation of our study was the selection of real-life samples as both sample sets are not matched in size, demographic background, and storage duration. However, these samples still have the power to showcase the unique capacities of the developed approach to detect various chemicals from endogenous and exogenous sources. Moreover, only a single reference material was analyzed as the experimental sample for plasma. Nonetheless, we could identify over 60 chemicals in this plasma sample showing highly convincing performance for this matrix.

The combination of the column effluents before introduction to the MS increases the amount of coeluting compounds, potentially causing elevated matrix effects. At the same time, the sample is diluted as the flow is doubled before the analysis.

Internal standard normalization was applied for quantification. Since the set of labeled standards was limited, partially surrogate internal standards were applied reducing the reliability of normalization for these analytes.

The concentrations in the urine samples were not normalized to the creatinine level, thus the varying matrix dilution might cause varying matrix effects impacting the analysis

### Application in Biomonitoring Studies from Europe and Sub-Saharan Africa

**Figure 5:**
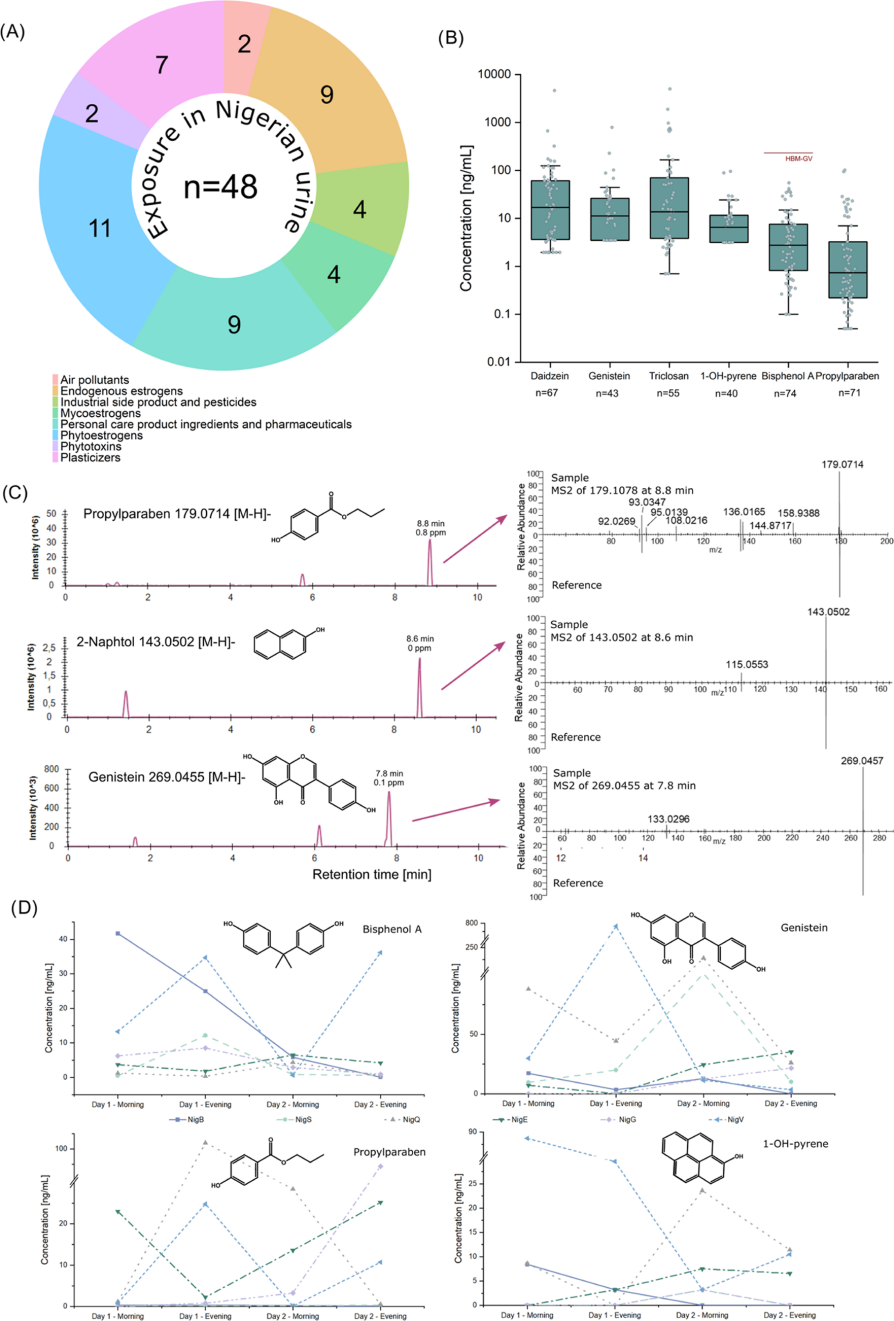
Detection of xenobiotics and endogenous estrogens in Nigerian samples. (A) Variety of observed xenobiotics including human estrogens and their classification. (B) Individual concentrations of selected analytes that have been detected in > 50% of the samples (*n* = 77). The human biomonitoring (HBM) guidance value for BPA^45^ was added in red. (C) Extracted ion chromatograms (XICs) of selected analytes, including corresponding MS2 spectra of experimental samples and authentic reference standards. (D) Variation of analyte concentration in six individuals for selected xenobiotics demonstrates severe exposure dynamics and the need for longitudinal sampling design.

The established analysis pipeline was applied in proof-of-principle studies of two different urine sample sets from geographically different areas, Austria and Nigeria. Both studies included several individuals who donated samples at four different time points. In the Austrian samples, 17 different xenobiotics belonging to four compound categories were identified (Figure S5A/B and Table S10). Most exposures were either personal care product ingredients or phytoestrogens. The Nigerian urine samples were contaminated with more than twice as many chemicals (*n* = 48) coming from diverse sources (Figure 5A and Table S9), but personal care product ingredients and phytoestrogens were still the dominant classes. Concentrations covered five orders of magnitude from the sub-ppb to the ppm range. The Nigerian samples were contaminated with a broader diversity of chemicals, including air pollutants (1-OH-pyrene), mycoestrogens (ZEN, AME), and industrial side products (2-naphthol) at higher concentration levels. In particular, the maximum values were frequently 10–100-times higher than the Austrian samples. However, especially the Austrian dataset was small and homogenous compared to the Nigerian dataset. Moreover, the demographic data of the individuals involved in both studies and storage times varied and the stability of analytes was not specifically assessed in this work. Therefore, the available data are not sufficiently representative to estimate population-wide exposure levels. However, the results highlight the potential of the dual-column approach to capture environmental contaminants in different urine samples. The Austrian samples were considered a low-exposure scenario due to strict regulation and enforcement on the use/tolerance of e.g., pesticides and mycotoxins, whereas the Nigerian samples were regarded as high-exposure samples as often food/environmental safety regulations are lacking or not adequately enforced. Each detected xenobiotic was, on average, detected in 31 out of 77 Nigerian samples. Four analytes (enterolactone, BPA, nonylphenol, and propylparaben) were present in >90% of all samples (Figure 5B). In the Austrian samples, two-thirds were positive for daidzein, 4-tert-octylphenol, BPA, and propylparaben. In addition, all samples contained nonylphenol and dibutyl phthalate. MS2 spectra further supported identification (Figure 5C), although for all analytes discovered here reference standards were used for retention time confirmation (level 1 identifications). The number and level of the detected phytoestrogens were restricted due to the regulated diet with no vegetable and fruit intake except for a smoothie on day 2 before the Austrian study and on the first two study days. Approximately 70 diverse endogenous metabolites were quantified in the urine samples of both studies (Tables S11 and S12). Thirteen individuals completed the full longitudinal sampling with four time points. The variation over two days is exemplified for six individuals and analytes of distinct origins, including the plasticizer bisphenol A, the phytoestrogen genistein, the personal care product ingredient propylparaben and the air pollutant 1-OH-pyrene in Figure 5D. The concentrations spanned over three orders of magnitude within the individual sample. Genistein and propylparaben differed vastly between the time points in particular. The intraindividual variation was like the interindividual variation demonstrating the importance of longitudinal sampling for exposure assessment of several participants. The diversity and high dynamics of the exposome even within the same individual were described previously^44^ and further supported the need for time-resolved testing to capture dynamic exposure in spot urine samples or 24 h urine.

A Spearman correlation matrix was created to showcase the power of our approach for deriving possible impacts of exogenous exposures on the endogenous metabolites that are of high interest for further thorough mechanistic studies. Various significant correlations between xenobiotics and also between xenobiotics and metabolites were demonstrated (Figure S4). Mainly analytes detected in many samples, e.g., monobutyl phthalate, ethylparaben, MEHP, benzophenone, and enterolactone, yielded significant correlations, probably due to the higher statistical power. High-correlation coefficients were observed for monobutyl phthalate and MEHP (0.72), ethylparaben and *p*-hydroxybenzoic acid (0.64), benzophenone-1 and enterolactone (0.74), and propylparaben and methylparaben (0.59). MEHP and monobutyl phthalate are both urinary biomarkers of phthalate exposure.^46^ The preservatives ethylparaben and p-hydroxybenzoic acid were likely to be an ingredient in the same type of personal care products and also methylparaben and ethylparaben may be present in similar products. Enterolactone, a biotransformation product of plant lignans originating from, e.g., flaxseeds and sesame,^47^ and the urinary biotransformation product of benzophenone-3, benzophenone-1, a UV-filter used in cosmetics,^48^ clearly have different sources. However, both are hormonally active and have a shared mechanism concerning obesity antagonism. This was demonstrated to be associated with late onset of puberty in girls.^49^

The link between external exposures and the ensuing disturbance of the internal metabolome is one step to elucidating disease development, which is part of the broad scope of exposome research. With our approach, connections between xenobiotics and metabolites were indicated. A strong correlation between ethylparaben and the carboxylic acids, fumaric acid (0.68) and malic acid (0.67) and the amino acid alanine (0.62) were uncovered. Alanine (0.64), fumaric acid (0.63) and malic acid (0.6) were connected to benzophenone-1, too. The matrix also strongly correlated monobutyl phthalate and aspartic acid (0.66). These correlations highlight potentially interesting links between the exposome and the metabolome and might be seen as a starting point for deciphering causal connections. Additional, in-depth experiments will be necessary to prove these links in future ExWAS investigations.

However, in the literature some exposed correlations have already been reported. Phthalate exposure was associated with altered carnitine levels and changes in metabolites associated with amino acid metabolism in the urine of Chinese men.^50^ In our study, carnitine, propionyl carnitine, and several amino acids were also correlated with monobutyl phthalate. Pathway analysis was performed for significantly correlated metabolites with monobutyl phthalate (Figure S6). The results revealed a potentially strong impact on alanine, aspartate, and glutamate metabolism, arginine biosynthesis, and the citrate cycle. The pathway analysis of ethylparaben-associated metabolites showed similar results, with arginine biosynthesis and alanine, aspartate, and glutamate metabolism being the most affected pathways (Figure S7). These two pathways were also the most affected along with benzophenone-1 correlated metabolites (Figure S8). The xenobiotics benzophenone-1, monobutyl phthalate, and ethylparaben exhibited a moderate correlation (correlation coefficient between 0.5 and 0.64) among each other, therefore, the disturbance in amino acid metabolism might not be triggered by a single compound but rather by a mixture of different chemicals. Further investigations are needed to support our preliminary observations.

Recent studies demonstrated the feasibility of exposome-wide association studies (ExWAS), e.g., extensive effect biomarker and biomarker discovery of air pollutant exposure^51^ and linking metabolic profiling and exposure to perfluoroalkyl substances.^52^ However, several independent measurements were required to capture the metabolome and chemical exposome, increasing the measurement time compared to our 15 min LC-HRMS/MS run covering both polar metabolites and the primarily non-polar xenobiotics. Especially, molecules with high vapor pressure and low boiling/melting point were not accessible with LC-MS technology. Therefore, GC-MS would need to be integrated to further extend coverage.^53,54^ Although our fast LC-MS approach might be advantageous regarding run time, the vast concentration difference between endogenous metabolites and environmental contaminants^30^ hamper their simultaneous measurement as several endogenous metabolites were close to the detector saturation and less-abundant xenobiotics still need higher sensitivity. In particular, quantification posed a challenge, as calibration ranges were partially exceeded. Internal standard correction eased linearity issues especially at high concentrations as it compensates for increasing ion suppression and detector saturation.

### Suspect Screening in Biological Samples Obtained from Nigerian Women

Raw data from four pooled Nigerian urine samples with iterative MS2 exclusion lists were processed for suspect screening to demonstrate the workflow’s suitability for suspect and nontargeted screening/analysis (NTS/NTA). On average, 14,126 and 18,408 features were picked in each sample in the negative mode and positive mode, respectively; 16,288 negative features in 4590 groups remained after removing features present in the blank and applying an intensity and replicate abundance filter. In the positive ionization mode, 21,384 features in 5937 groups remained after filtering. Features were included if they were present in at least 3 out of 4 replicates. Therefore the feature number after the filter application was higher than that in the individual sample. A match with the suspect list was found for 706 (positive) and 749 (negative) peaks. The suspect list contained several isomers. Therefore in some cases, various molecules were suggested as an annotation. Altogether, 1238 different compounds, partly observed in both ionization modes, were proposed as potential annotations for the feature groups. Identification levels 1–4 were established for 52% (370) and 58% (435) in positive and negative ionization modes, respectively (Tables S17 and S18). MS2 matches (level ≥ 3c) were obtained for 187 (positive mode) and 190 (negative mode) feature groups. The annotation of 377 feature groups in both ionization modes with an identification level of at least 3c was successful, consequently showcasing the workflow’s ability to detect analytes without available reference standards and capture additional compounds potentially present in the samples.

## Conclusions

The presented workflow facilitates the rapid and simultaneous exploration of complex environmental exposures and their effect on the human metabolome. Despite issues due to the wide concentration range, the quantification of several endogenous metabolites and exogenous chemicals acquired simultaneously in one short LC-MS/MS run succeeded and preliminary correlations between metabolites and chemicals were revealed. Consequently, the potential effects of specific exposures on the metabolome were directly derived from exposure data in a unique way. However, further experiments are required to establish solid causal relationships. Combining two columns and both ionization modes in one single data file drastically decreased measurement time and simplified data evaluation and storage requirements. For deciphering the exposome, hundreds to thousands of samples will be needed to be analyzed. Therefore, the reduced analysis time opens up for so far unseen throughput in exposome-wide association studies for drawing reliable conclusions on the impact of environmental factors on disease development.

## Supporting Information Available

Supporting Information A: visualization of the diversity of selected model molecules, normalized peak areas of QC samples, concentrations of noncertified values in the reference material, Spearman correlation matrix, summary of detected xenobiotics in Austrian urine samples, pathway analyses, total ion chromatograms, summary of molecules in a multi-analyte mixture and their concentrations including internal standards, average peak areas of analytes for different eluent/column combinations, limits of detection and retention times of analytes, calibration parameters, and linear dynamic ranges and recoveries of analytes (PDF)
Supporting Information B: detailed results of Nigerian and Austrian urine analysis, concentrations of certified values in reference materials, results of suspect screening approach, creatinine levels of urine samples, selected extracted ion chromatograms, quality control charts, PCA plots, and peak areas of pooled QC sample over several injections (XLSX)
