# Supplementary material for: Integrated Exposomics/Metabolomics for Rapid Exposure and Effect Analyses
Source: JACS Au. 2022 Nov 7;2(11):2548–60. doi: 10.1021/jacsau.2c00433 (PMC9709941; doi:10.1021/jacsau.2c00433)
Supplement: Supplementary file 1 — au2c00433_si_001.pdf [file au2c00433_si_001.pdf]

Supporting Information

Integrated exposomics/metabolomics for rapid exposure and effect analyses

Mira Flasch[1,2], Veronika Fitz[2,3], Evelyn Rampler[3], Chibundu N. Ezekiel[4], Gunda Koellensperger[3,5], Benedikt Warth[1,5*]

[1]University of Vienna, Faculty of Chemistry, Department of Food Chemistry and Toxicology, Währinger Straße 38-40, 1090 Vienna, Austria

[2]University of Vienna, Vienna Doctoral School of Chemistry, Währinger Straße 42, 1090, Vienna, Austria

[3]University of Vienna, Faculty of Chemistry, Department of Analytical Chemistry, Währinger Straße 38-40, 1090 Vienna, Austria

[4]Department of Microbiology, Babcock University, Ilishan Remo, Ogun State, Nigeria

[5]Exposome Austria, Research Infrastructure and National EIRENE Hub, Austria

*benedikt.warth@univie.ac.at; +43 1 4277 70806

Table of Contents

Figure S1 Structural diversity of some of the model molecules involved in this study 3

Figure S2 Peak areas normalized to internal standard of the pooled QC sample from A) Nigerian urine and B) Austrian urine 4

Figure S3 Concentration of quantified A) xenobiotics and human estrogens (only analytes not described in the certificate) and B) endogenous metabolites present in SRM3672 (green) and SRM1950 (grey)……………………. 5

Figure S4 Spearman correlation matrix of analytes detected in at least 20 % of all samples. 6

Figure S5 Detection of xenobiotics in Austrian urine samples... 7

Figure S6 Results of pathway analysis of with monobutyl phthalate correlated metabolites 8

Figure S7 Results of pathway analysis of with ethylparaben correlated metabolites including for multiple testing corrected p-values.. 9

Figure S8 Results of pathway analysis of with benzophenone 1 correlated metabolites including for multiple testing corrected p-values 10

Figure S9 Total ion chromatograms of a solvent (A,D), a urine (B,E) and a plasma multi-analyte standard (std 6) in negative and positive mode 11

Figure S10 Total ion chromatograms of pooled quality control (QC) sample from Austria (A,C) and Nigeria (B,D) in negative and positive mode 12

Table S1 Summary of molecules (n=251) present in the multi-analyte mixture and available internal standards (n=15)……………………………. 13

Table S2 Analyte concentrations in the solvent multi-analyte standard 20

Table S3 Concentrations of ^{13}C-labeled analytes in the internal standard mix 25

Table S4 Average peak area of individual endogenous metabolites (analysed with the HILIC column) and xenobiotics/endogenous estrogen metabolites (analysed with the RP column) 26

Table S5 Limit of detection (LOD), retention time and the detected adducts (with the most abundant one in bold)……………. 29

Table S6 Limit of detection (LOD), retention time and the detected adducts................................ 33

Table S7 Calibration parameter of analytes in all three matrices and used normalisation method 38

Table S19 Linear dynamic range of solvent standards and matrix-matched standards, recovery of solvent standards and extraction recovery of spiked matrix standards 48

Table S20 Linear dynamic range of solvent standards and matrix-matched standards, recovery of solvent standards and extraction recovery of spiked matrix standards 52

Table S21 Limit of detection, retention time and the detected adducts in solvent and matrix....... 57

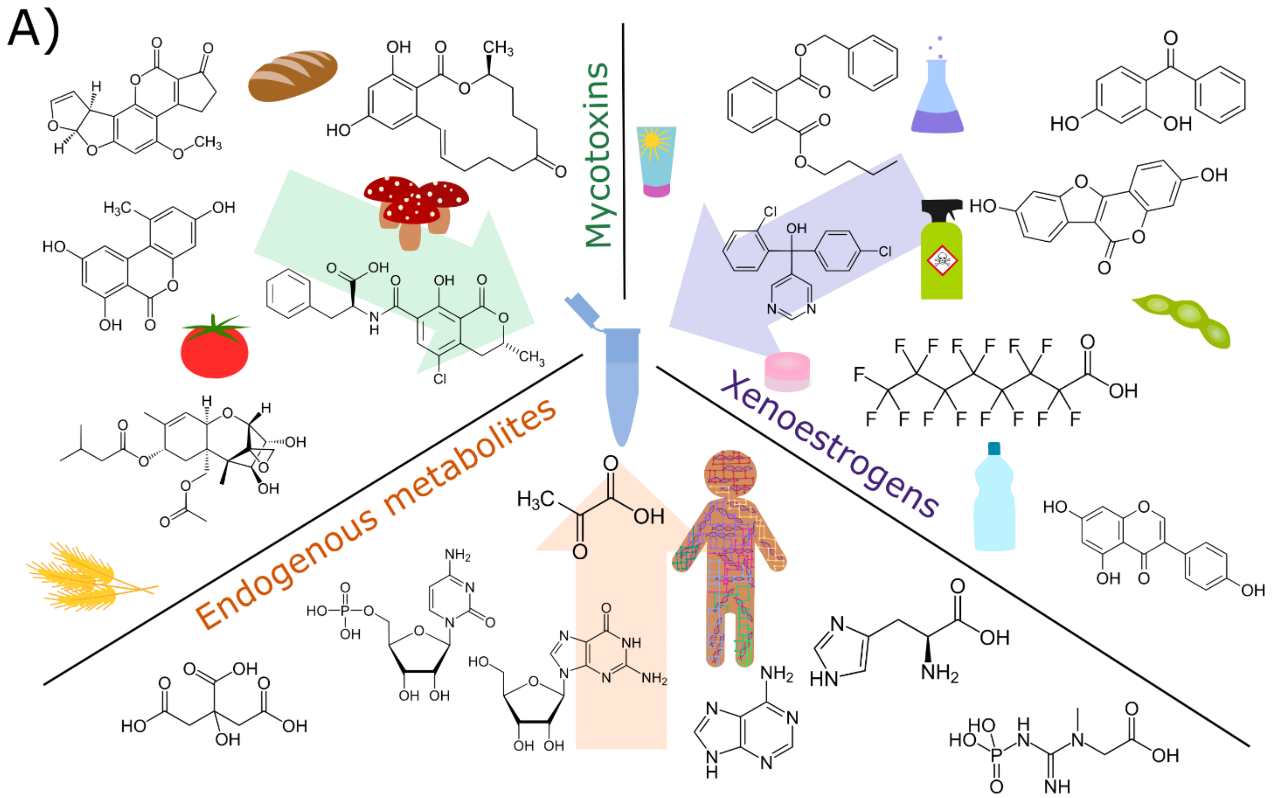

Figure S1 Structural diversity of some of the model molecules involved in this study

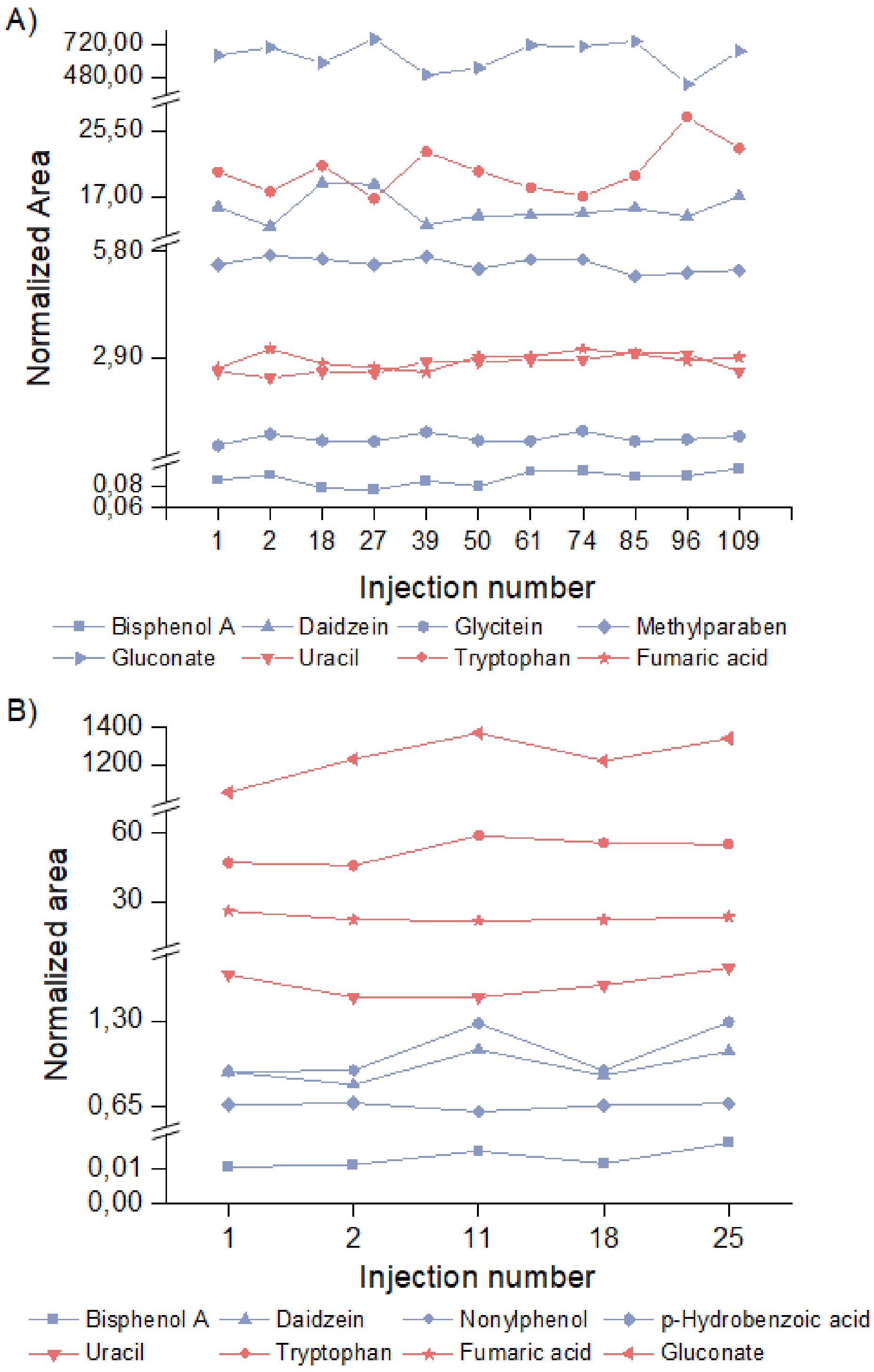

Figure S2 Peak areas normalized to internal standard of the pooled QC sample from A) Nigerian urine and B) Austrian urine over several injections within the sequence to demonstrate the high robustness and repeatability of the method

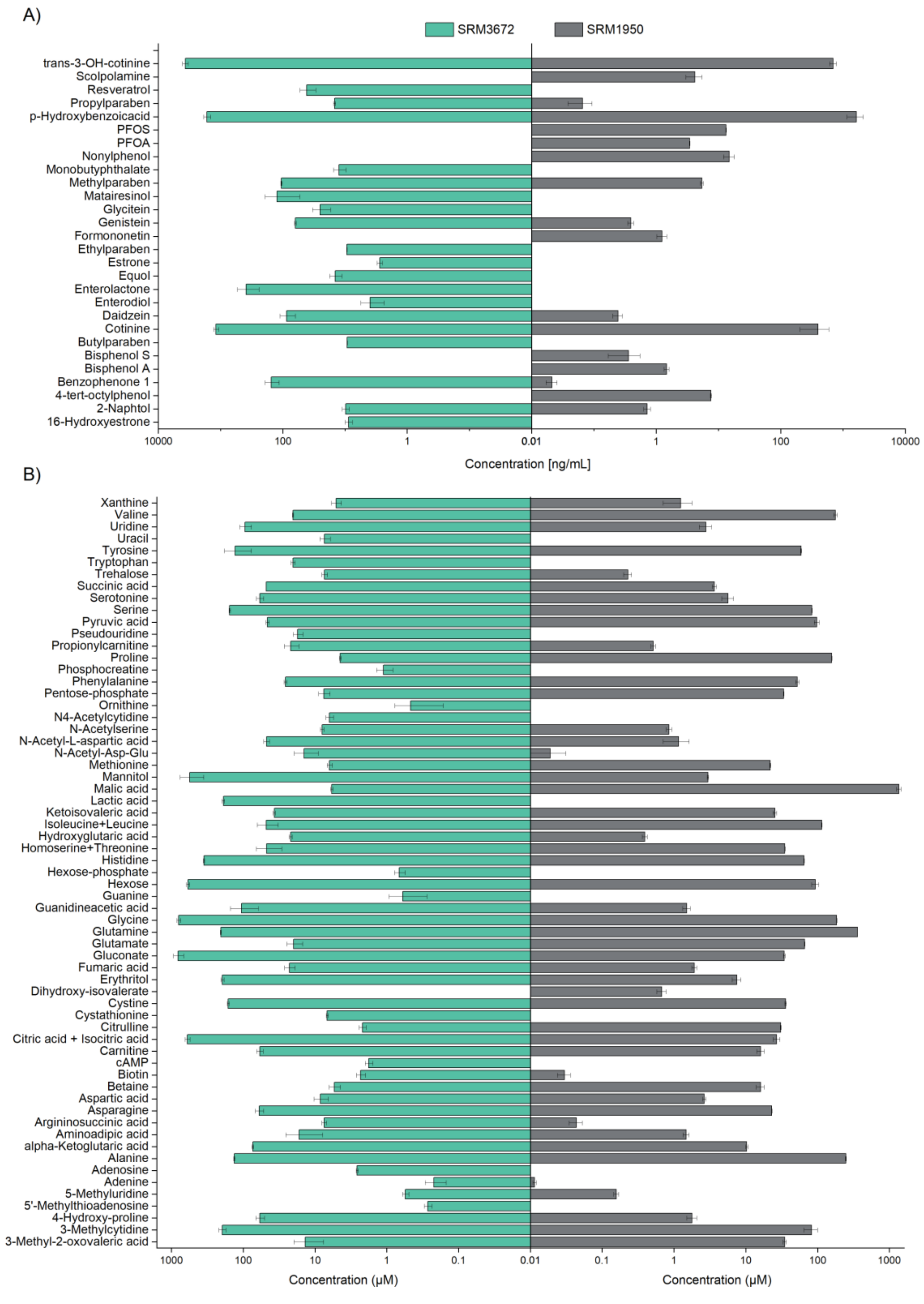

Figure S3 Concentration of quantified A) xenobiotics and human estrogens (only analytes not described in the certificate) and B) endogenous metabolites present in SRM3672 (green) and SRM1950 (grey)

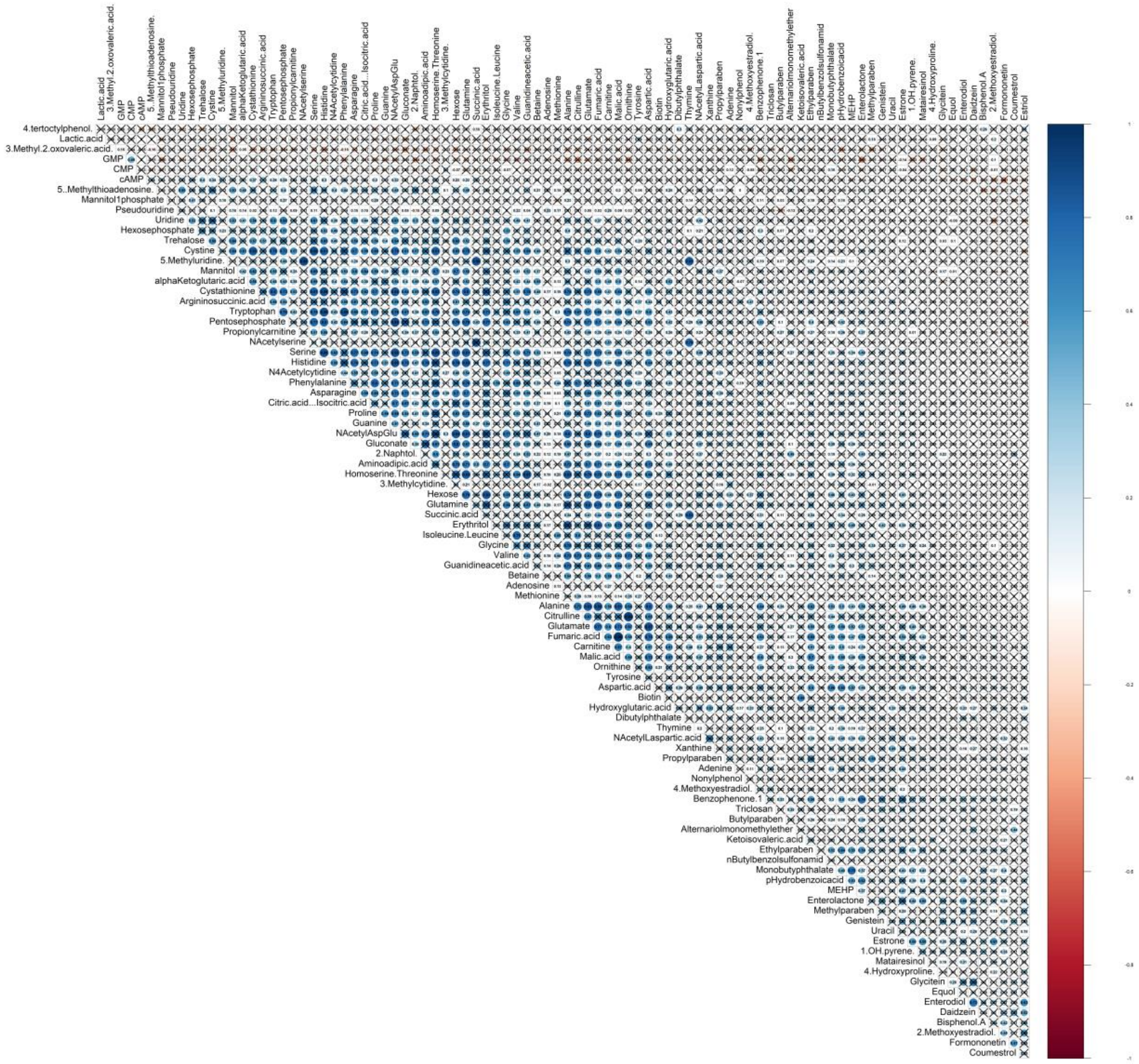

Figure S4 Spearman correlation matrix of analytes detected in at least 20 % of all samples. The significance level was tested and if higher than 0.01 the squares were crossed

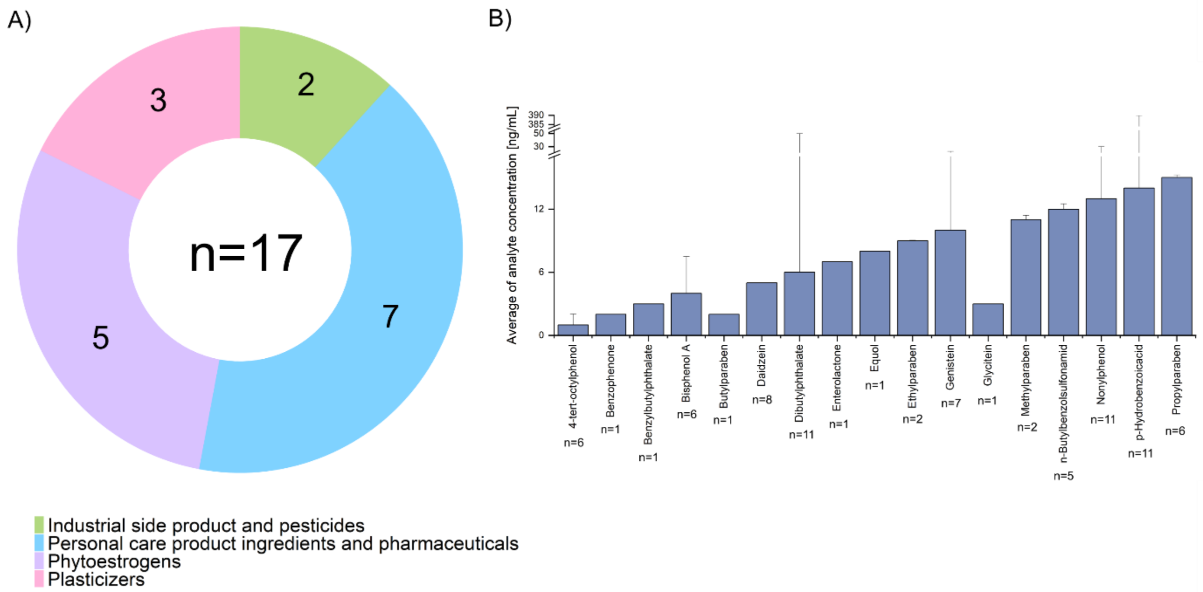

Figure S5 Detection of xenobiotics in Austrian urine samples. A) Variety of observed xenobiotics and their expected origin. B) Average concentration of in the samples detectable compounds with standard deviation

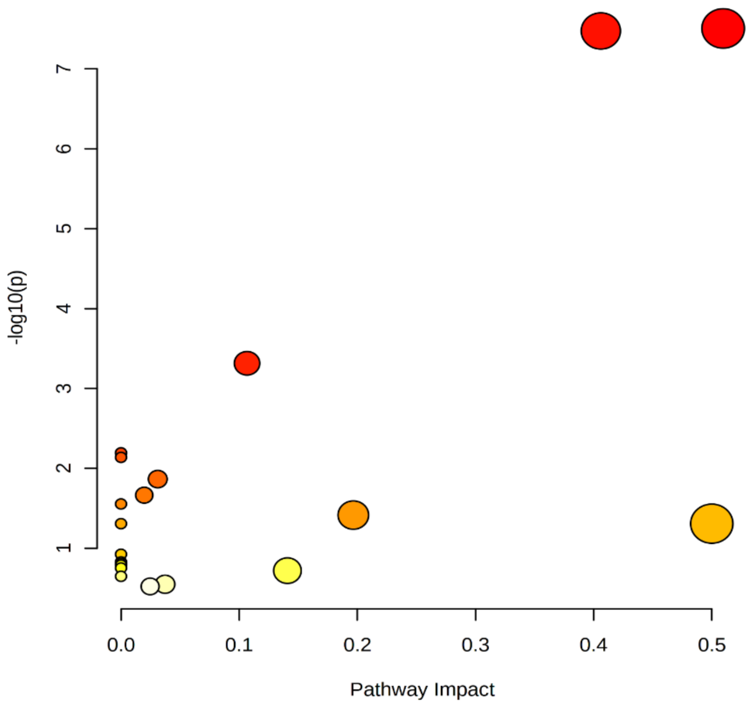

Pathway Name	Match Status	p	-log(p)	Holm p	FDR	Impact	Details
Alanine, aspartate and glutamate metabolism	6/28	3.1097E-8	7.5073	2.6121E-6	1.405E-6	0.50962	KEGG SMP SMP SMP
Arginine biosynthesis	5/14	3.3453E-8	7.4756	2.7766E-6	1.405E-6	0.40609	KEGG
Citrate cycle (TCA cycle)	3/20	4.8454E-4	3.3147	0.039733	0.013567	0.10666	KEGG SMP
Aminoacyl-tRNA biosynthesis	3/48	0.0064113	2.1931	0.51932	0.10214	0.0	KEGG

Figure S6 Results of pathway analysis of with monobutyl phthalate correlated metabolites including for multiple testing corrected p-values from MetaboAnalyst 5.0 (1)

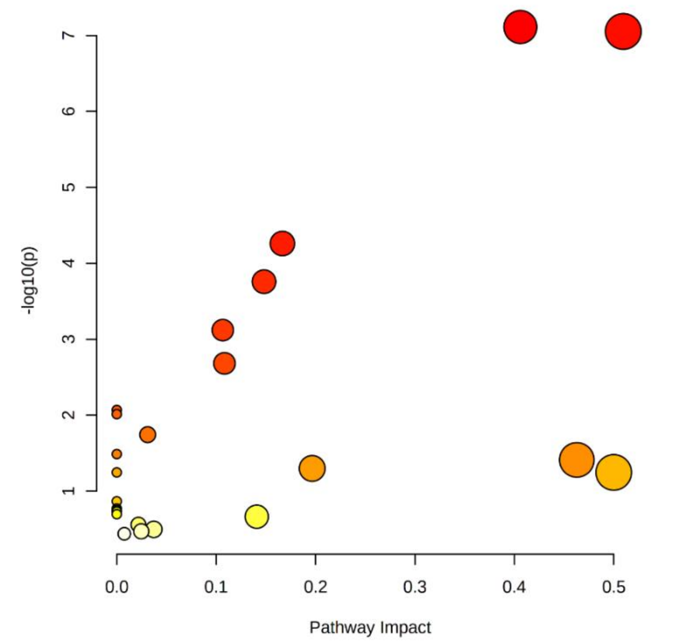

Pathway Name	Match Status	p	-log(p)	Holm p	FDR	Impact	Details
Arginine biosynthesis	5/14	7.7299E-8	7.1118	6.4931E-6	3.7167E-6	0.40609	KEGG
Alanine, aspartate and glutamate metabolism	6/28	8.8492E-8	7.0531	7.3449E-6	3.7167E-6	0.50962	KEGG SMP SMP SMP
Aminoacyl-tRNA biosynthesis	5/48	5.4905E-5	4.2604	0.0045022	0.0015374	0.16667	KEGG
Glyoxylate and dicarboxylate metabolism	4/32	1.7451E-4	3.7582	0.014135	0.0036647	0.14815	KEGG
Citrate cycle (TCA cycle)	3/20	7.5822E-4	3.1202	0.060657	0.012738	0.10666	KEGG SMP
Glutathione metabolism	3/28	0.0020793	2.6821	0.16426	0.02911	0.10839	KEGG SMP

Figure S7 Results of pathway analysis of with ethylparaben correlated metabolites including for multiple testing corrected p-values from MetaboAnalyst 5.0 (1)

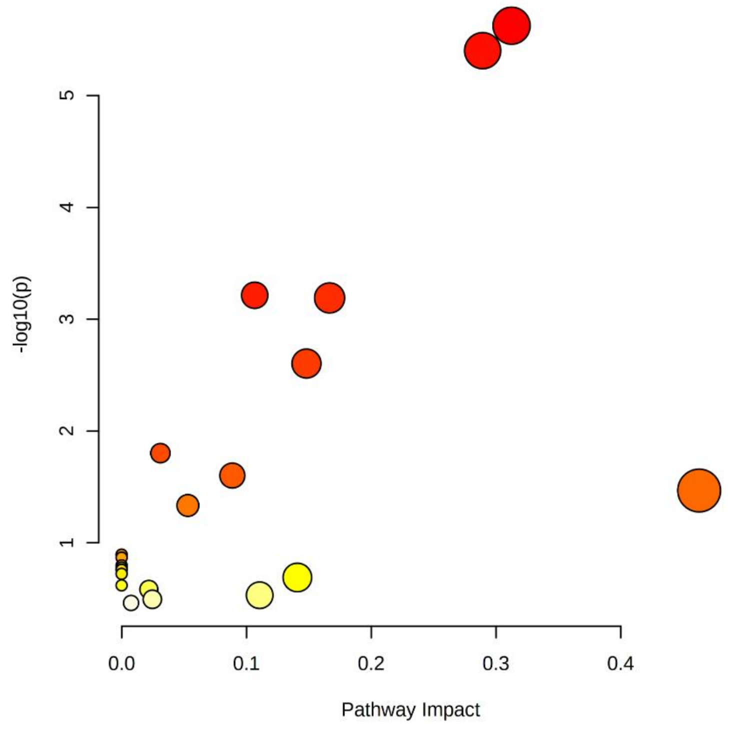

Pathway Name	Match Status	p	-log(p)	Holm p	FDR	Impact	Details
Alanine, aspartate and glutamate metabolism	5/28	2.3737E-6	5.6246	1.9939E-4	1.6677E-4	0.3125	KEGG SMP SMP SMP
Arginine biosynthesis	4/14	3.9707E-6	5.4011	3.2957E-4	1.6677E-4	0.28934	KEGG
Citrate cycle (TCA cycle)	3/20	6.1161E-4	3.2135	0.050152	0.013567	0.10666	KEGG SMP
Aminoacyl-tRNA biosynthesis	4/48	6.4607E-4	3.1897	0.052332	0.013567	0.16667	KEGG
Glyoxylate and dicarboxylate metabolism	3/32	0.0024951	2.6029	0.19961	0.041918	0.14815	KEGG

Figure S8 Results of pathway analysis of with benzophenone 1 correlated metabolites including for multiple testing corrected p-values from MetaboAnalyst 5.0 (1)

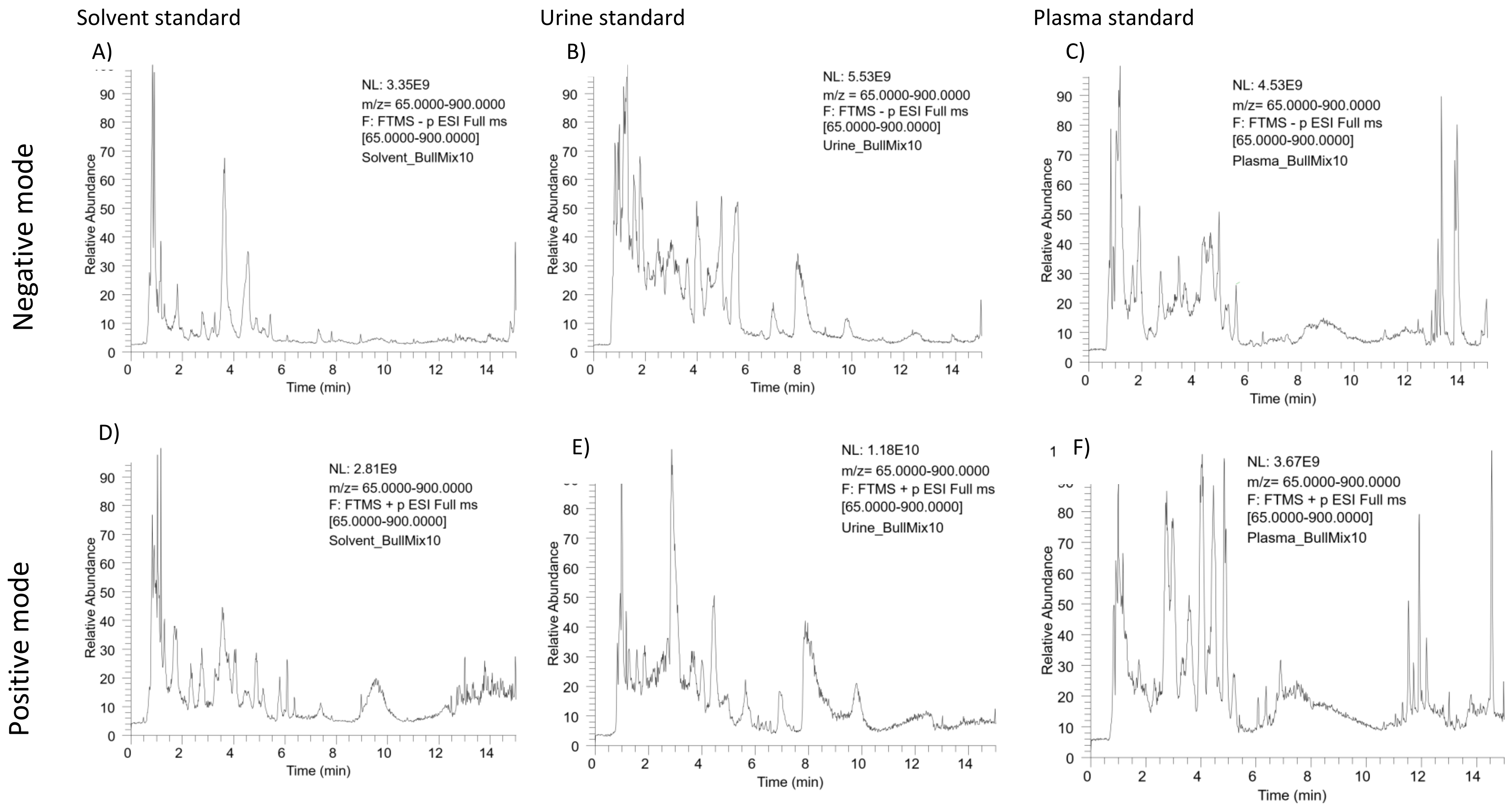

Figure S9 Total ion chromatograms of a solvent (A,D), a urine (B,E) and a plasma multi-analyte standard (std 6) in negative and positive mode

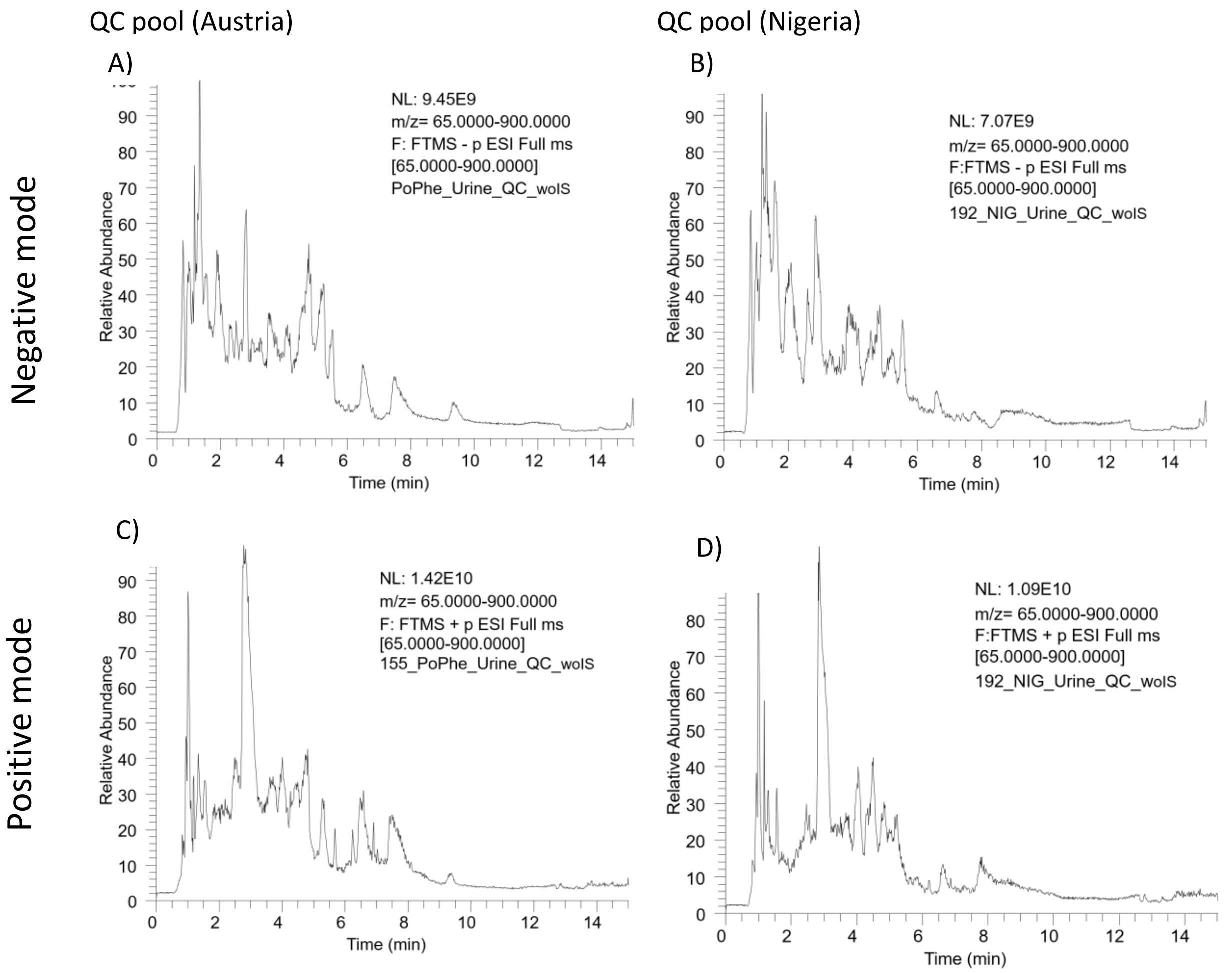

Figure S10 Total ion chromatograms of pooled quality control (QC) sample from Austria (A,C) and Nigeria (B,D) in negative and positive mode

Table S1 Summary of molecules (n=251) present in the multi-analyte mixture and available internal standards (n=15)

Compound	Molecular Formula	CAS number	Classification	Abbreviation
		Endogenous metabolites excluding human estrogens		
1-Methylhydantoin	C4H6N2O2	616-04-6	Organoheterocyclic compounds	Methylhydantoin
1-Methylnicotinamide	C7H8N2O	3106-60-3	Organoheterocyclic compounds	MNA
2-(Carbamoylamino)butanedioic acid (Ureidosuccinic acid)	C5H8N2O5	923-37-5	Organic acids (Amino acids)	Carbamoyl-Asp
2′-Deoxyadenosine 5′-monophosphate	C10H14N5O6P	653-63-4	Nucleotides	dAMP
2′-Deoxycytidine	C9H13N3O4	951-77-9	Nucleosides	dCdin
2-Deoxycytidine 5'-Monophosphate	C9H14N3O7P	1032-65-1	Nucleotides	dCMP
2'-Deoxyuridine	C9H12N2O5	951-78-0	Nucleosides	dUdin
2-Phosphoglycerate	C3H7O7P	2553-59-5	Carbohydrates and conjugates	2PG
3-Methyl-2-oxovaleric acid	C6H10O3	1460-34-0	Organic acids (Keto acids)	K-Ile
3-Methylcytidine	C10H15N3O5	2140-64-9	Nucleosides	Mcdin
3-Phosphoglycerate	C3H7O7P	820-11-1	Carbohydrates and conjugates	3PG
4-Hydroxy-proline	C5H9NO3	6912-67-0	Organic acids (Amino acids)	Hydroxyproline
5'-Deoxy-5'-Methylthioadenosine	C11H15N5O3S	2457-80-9	Nucleosides	5d5mtAsin
5-Methyluridine	C10H14N2O6	1463-10-1	Nucleosides	5MUdin
6-Phosphogluconate	C6H13O10P	921-62-0	Carbohydrates and conjugates	6PGA
Adenine	C5H5N5	73-24-5	Nucleobases	Ade
Adenosine	C10H13N5O4	58-61-7	Nucleosides	Asin
Adenosine 3′,5′-cyclic monophosphate	C10H12N5O6P	60-92-4	Nucleotides	cAMP
Adenosine 3'-monophosphate	C10H14N5O7P	84-21-9	Nucleotides	3AMP
Adenosine 5'-monophosphate	C10H14N5O7P	61-19-8	Nucleotides	5AMP
Adenosine 5′-triphosphate	C10H16N5O13P3	56-65-5	Nucleotides	ATP
Adenosine diphosphate	C10H15N5O10P2	58-64-0	Nucleotides	ADP
Alanine	C3H7NO2	302-72-7	Organic acids (Amino acids)	Ala
alpha-Aminoadipic acid	C6H11NO4	542-32-5	Organic acids (Amino acids)	AAA
alpha-Ketoglutarate	C5H6O5	328-50-7	Organic acids (Keto acids)	AKG
Arginine	C6H14N4O2	74-79-3	Organic acids (Amino acids)	Arg
Argininosuccinic acid	C10H18N4O6	2387-71-5	Organic acids (Amino acids)	Arg-Suc
Asparagine	C4H8N2O3	70-47-3	Organic acids (Amino acids)	Asn
Aspartic acid (Aspartate)	C4H7NO4	56-84-8	Organic acids (Amino acids)	Asp
Betaine	C5H11NO2	107-43-7	Organic acids (Amino acids)	Betaine
Biotin	C10H16N2O3S	58-85-5	Biotin and derivatives	Biotin
Carnitine	C7H15NO3	406-76-8	Quaternary ammonium salts	Carn

Compound	Molecular Formula	CAS number	Classification	Abbreviation
Choline chloride	C5H14NO	67-48-1	Quaternary ammonium salts	Choline
cis-Aconitate	C6H6O6	585-84-2	Organic acids (Carboxylic acids)	Aco
Citrate	C6H8O7	126-44-3	Organic acids (Carboxylic acids)	Cit
Cytidine-5'-monophosphate	C9H14N3O8P	63-37-6	Nucleotides	CMP
Cysteic acid	C3H7NO5S	13100-82-8	Organic acids (Amino acids)	Cysteic acid
Cysteine	C3H7NO2S	52-90-4	Organic acids (Amino acids)	Cys
Cysteinyl-glycine	C5H10N2O3S	19246-18-5	Organic acids (Amino acids)	Cys-Gly
Cystine	C6H12N2O4S2	923-32-0	Organic acids (Amino acids)	Cyst
Cytidine	C9H13N3O5	65-46-3	Nucleosides	Cdin
Cytidine 5′-triphosphate	C9H16N3O14P3	65-47-4	Nucleotides	CTP
Cytosine	C4H5N3O	71-30-7	Nucleobases	Cyt
Deoxyguanosine triphosphate	C10H16N5O13P3	2564-35-4	Nucleotides	dGTP
Dihydroxyacetonephosphate	C3H7O6P	57-04-5	Carbohydrates and conjugates	DHAP
Dihydroxyisovalerate	C5H10O4	19451-56-0	Fatty acyls	DHIV
Erythrito	C4H10O4	149-32-6	Carbohydrates and conjugates	EryOl
Erythrose-4-phosphate	C4H9O7P	585-18-2	Carbohydrates and conjugates	E4P
Flavinadenin dinucleotide	C27H33P2N9O15	146-14-5	Coenzymes and Vitamines	FAD
Fructose	C6H12O6	6347-01-9	Carbohydrates and conjugates	Fru
Fructose-1,6-bisphosphate	C6H14O12P2	34693-23-7	Carbohydrates and conjugates	FBP
Fructose-6-phosphate	C6H13O9P	643-13-0	Carbohydrates and conjugates	F6P
Fumarate	C4H4O4	142-42-7	Organic acids (Carboxylic acids)	Fum
Galactose	C6H12O6	10257-28-0	Carbohydrates and conjugates	Gal
Gluconate	C6H12O7	526-95-4	Carbohydrates and conjugates	Glc-ON
Glucose	C6H12O6	2280-44-6	Carbohydrates and conjugates	Glc
Glucose-1-phosphate	C6H13O9P	59-56-3	Carbohydrates and conjugates	G1P
Glucose-6-phosphate	C6H13O9P	299-31-0	Carbohydrates and conjugates	G6P
Glutamate	C5H9NO4	56-86-0	Organic acids (Amino acids)	Glu
Glutamine	C5H10N2O3	56-85-9	Organic acids (Amino acids)	Gln
Glutamyl-cysteine	C8H14N2O5S	686-58-8	Organic acids (Amino acids)	Glu-Cys
Glutathione, oxidized	C20H32N6O12S2	27025-41-8	Organic acids (Amino acids)	GSSG
Glutathione, reduced	C10H17N3O6S	70-18-8	Organic acids (Amino acids)	GSH
Glycine	C2H5NO2	56-40-6	Organic acids (Amino acids)	Gly
Glyoxylic acid	C2H2O3	298-12-4	Organic acids (Carboxylic acids)	Glyoxylate
Guanosine-5'-monophosphate	C10H14N5O8P	85-32-5	Nucleotides	GMP

Compound	Molecular Formula	CAS number	Classification	Abbreviation
Guanidineacetic acid	C3H7N3O2	352-97-6	Organic acids (Amino acids)	GdinAc
Guanine	C5H5N5O	73-40-5	Nucleobases	Gnin
Guanosine	C10H13N5O5	118-00-3	Nucleosides	Gsin
Guanosine 3′,5′-cyclic monophosphate	C10H12N5O7P	7665-99-8	Nucleotides	cGMP
Guanosine 5'-diphosphate	C10H15N5O11P2	146-91-8	Nucleotides	GDP
Guanosine 5′-triphosphate	C10H16N5O14P3	86-01-1	Guanosine 5′-triphosphate	GTP
Histidine	C6H9N3O2	71-00-1	Organic acids (Amino acids)	His
Homocysteine	C4H9NO2S	6027-13-0	Organic acids (Amino acids)	Hcys
Homoserine	C4H9NO3	1927-25-9	Organic acids (Amino acids)	H-Ser
Hydroxyglutaric acid	C5H8O5	2889-31-8	Organic acids (Hydroxy acids)	2HG
Inosine	C10H12N4O5	58-63-9	Nucleosides	Isin
Inosine 5'-monophosphate	C10H13N4O8P	131-99-7	Nucleotides	IMP
Inositol	C6H12O6	551-72-4	Carbohydrates and conjugates	Ino
Isocitrate	C6H8O7	320-77-4	Organic acids (Carboxylic acids)	I-Cit
Isoguanosine	C10H13N5O5	1818-71-9	Nucleosides	IGsin
Isoleucine	C6H13NO2	443-79-8	Organic acids (Amino acids)	Ile
Ketoisovalerate	C5H8O3	759-05-7	Organic acids (Keto acids)	K-Val
Kynurenine	C10H12N2O3	343-65-7	Organic acids (Amino acids)	Kynurenine
Lactate	C3H6O3	113-21-3	Organic acids (Hydroxy acids)	Lac
L-Citrulline	C6H13N3O3	372-75-8	Organic acids (Amino acids)	Citrulline
L-Cystathionine	C7H14N2O4S	56-88-2	Organic acids (Amino acids)	LCT
Leucine	C6H13NO2	61-90-5	Organic acids (Amino acids)	Leu
L-Ornithine	C5H12N2O2	70-26-8	Organic acids (Amino acids)	Orn
Lysine	C6H14N2O2	56-87-1	Organic acids (Amino acids)	Lys
Malic acid (Malate)	C4H6O5	320-77-4	Organic acids (Hydroxy acids)	Mali
Mannitol	C6H14O6	69-65-8	Carbohydrates and conjugates	Man-OL
Mannitol 1-phosphate	C6H15O9P	15806-48-1	Carbohydrates and conjugates	Man-OL-1-P
Mannose	C6H12O6	530-26-7	Carbohydrates and conjugates	Man
Melatonine	C13H16N2O2	73-31-4	Indoles and derivatives	Melatonine
Methionine	C5H11NO2S	63-68-3	Organic acids (Amino acids)	Met
Methionine sulfone	C5H11NO4S	7314-32-1	Organic acids (Amino acids)	MetSulf
Mevalonic acid	C6H12O4	17817-88-8	Fatty acyls	Meva
N4-Acetylcytidine	C11H15N3O6	3768-18-1	Nucleosides	AcCdin
N-Acetyl-Asp-Glu	C11H16N2O8	3106-85-2	Organic acids (Amino acids)	Acetyl-Asp-Glu
N-Acetyl-L-aspartic acid	C6H9NO5	997-55-7	Organic acids (Amino acids)	Acetyl-Asp

Compound	Molecular Formula	CAS number	Classification	Abbreviation
N-Acetyl-serine	C5H9NO4	16354-58-8	Organic acids (Amino acids)	N-Acetyl-DL-serine
NAD+	C21H27N7O14P2	53-84-9	Coenzymes and Vitamines	NAD
NADH	C21H29N7O14P2	58-68-4	Coenzymes and Vitamines	NADH
NADP+	C21H28N7O17P3	604-79-5	Coenzymes and Vitamines	NADP
NADPH	C21H30N7O17P3	53-57-6	Coenzymes and Vitamines	NADPH
Nicotinamide	C6H6N2O	98-92-0	Coenzymes and Vitamines	NAM
Octopamine	C8H11NO2	104-14-3	Benzenoids	Octopamine
Oxaloacetic acid	C4H4O5	328-42-7	Organic acids (Keto acids)	Oac
Palmitic acid	C16H32O2	57-10-3	Fatty acyls	Palm A
Phenylalanine	C9H11NO2	63-91-2	Organic acids (Amino acids)	Phe
Phosphocreatine	C4H10N3O5P	67-07-2	Organic acids (Amino acids)	P-Creatine
Proline	C5H9NO2	4305-67-3	Organic acids (Amino acids)	Pro
Propionyl-L-carnitine	C10H19NO4	17298-37-2	Fatty acyls	Prop. Carn
Pseudouridine	C9H12N2O6	1445-07-4	Nucleosides	Pudin
Pyruvate	C3H4O3	57-60-3	Organic acids (Keto acids)	Pyr
Ribose	C5H10O5	10257-32-6	Carbohydrates and conjugates	Rib
Ribose-5-phosphate	C5H11O8P	4300-28-1	Carbohydrates and conjugates	R5P
Ribulose-5-phosphate	C5H11O8P	551-85-9	Carbohydrates and conjugates	Rl5P
S-(Adenosyl)-methionine	C15H22N6O5S	29908-03-0	Coenzymes and Vitamines	SAM
Sarcosine	C3H7NO2	107-97-1	Organic acids (Amino acids)	Sarcosine
Sedoheptulose-7-phosphate	C7H15O10P	2646-35-7	Carbohydrates and conjugates	S7P
Seleno-methionine	C5H11NO2Se	3211-76-5	Organic acids (Amino acids)	Se-Met
Serine	C3H7NO3	56-45-1	Organic acids (Amino acids)	Ser
Serotonine	C10H12N2O	50-67-9	Indoles and derivatives	Serotonine
Spermidine	C7H19N3	124-20-9	Amines	Spermidine
Spermine	C10H26N4	71-44-3	Amines	Spermine
Succinate	C4H6O4	56-14-4	Organic acids (Carboxylic acids)	Suc
Thiamine hydrochloride	C12H17N4OS	67-03-8	Coenzymes and Vitamines	Thiamine
Threonine	C4H9NO3	72-19-5	Organic acids (Amino acids)	Thr
Thymidine	C10H14N2O5	50-89-5	Nucleosides	Tdin
Thymidine 5′-monophosphate	C10H15N2O8P	14057-65-9	Nucleotides	TMP
Thymidine 5'-triphosphate	C10H17N2O14P3	365-08-2	Nucleotides	TTP
Thymine	C5H6N2O2	65-71-4	Nucleobases	Thy
Trehalose	C12H22O11	99-20-7	Carbohydrates and conjugates	Tre
Tryptophan	C11H12N2O2	73-22-3	Organic acids (Amino acids)	Trp

Compound	Molecular Formula	CAS number	Classification	Abbreviation
Tyrosine	C9H11NO3	60-18-4	Organic acids (Amino acids)	Tyr
Uridine 5'-monophosphate	C9H13N2O9P	58-97-9	Nucleotides	UMP
Uracil	C4H4N2O2	66-22-8	Nucleobases	Ura
Uridine	C9H12N2O6	58-96-8	Nucleosides	Uri
Uridine 5′-diphosphate	C9H14N2O12P2	58-98-0	Nucleotides	UDP
Uridine 5′-triphosphate	C9H15N2O15P3	63-39-8	Nucleotides	UTP
Valine	C5H11NO2	72-18-4	Organic acids (Amino acids)	Val
Xanthine	C5H4N4O2	69-89-6	Nucleobases	Xan
Xylose	C5H10O5	10257-31-5	Carbohydrates and conjugates	Xyl
**Xenobiotics and human estrogens**				
16-Epiestriol	C18H24O3	547-81-9	Endogenous estrogens	16EpiE3
16-Hydroxyestrone	C18H22O3	566-76-7	Endogenous estrogens	16OHE1
17-Epiestriol	C18H24O3	1228-72-4	Endogenous estrogens	17EpiE3
1-OH-pyrene	C16H10O	5315-79-7	Air pollutant	1OHPy
2-Methoxyestradiol	C19H26O3	362-07-2	Endogenous estrogens	2MeOE2
2-tert Butylphenol	C10H14O	88-18-6	Industrial side product and pesticides	2-tert-BP
2-Hydroxyestradiol	C18H24O3	362-05-0	Endogenous estrogens	2OHE2
2-Methoxyestrone	C19H24O3	362-08-3	Endogenous estrogens	2MeOE1
2-Naphtol	C10H8O	135-19-3	Industrial side product and pesticides	2Naph
3-Benzylidencampher	C17H20O	15087-24-8	Personal care product ingredients and pharmaceuticals	3-BC
4-Methoxyestradiol	C19H26O3	26788-23-8	Endogenous estrogens	4MeOE2
4-Hydroxyestrone	C18H22O3	3131-23-5	Endogenous estrogens	4OHE1
4-Methoxyestrone	C19H24O3	58562-33-7	Endogenous estrogens	MeOE1
4-Methylbenzyliden campher	C18H22O	36861-47-9	Personal care product ingredients and pharmaceuticals	4-MBC
4-Octylphenol	C14H22O	1806-26-4	Industrial side product and pesticides	4-OP
4-tert-Octylphenol	C14H22O	140-66-9	Industrial side product and pesticides	4-tert-OP
8-Prenylnaringenin	C20H20O5	53846-50-7	Phytoestrogen	8-Pn
Aflatoxicol	C17H14O6	29611-03-8	Mycotoxin	AFL
Aflatoxin B1	C17H12O6	1162-65-8	Mycotoxin	AFB1
Aflatoxin B2	C17H14O6	7220-81-7	Mycotoxin	AFB2
Aflatoxin G1	C17H12O7	1165-39-5	Mycotoxin	AFG1
Aflatoxin G2	C17H14O7	7241-98-7	Mycotoxin	AFG2
Aflatoxin M1	C17H12O7	6795-23-9	Mycotoxin	AFM1
Aflatoxin M2	C17H14O7	6885-57-0	Mycotoxin	AFM2
Aflatoxin P1	C16H10O6	32215-02-4	Mycotoxin	AFP1
Aristolactam I	C17H11NO4	13395-02-3	Phytotoxin	Aristolactam
Alpha-zearalanol	C18H26O5	26538-44-3	Mycoestrogen	α-ZAL
Alpha-zearalenol	C18H24O5	36455-72-8	Mycoestrogen	α-ZEL
Alpha-zearalenol-14-glucuronide	C24H32O11	-	Mycoestrogen	α-ZEL-GlcA
Alternariol	C14H10O5	641-38-3	Mycoestrogen	Alternariol
Alternariol monomethyl ether	C15H12O5	26894-49-5	Mycoestrogen	AME
Anisodamine	C17H23NO4	55869-99-3	Phytotoxins	Anisodamine
Aristolochic acid I	C17H11NO7	313-67-7	Phytotoxins	AA
Beauvericin	C45H57N3O9	26048-05-5	Mycotoxin	BEA
Benzophenone 1	C13H10O3	131-56-6	Personal care product ingredients and pharmaceuticals	Benzophenone 1

Compound	Molecular Formula	CAS number	Classification	Abbreviation
Benzophenone 2	C13H10O5	131-55-5	Personal care product ingredients and pharmaceuticals	Benzophenone 2
Benzylbutyl phthalate	C19H20O4	85-68-7	Plasticizer	Benzyl butyl phthalate
Benzylparaben	C14H12O3	94-18-8	Personal care product ingredients and pharmaceuticals	B4HB
Beta-zearalanol	C18H26O5	42422-68-4	Mycoestrogen	β-ZAL
Beta-zearalenol	C18H24O5	71030-11-0	Mycoestrogen	β-ZEL
Beta-zearalenol-14-glucuronide	C24H32O11	-	Mycoestrogen	β-ZEL-GlcA
Bisphenol A	C15H16O2	80-05-07	Plasticizer	BPA
Bisphenol AF	C15H10F6O2	1478-61-1	Plasticizer	BPAF
Bisphenol B	C14H10Cl2O2	77-40-7	Plasticizer	BPB
Bisphenol C	C16H18O2	79-97-0	Plasticizer	BPC
Bisphenol F	C13H12O2	620-92-8	Plasticizer	BPF
Bisphenol S	C12H10O4S	80-09-1	Plasticizer	BPS
Butylparaben	C11H14O3	94-26-8	Personal care product ingredients and pharmaceuticals	Butylparaben
Citrinin	C13H14O5	518-75-2	Mycotoxin	CIT
Cotinine	C10H12N2O	486-56-6	Air pollutant	Cotinine
Coumestrol	C15H8O5	479-13-0	Phytoestrogen	Coumestrol
Daidzein	C15H10O4	486-66-8	Phytoestrogen	DAI
Deoxynivalenol	C15H20O6	51481-10-8	Mycotoxin	DON
Dibutylphthalate	C16H22O4	84-74-2	Plasticizer	Dibutylphthalate
Estradiol-17-glucuronide	C24H32O8	15087-02-2	Endogenous estrogens	E2-17-GlcA
Enterodiol	C18H22O4	80226-00-2	Phytoestrogen	Enterodiol
Enterolactone	C18H18O4	78473-71-9	Phytoestrogen	Enterolactone
Equol	C15H14O3	94105-90-5	Phytoestrogen	Equol
Estradiol	C18H24O2	50-28-2	Endogenous estrogens	E2
Estradiol-3-sulfate	C18H24O5S	4999-79-5	Endogenous estrogens	E2-3-sulfate
Estriol	C18H24O3	50-27-1	Endogenous estrogens	E3
Estrone	C18H22O2	53-16-7	Endogenous estrogens	E1
Ethinylestradiol	C20H24O2	57-63-6	Personal care product ingredients and pharmaceuticals	EE
Ethylparaben	C9H10O3	120-47-8	Personal care product ingredients and pharmaceuticals	Ethylparaben
Fenarimol	C17H12Cl2N2O	60168-88-9	Industrial side product and pesticides	Fenarimol
Formononetin	C16H12O4	485-72-3	Phytoestrogen	Formononetin
Genistein	C15H10O5	446-72-0	Phytoestrogen	GEN
Glycitein	C16H12O5	40957-83-3	Phytoestrogen	Glycitein
Isobutylparaben	C11H14O3	4247-02-3	Personal care product ingredients and pharmaceuticals	Isobutylparaben
Isoxanthohumol	C21H22O5	70872-29-6	Phytoestrogen	Isoxanthohumol
Jacobine	C18H25NO6	6870-67-3	Phytotoxins	Jacobine
Jacobine-N-oxide	C18H25NO7	38710-25-7	Phytotoxins	Jacobine-N-oxide
Matairesinol	C20H22O6	580-72-3	Phytoestrogen	Matairesinol
Mono-2-ethylhexyl phthalate	C16H21O4	4376-20-9	Plasticizer	MEHP
Methiocarb	C11H15NO2S	2032-65-7	Industrial side product and pesticides	Methiocarb
Methylparaben	C8H8O3	99-76-3	Personal care product ingredients and pharmaceuticals	Methylparaben
Monobutyl phthalate	C12H14O4	131-70-4	Plasticizer	MBP
n-Butylbenzolsulfonamid	C10H15NO2S	3622-84-2	Plasticizer	n-Butylbenzolsulfonamid

Compound	Molecular Formula	CAS number	Classification	Abbreviation
Nivalenol	C15H20O7	23282-20-4	Mycotoxin	NIV
Nonylphenol	C15H24O	84852-15-3	Mycotoxin	Nonylphenol
Ochratoxin A	C20ClH18NO6	303-47-9	Mycotoxin	OTA
Ochratoxin Alpha	C11H10O5	19165-63-0	Mycotoxin	Otα
Ochratoxin B	C11ClH9O5	4825-86-9	Mycotoxin	OTB
Octyl methoxycinnamate	C18H26O3	5466-77-3	Personal care product ingredients and pharmaceuticals	OMC
Perfluoroctanoic acid	C8HF15O2	335-67-1	Perfluorinated alkylated substances	PFOA
Perfluorooctanesulfonic acid	C8HF17O3S	1763-23-1	Perfluorinated alkylated substances	PFOS
PhIP	C13H12N4	105650-23-5	Food processing by-products	PhIP
p-Hydrobenzoic acid	C7H6O3	99-96-7	Personal care product ingredients and pharmaceuticals	pOHBA
Prochloraz	C15H16Cl3N3O2	67747-09-5	Industrial side product and pesticides	Prochloraz
Propylparaben	C10H12O3	94-13-3	Personal care product ingredients and pharmaceuticals	Propylparaben
Resveratrol	C14H12O3	501-36-0	Phytoestrogen	Resveratrol
Riddeliin	C18H23NO6	23246-96-0	Phytotoxins	Riddeliin
Riddeliin-N-oxide	C18H23NO7	75056-11-0	Phytotoxins	Riddeliin-N-oxide
Scopolamine	C17H21NO4	51-34-3	Phytotoxins	Scopolamine
Sterigmatocystein	C18H12O6	10048-13-2	Mycotoxin	Sterigmatocystein
Tentoxin	C22H30N4O4	28540-82-1	Mycotoxin	Tentoxin
Tetrabrombisphenol A	Br4C15H12O2	79-94-7	Plasticizer	TBPA
HT-2 Toxin	C22H32O8	26934-87-2	Mycotoxin	HT2
T-2 Toxin	C24H34O9	21259-20-1	Mycotoxin	T2
Trans-3-hydroxy-cotinine	C10H12N2O2	34834-67-8	Air pollutant	trans-3-OH-cotinine
Triclosan	C12H7Cl3O2	3380-34-5	Industrial side product and pesticides	Triclosan
Xanthohumol	C21H22O5	569-83-5	Phytoestrogen	Xanthohumol
Zearalanone	C18H22O5	5975-78-0	Mycoestrogen	ZAN
Zearalenone	C18H24O5	17924-92-4	Mycoestrogen	ZEN
Zearalenone-14-glucuronide	C24H30O11	-	Mycoestrogen	ZEN-14-GlcA
Zearalenone-14-sulfate	C18H22O8S	-	Mycoestrogen	ZEN-14-sulfate
		**Internal standards**		
$^{13}C_{12}$-Bisphenol A		263261-85-0		
$^{13}C_{18}$- Zearalenone				
$^{13}C_{4}$-Monobutyl phthalate				
$^{13}C_{4}$-MEHP				
$^{13}C_{3}$-Estradiol		1261254-48-1		
$^{13}C_{6}$-4-tert-octylphenol		1173020-24-0		
$^{13}C_{6}$-Butylparaben				
$^{13}C_{6}$-Ethylparaben				
$^{13}C_{6}$-Methylparaben				
$^{13}C_{6}$-p-Hydroxybenzoic acid		267399-29-5		
$^{13}C_{6}$-Propylparaben				
$^{13}C_{8}$-PFOA				
$^{13}C_{8}$-PFOS				
$^{13}C_{15}$-Deoxynivalenol				
$^{13}C_{17}$-Aflatoxin M1				

Table S2 Analyte concentrations in the solvent multi-analyte standard. The same concentration levels were used for the preparation of the corresponding matrix-matched standard.

**Compound**	**Concentration [ng/mL]**							
	**Std 1**	**Std 2**	**Std 3**	**Std 4**	**Std 5**	**Std 6**	**Std 7**	**Std 8**
	**Endogenous metabolites excluding human estrogens**							
	**0.001 µM**	**0.01 µM**	**0.03 µM**	**0.1 µM**	**0.3 µM**	**1 µM**	**3 µM**	**10 µM**
1-Methylhydantoin	0.1	1.1	3.4	11.4	34.2	114.1	342.3	1141
1-Methylnicotinamide	0.1	1.4	4.1	13.7	41.1	137.2	411.5	1371.6
2-(Carbamoylamino)butanedioic acid	0.2	1.8	5.3	17.6	52.8	176.1	528.4	1761.3
2'-Deoxyadenosine 5'-monophosphate	0.3	3.3	9.9	33.1	99.4	331.2	993.7	3312.2
2'-Deoxycytidine	0.2	2.3	6.8	22.7	68.2	227.2	681.7	2272.2
2-Deoxycytidine 5'-Monophosphate	0.3	3.1	9.2	30.7	92.2	307.2	921.6	3072
2'-Deoxyuridine	0.2	2.3	6.8	22.8	68.5	228.2	684.6	2282
2-Phosphoglyceric acid	0.2	1.9	5.6	18.6	55.8	186.1	558.2	1860.6
3'AMP	0.3	3.5	10.4	34.7	104.2	347.2	1041.7	3472.2
3-Methyl-2-oxovaleric acid	0.1	1.3	3.9	13	39.0	130.1	390.4	1301.39
3-Methylcytidine	0.3	2.6	7.7	25.7	77.2	257.2	771.7	2572.4
3-Phosphoglycerate	0.2	1.9	5.6	18.6	55.8	186.1	558.2	1860.6
4-Hydroxy-proline	0.1	1.3	3.9	13.1	39.3	131.1	393.4	1311.3
5'-Deoxy-5'-Methylthioadenosine	0.3	3	8.9	29.7	89.2	297.3	892.0	2973.4
5-Methyluridine	0.3	2.6	7.7	25.8	77.5	258.2	774.7	2582.3
6-Phosphogluconate	0.3	2.8	8.3	27.6	82.8	276.1	828.4	2761.4
Adenine	0.1	1.4	4.1	13.5	40.5	135.1	405.4	1351.3
Adenosine	0.3	2.7	8	26.7	80.2	267.2	801.7	2672.4
Adenosine 3',5'-cyclic monophosphate	0.3	3.3	9.9	32.9	98.8	329.2	987.6	3292.1
Adenosine 5'-triphosphate	0.5	5.1	15.2	50.7	152.2	507.2	1521.5	5071.8
Adenosine diphosphate	0.4	4.3	12.8	42.7	128.2	427.2	1281.6	4272
Alanine	0.1	0.9	2.7	8.9	26.7	89.1	267.3	890.9
alpha-Aminoadipic acid	0.2	1.6	4.8	16.1	48.3	161.2	483.5	1611.6
alpha-Ketoglutarate	0.1	1.5	4.4	14.6	43.8	146.1	438.3	1461
5'AMP	0.3	3.5	10.4	34.7	104.2	347.2	1041.7	3472.2
Arginine	0.2	1.7	5.2	17.4	52.3	174.2	522.6	1742
Argininosuccinic acid	0.3	2.9	8.7	29	87.1	290.3	870.8	2902.7
Asparagine	0.1	1.3	4	13.2	39.6	132.1	396.4	1321.2
Aspartate	0.1	1.3	4	13.3	39.9	133.1	399.3	1331
Betaine	0.1	1.2	3.5	11.7	35.1	117.2	351.5	1171.5
Biotin	0.2	2.4	7.3	24.4	73.3	244.3	732.9	2443.1
Carnitine	0.2	1.6	4.8	16.1	48.4	161.2	483.6	1612
Choline chloride	0.1	1	3.1	10.4	31.3	104.2	312.5	1041.7
cis-Aconitate	0.2	1.7	5.2	17.4	52.2	174.1	522.3	1741.1
Citrate	0.2	1.9	5.8	19.2	57.6	192.1	576.4	1921.2
CMP	0.3	3.2	9.7	32.3	97.0	323.2	969.6	3232
Cysteic acid	0.2	1.7	5.1	16.9	50.7	169.2	507.5	1691.6
Cysteine	0.1	1.2	3.6	12.1	36.3	121.2	363.5	1211.6
Cysteinyl-glycine	0.2	1.8	5.3	17.8	53.5	178.2	534.6	1782.1
Cystine	0.2	2.4	7.2	24	72.1	240.3	720.9	2403
Cytidine	0.2	2.4	7.3	24.3	73.0	243.2	729.7	2432.2
Cytidine 5'-triphosphate	0.5	4.8	14.5	48.3	144.9	483.2	1449.5	4831.6
Cytosine	0.1	1.1	3.3	11.1	33.3	111.1	333.3	1111
Deoxyguanosine triphosphate	0.5	5.1	15.2	50.7	152.2	507.2	1521.5	5071.8
Dihydroxyacetonephosphate	0.2	1.7	5.1	17	51.0	170.1	510.2	1700.6
Dihydroxyisovalerate	0.1	1.3	4	13.4	40.2	134.1	402.4	1341.3
Erythrito	0.1	1.2	3.7	12.2	36.6	122.1	366.4	1221.2
Erythrose-4-phosphate	0.2	2	6	20	60.0	200.1	600.2	2000.8

Compound	Concentration [ng/mL]							
	Std 1	Std 2	Std 3	Std 4	Std 5	Std 6	Std 7	Std 8
Flavinadenin dinucleotide	0.8	7.9	23.6	78.6	235.7	785.5	2356.5	7855
Fructose	0.2	1.8	5.4	18	54.0	180.2	540.4	1801.6
Fructose-1,6-bisphosphate	0.3	3.4	10.2	34	102.0	340.1	1020.4	3401.2
Fructose-6-phosphate	0.3	2.6	7.8	26	78.0	260.1	780.4	2601.4
Fumarate	0.1	1.2	3.5	11.6	34.8	116.1	348.2	1160.7
Galactose	0.2	1.8	5.4	18.0	54.0	180.2	540.5	1801.6
Gluconate	0.2	2	5.9	19.6	58.8	196.2	588.5	1961.6
Glucose	0.2	1.8	5.4	18.0	54.0	180.16	540.5	1801.6
Glucose-1-phosphate	0.3	2.6	7.8	26	78.0	260.1	780.4	2601.4
Glucose-6-phosphate	0.3	2.6	7.8	26	78.0	260.1	780.4	2601.4
Glutamate	0.1	1.5	4.4	14.7	44.1	147.1	441.4	1471.3
Glutamine	0.1	1.5	4.4	14.6	43.8	146.1	438.4	1461.4
Glutamyl-cysteine	0.3	2.5	7.5	25	75.1	250.3	750.8	2502.7
Glutathione, oxidized	0.6	6.1	18.4	61.3	183.8	612.6	1837.8	6126
Glutathione, reduced	0.3	3.1	9.2	30.7	92.2	307.3	922.0	3073.3
Glycine	0.1	0.8	2.3	7.5	22.5	75.1	225.2	750.7
Glyoxylic acid	0.1	0.7	2.2	7.4	22.2	74.0	222.1	740.4
GMP	0.4	3.6	10.9	36.3	109.0	363.2	1089.7	3632.2
Guanidineacetic acid	0.1	1.2	3.5	11.7	35.1	117.1	351.3	1171.1
Guanine	0.2	1.5	4.5	15.1	45.3	151.1	453.4	1511.3
Guanosine	0.3	2.8	8.5	28.3	85.0	283.2	849.7	2832.4
Guanosine 3',5'-cyclic monophosphate	0.3	3.5	10.4	34.5	103.6	345.2	1035.6	3452.1
Guanosine 5'-diphosphate	0.4	4.4	13.3	44.3	133.0	443.2	1329.6	4432
Guanosine 5'-triphosphate	0.5	5.2	15.7	52.3	157.0	523.2	1569.5	5231.8
Histidine	0.2	1.6	4.7	15.5	46.5	155.2	465.5	1551.5
Homocysteine	0.1	1.4	4.1	13.5	40.6	135.2	405.6	1351.9
Homoserine	0.1	1.2	3.6	11.9	35.7	119.1	357.4	1191.2
Hydroxyglutaric acid	0.1	1.5	4.4	14.8	44.4	148.1	444.3	1481.1
Inosine	0.3	2.7	8	26.8	80.5	268.2	804.7	2682.3
Inosine 5'-monophosphate	0.3	3.5	10.4	34.8	104.5	348.2	1044.6	3482.1
Inositol	0.2	1.8	5.4	18.0	54.0	180.2	540.5	1801.6
Isocitrate	0.2	1.9	5.8	19.2	57.6	192.1	576.4	1921.2
Isoguanosine	0.3	2.8	8.5	28.3	85.0	283.2	849.7	2832.4
Isoleucine	0.1	1.3	3.9	13.1	39.4	131.2	393.5	1311.7
Ketoisovalerate	0.3	2.8	8.3	27.5	82.6	275.5	826.5	2754.9
Kynurenine	0.2	2.1	6.2	20.8	62.5	208.2	624.6	2082.1
Lactate	0.1	0.9	2.7	9	27.0	90.1	270.2	900.8
L-Citrulline	0.2	1.8	5.3	17.5	52.6	175.2	525.6	1751.9
L-Cystathionine	0.2	2.2	6.7	22.2	66.7	222.3	666.8	2222.6
Leucine	0.1	1.3	3.9	13.1	39.4	131.2	393.5	1311.7
L-Ornithine	0.1	1.3	4	13.2	39.6	132.2	396.5	1321.6
Lysine	0.1	1.5	4.4	14.6	43.9	146.2	438.6	1461.9
Malate	0.1	1.3	4	13.4	40.2	134.1	402.3	1340.9
Mannitol	0.2	1.8	5.5	18.2	54.7	182.2	546.5	1821.7
Mannitol 1-phosphate	0.3	2.6	7.9	26.2	78.6	262.2	786.5	2621.5
Mannose	0.2	1.8	5.4	18.0	54.0	180.2	540.5	1801.6
Melatonine	0.2	2.3	7	23.2	69.7	232.3	696.8	2322.8
Methionine	0.1	1.5	4.5	14.9	44.8	149.2	447.6	1492.1
Methionine sulfone	0.2	1.8	5.4	18.1	54.4	181.2	543.6	1812.1
Mevalonic acid	0.1	1.5	4.4	14.8	44.4	148.2	444.5	1481.6
N4-Acetylcytidine	0.3	2.9	8.6	28.5	85.6	285.3	855.8	2852.5
N-Acetyl-Asp-Glu	0.3	3	9.1	30.4	91.3	304.3	912.8	3042.5
N-Acetyl-L-aspartic acid	0.2	1.8	5.3	17.5	52.5	175.1	525.4	1751.4
N-Acetyl-serine	0.1	1.5	4.4	14.7	44.1	147.1	441.4	1471.3
NAD+	0.7	6.6	19.9	66.3	199.0	663.4	1990.2	6634
NADH	0.7	6.7	20	66.5	199.6	665.4	1996.2	6654
NADP+	0.7	7.4	22.3	74.4	223.3	744.4	2233.2	7444

Compound	Concentration [ng/mL]							
	Std 1	Std 2	Std 3	Std 4	Std 5	Std 6	Std 7	Std 8
NADPH	0.7	7.5	22.4	74.5	223.6	745.4	2236.2	7454
Nicotinamide	0.1	1.2	3.7	12.2	36.6	122.1	366.4	1221.2
Octopamine	0.2	1.5	4.6	15.3	46.0	153.2	459.5	1531.8
Oxaloacetic acid	0.1	1.3	4	13.2	39.6	132.1	396.2	1320.7
Palmitic acid	0.3	2.6	7.7	25.6	76.9	256.4	769.3	2564.2
Phenylalanine	0.2	1.7	5	16.5	49.6	165.2	495.6	1651.9
Phosphocreatine	0.2	2.1	6.3	21.1	63.3	211.1	633.3	2111.1
Proline	0.1	1.2	3.5	11.5	34.5	115.1	345.4	1151.3
Propionyl-L-carnitine	0.2	2.2	6.5	21.7	65.2	217.3	651.8	2172.6
Pseudouridine	0.2	2.4	7.3	24.4	73.3	244.2	732.6	2442
Pyruvate	0.1	0.9	2.6	8.8	26.4	88.1	264.2	880.6
Ribose	0.2	1.5	4.5	15	45.0	150.1	450.4	1501.3
Ribose-5-phosphate	0.2	2.3	6.9	23	69.0	230.1	690.3	2301.1
Ribulose-5-phosphate	0.2	2.3	6.9	23	69.0	230.1	690.3	2301.1
S-(Adenosyl)-methionine	0.4	3.8	11.5	38.4	115.3	384.4	1153.2	3844.1
Sarcosine	0.1	0.9	2.7	8.9	26.7	89.1	267.3	890.9
Sedoheptulose-7-phosphate	0.3	2.9	8.7	29	87.0	290.2	870.5	2901.6
Seleno-methionine	0.2	2	5.9	19.6	58.8	196.1	588.4	1961.2
Serine	0.1	1.1	3.2	10.5	31.5	105.1	315.3	1050.9
Serotonine	0.2	1.8	5.3	17.6	52.9	176.2	528.6	1762.1
Spermidine	0.1	1.5	4.4	14.5	43.6	145.3	435.8	1452.5
Spermine	0.2	2	6.1	20.2	60.7	202.3	607.0	2023.4
Succinate	0.1	1.2	3.5	11.8	35.4	118.1	354.3	1180.9
Thiamine hydrochloride	0.3	2.7	8	26.5	79.6	265.4	796.1	2653.6
Threonine	0.1	1.2	3.6	11.9	35.7	119.1	357.4	1191.2
Thymidine	0.2	2.4	7.3	24.2	72.7	242.2	726.7	2422.3
Thymidine 5'-monophosphate	0.3	3.2	9.6	32.1	96.4	321.2	963.6	3212
Thymine	0.1	1.3	3.8	12.6	37.8	126.1	378.3	1261.1
Trehalose	0.3	3.4	10.3	34.2	102.7	342.3	1026.9	3423
Tryptophan	0.01	0.1	0.3	1	3.0	10.0	30.0	100
TTP (Thymidinetriphosphate)	0.2	2	6.1	20.4	61.3	204.2	612.7	2042.2
Tyrosine	0.5	4.8	14.5	48.2	144.7	482.2	1446.5	4821.7
UMP	0.3	3.2	9.7	32.4	97.3	324.2	972.5	3241.8
Uracil	0.1	1.1	3.4	11.2	33.6	112.1	336.3	1120.9
Uridine	0.2	2.4	7.3	24.4	73.3	244.2	732.6	2442
Uridine 5'-diphosphate	0.4	4	12.1	40.4	121.2	404.2	1212.5	4041.6
Uridine 5'-triphosphate	0.5	4.8	14.5	48.4	145.2	484.1	1452.4	4841.4
Valine	0.1	1.2	3.5	11.7	35.1	117.2	351.5	1171.5
Xanthine	0.2	1.5	4.6	15.2	45.6	152.1	456.3	1521.1
Xylose	0.2	1.5	4.5	15	45.0	150.1	450.4	1501.3
	**Xenobiotics and human estrogens**							
16-Epiestriol	0.05	0.5	1.5	5	15.0	50.0	150.0	500
16-Hydroxyestrone	0.01	0.1	0.3	1	3.0	10.0	30.0	100
17-Epiestriol	0.05	0.5	1.5	5	15.0	50.0	150.0	500
1-OH-pyrene	0.01	0.1	0.3	1	3.0	10.0	30.0	100
2 Methoxyestradiol	0.03	0.3	0.9	3	9.0	30.0	90.0	300
2 tert Butylphenol	0.2	2	6	20	60.0	200.0	600.0	2000
2-Hydroxyestradiol	0.1	1	3	10	30.0	100.0	300.0	1000
2-Methoxyestrone	0.03	0.3	0.9	3	9.0	30.0	90.0	300
2-Naphtol	0.05	0.5	1.5	5	15.0	50.0	150.0	500
3-Benzylidencampher	0.5	5	15	50	150.0	500.0	1500.0	5000
4 Methoxyestradiol	0.01	0.1	0.2	0.5	1.5	5.0	15.0	50
4-Hydroxyestrone	0.05	0.5	1.5	5	15.0	50.0	150.0	500
4-Methoxyestrone	0.01	0.1	0.2	0.5	1.5	5.0	15.0	50
4-Methylbenzyliden campher	0.2	2	6	20	60.0	200.0	600.0	2000
4-octylphenol	0.1	1	3	10	30.0	100.0	300.0	1000
4-tert-octylphenol	0.1	1	3	10	30.0	100.0	300.0	1000
8-Prenylnaringenin	0.001	0.01	0.03	0.1	0.3	1.0	3.0	10

Compound	Concentration [ng/mL]							
	Std 1	Std 2	Std 3	Std 4	Std 5	Std 6	Std 7	Std 8
Aflatoxicol	0.01	0.1	0.2	0.5	1.5	5.0	15.0	50
Aflatoxin B1	0.01	0.1	0.3	1	3.0	10.0	30.0	100
Aflatoxin B2	0.01	0.1	0.3	1	3.0	10.0	30.0	100
Aflatoxin G1	0.01	0.1	0.3	1	3.0	10.0	30.0	100
Aflatoxin G2	0.01	0.1	0.3	1	3.0	10.0	30.1	100.3
Aflatoxin M1	0.01	0.1	0.3	1	3.0	10.0	30.0	100
Aflatoxin M2	0.01	0.1	0.3	1	3.0	10.0	30.1	100.3
Aflatoxin P1	0.01	0.1	0.3	1	3.0	10.0	30.0	100
Aristolactam I	0.01	0.1	0.3	1	3.0	10.0	30.0	100
Alpha-zearalanol	0.01	0.1	0.3	1	3.0	10.0	30.0	100
Alpha-zearalenol	0.01	0.1	0.3	1	3.0	10.0	30.0	100
Alpha-zearalenol-14-glucuronide	0.01	0.1	0.3	1	3.0	10.0	30.0	100
Alternariol	0.01	0.1	0.3	1	3.0	10.0	30.0	100
Alternariol monomethyl ether	0.01	0.1	0.3	1	3.0	10.0	30.0	100
Anisodamine	0.01	0.1	0.3	1	3.0	10.0	30.0	100
Aristolochic acid I	0.01	0.1	0.3	1	3.0	10.0	30.0	100
Beauvericin	0.01	0.1	0.2	0.5	1.5	5.0	15.0	50
Benzophenone 1	0.001	0.01	0.03	0.1	0.3	1.0	3.0	10
Benzophenone 2	0.01	0.1	0.2	0.5	1.5	5.0	15.0	50
Benzylbutyl phthalate	0.05	0.5	1.5	5	15.0	50.0	150.0	500
Benzylparaben (B4HB)	0.001	0.01	0.02	0.1	0.2	0.5	1.5	5
Beta-zearalanol	0.01	0.1	0.3	1	3.0	10.0	30.0	100
Beta-zearalenol	0.01	0.1	0.3	1	3.0	10.0	30.0	100
Beta-zearalenol-14-glucuronide	0.01	0.1	0.3	1	3.0	10.0	30.0	100
Bisphenol A	0.01	0.1	0.3	1	3.0	10.0	30.0	100
Bisphenol AF	0.01	0.1	0.2	0.5	1.5	5.0	15.0	50
Bisphenol B	0.01	0.1	0.3	1	3.0	10.0	30.0	100
Bisphenol C	0.02	0.2	0.6	2	6.0	20.0	60.0	200
Bisphenol F	0.02	0.2	0.6	2	6.0	20.0	60.0	200
Bisphenol S	0.001	0.01	0.03	0.1	0.3	1.0	3.0	10
Butylparaben	0.01	0.1	0.2	0.5	1.5	5.0	15.0	50
Citrinin	0.01	0.1	0.3	1	3.0	10.0	30.0	100
Cotinine	0.01	0.1	0.3	1	3.0	10.0	30.0	100
Coumestrol	0.01	0.1	0.3	1	3.0	10.0	30.0	100
Daidzein	0.01	0.1	0.2	0.5	1.5	5.0	15.0	50
Deoxynivalenol	0.01	0.1	0.3	1	3.0	10.0	30.0	100
Dibutylphthalate	0.1	1	3	10	30.0	100.0	300.0	1000
E2-17-GlcA	0.05	0.5	1.5	5	15.0	50.0	150.0	500
Enterodiol	0.01	0.1	0.2	0.5	1.5	5.0	15.0	50
Enterolactone	0.01	0.1	0.3	1	3.0	10.0	30.0	100
Equol	0.01	0.1	0.3	1	3.0	10.0	30.0	100
Estradiol	0.02	0.2	0.6	2	6.0	20.0	60.0	200
Estradiol-3-sulfate	0.02	0.2	0.6	2	6.0	20.0	60.0	200
Estriol	0.02	0.2	0.6	2	6.0	20.0	60.0	200
Estrone	0.01	0.1	0.3	1	3.0	10.0	30.0	100
Ethinylestradiol	0.05	0.5	1.5	5	15.0	50.0	150.0	500
Ethylparaben	0.01	0.1	0.2	0.5	1.5	5.0	15.0	50
Fenarimol	0	0	0	0.1	0.2	0.5	1.5	5
Formononetin	0.01	0.1	0.2	0.5	1.5	5.0	15.0	50
Genistein	0.01	0.1	0.2	0.5	1.5	5.0	15.0	50
Glycitein	0.01	0.1	0.2	0.5	1.5	5.0	15.0	50
Isobutylparaben	0.01	0.1	0.2	0.5	1.5	5.0	15.0	50
Isoxanthohumol	0	0	0	0.1	0.2	0.5	1.5	5
Jacobine	0.01	0.1	0.3	1	3.0	10.0	30.0	100
Jacobine-N-oxide	0.01	0.1	0.3	1	3.0	10.0	30.0	100
Matairesinol	0.02	0.2	0.6	2	6.0	20.0	60.0	200
MEHP	0.02	0.2	0.6	2	6.0	20.0	60.0	200
Methiocarb	0.03	0.3	0.9	3	9.0	30.0	90.0	300

Compound	Concentration [ng/mL]							
	Std 1	Std 2	Std 3	Std 4	Std 5	Std 6	Std 7	Std 8
Methylparaben	0.01	0.1	0.3	1	3.0	10.0	30.0	100
Monobutyl phthalate	0.02	0.2	0.6	2	6.0	20.0	60.0	200
n Butylbenzolsulfonamid	0.02	0.2	0.6	2	6.0	20.0	60.0	200
Nivalenol	0.01	0.1	0.3	1	3.0	10.0	30.0	100
Nonylphenol	0.05	0.5	1.5	5	15.0	50.0	150.0	500
Ochratoxin A	0.01	0.1	0.3	1	3.0	10.0	30.0	100
Ochratoxin Alpha	0.01	0.1	0.3	1	3.0	10.0	30.0	100
Ochratoxin B	0.01	0.1	0.3	1	3.0	10.0	30.0	100
Octyl methoxycinnamate	0.5	5	15	50	150.0	500.0	1500.0	5000
Perfluoroctanoic acid (PFOA)	0.01	0.1	0.2	0.5	1.5	5.0	15.0	50
PFOS	0.03	0.3	0.9	3	9.0	30.0	90.0	300
PhIP	0.01	0.1	0.3	1	3.0	10.0	30.0	100
p-Hydrobenzoic acid	0.3	3	9	30	90.0	300.0	900.0	3000
Prochloraz	0	0	0	0.1	0.3	1.0	3.0	10
Propylparaben	0.01	0.1	0.2	0.5	1.5	5.0	15.0	50
Resveratrol	0.05	0.5	1.5	5	15.0	50.0	150.0	500
Riddeliin	0.08	0.8	2.4	8	24.0	80.0	240.0	800
Riddeliin-N-oxide	0.08	0.8	2.4	8	24.0	80.0	240.0	800
Scopolamine	0.01	0.1	0.2	0.5	1.5	5.0	15.0	50
Sterigmatocystein	0.01	0.1	0.3	1	3.0	10.0	30.0	100
Tentoxin	0.01	0.1	0.3	1	3.0	10.0	30.0	100
Tetrabrombisphenol A	0.02	0.2	0.6	2	6.0	20.0	60.0	200
Toxin HT 2	0.01	0.1	0.3	1	3.0	10.0	30.0	100
Toxin T2	0.01	0.1	0.3	1	3.0	10.0	30.0	100
trans-3-OH-cotinine	0.08	0.8	2.4	8	24.0	80.0	240.0	800
Triclosan	0.01	0.1	0.3	1	3.0	10.0	30.0	100
Xanthohumol	0.01	0.1	0.2	0.5	1.5	5.0	15.0	50
Zearalanone	0.01	0.1	0.3	1	3.0	10.0	30.0	100
Zearalenone	0.01	0.1	0.3	1	3.0	10.0	30.0	100
Zearalenone-14-glucuronide	0.01	0.1	0.3	1	3.0	10.0	30.0	100
Zearalenone-14-sulfate	0.01	0.1	0.3	1	3.0	10.0	30.0	100

Table S3 Concentrations of ^{13}C-labeled analytes in the internal standard mix

Compound	Concentration [ng/mL]
$^{13}C_{12}$-Bisphenol A	100
$^{13}C_{18}$- Zearalenone	200
$^{13}C_{4}$-Monobutyl phthalate	800
$^{13}C_{4}$-MEHP	800
$^{13}C_{3}$-Estradiol	400
$^{13}C_{6}$-4-tert-octylphenol	500
$^{13}C_{6}$-Butylparaben	100
$^{13}C_{6}$-Ethylparaben	100
$^{13}C_{6}$-Methylparaben	100
$^{13}C_{6}$-p-Hydroxybenzoic acid	8000
$^{13}C_{6}$-Propylparaben	100
$^{13}C_{8}$-PFOA	100
$^{13}C_{8}$-PFOS	100
$^{13}C_{15}$-Deoxynivalenol	400
$^{13}C_{17}$-Aflatoxin M1	20

Table S4 Average peak area of individual endogenous metabolites (analysed with the HILIC column) and xenobiotics/endogenous estrogen metabolites (analysed with the RP column) measured with different eluent/column combinations. The average peak area over all analytes was calculated for each combination for the selection of the best column/eluent combination. The highest value is highlighted in bold.

	Average Area							
	Comb.1	Comb.2	Comb.3	Comb.5	Comb.1	Comb.2	Comb.3	Comb.5
	Solvent				Urine			
**Endogenous metabolites excluding endogenous human estrogens (HILIC)**								
Alanine	**2.59E+08** ±2.63E+06	2.16E+08±6.87E+06	1.54E+08±1.32E+07	1.10E+08±6.73E+06	**6.34E+09**±7.10E+07	4.33E+09±2.34E+08	3.78E+09±1.76E+08	2.30E+09±3.80E+07
Glutamine	3.65E+08 ±1.39E+07	4.22E+08±2.24E+07	1.38E+08±3.00E+06	**6.33E+08**±2.03E+07	4.16E+09±1.65E+07	**6.42E+09**±1.16E+08	4.79E+09±3.54E+08	1.56E+09±4.00E+07
Isoleucine	1.73E+08 ±2.43E+06	**4.47E+08**±1.61E+07	3.60E+08±2.46E+07	1.21E+08±2.51E+06	**1.26E+09**±1.72E+07	9.00E+08±1.34E+08	1.17E+09±2.54E+08	1.18E+09±2.85E+07
Phenylalanine	**3.81E+08** ±3.88E+06	3.41E+08±9.99E+06	2.40E+08±5.51E+06	2.70E+08±7.88E+06	9.70E+08±4.62E+07	1.27E+09±1.18E+08	**1.91E+09**±1.37E+08	1.76E+09±3.83E+07
Glutathione reduced	2.38E+05 ±1.29E+05	**1.89E+06**±3.98E+05	5.89E+05±1.78E+05	3.46E+05±2.44E+05	1.12E+07±1.57E+06	**1.26E+07**±1.87E+06	7.69E+06±1.80E+06	5.26E+06±9.79E+05
Methionine sulfone	1.37E+08 ±8.47E+05	**1.61E+08**±4.97E+06	1.06E+08±8.19E+06	1.00E+08±3.72E+06	1.71E+08±9.14E+06	1.71E+08±1.25E+07	**1.93E+08**±4.88E+06	3.66E+07±1.94E+06
N-Acetyl-serine	**3.96E+08** ±4.40E+06	2.80E+08±2.06E+07	2.70E+08±1.52E+07	9.45E+07±1.03E+07	**1.41E+09**±2.70E+07	7.23E+08±3.09E+07	1.13E+08±2.55E+07	4.31E+08±1.86E+07
Glutamate	4.81E+07 ±1.13E+06	**5.51E+07**±4.34E+06	3.95E+07±3.60E+06	1.65E+07±1.12E+06	1.48E+08±6.93E+06	**1.83E+08**±2.29E+07	1.58E+08±1.52E+07	5.73E+07±2.25E+06
Carnitine	1.28E+10 ±3.42E+08	1.31E+10±5.74E+08	**1.41E+10**±9.01E+08	1.11E+10±5.70E+08	8.48E+10±7.49E+08	9.68E+10±7.53E+09	**1.02E+11**±8.57E+09	2.08E+10±2.38E+08
NAD+	5.72E+07 ±4.74E+06	**9.38E+07**±2.98E+06	7.07E+07±5.24E+06	4.14E+07±2.01E+06	1.84E+07±1.59E+07	2.67E+07±4.67E+06	2.15E+07±2.92E+06	**2.92E+07**±2.34E+06
Pseudouridine	**3.23E+08** ±5.31E+06	2.64E+08±1.16E+07	1.82E+08±9.21E+06	5.96E+07±6.79E+06	4.86E+09±3.62E+07	**5.43E+09**±6.14E+08	4.47E+09±5.91E+08	2.12E+09±2.91E+07
2-dAMP	**4.77E+08** ±1.87E+07	2.25E+08±1.69E+07	1.24E+08±1.76E+07	2.09E+08±1.03E+07	**1.15E+08**±7.72E+06	9.32E+07±6.13E+06	3.73E+07±7.35E+06	4.20E+07±2.30E+06
alpha-Ketoglutarate	**1.20E+08** ±1.71E+06	8.26E+07±2.76E+06	8.99E+07±5.25E+06	4.11E+07±3.03E+06	3.23E+09±4.35E+07	**5.80E+09**±4.51E+08	2.79E+09±9.91E+07	1.11E+09±2.98E+07
Kynurenine	**3.23E+08** ±2.43E+06	3.23E+08±5.72E+06	1.80E+08±8.01E+06	2.39E+08±1.10E+07	1.63E+08±3.27E+06	4.87E+07±1.70E+07	1.32E+08±6.19E+06	**1.83E+08**±3.50E+06
Adenosine	3.19E+09 ±1.37E+08	3.94E+09±3.20E+08	**4.21E+09**±1.06E+08	3.82E+09±1.03E+08	6.27E+08±1.79E+07	**1.22E+09**±9.62E+07	9.70E+08±5.77E+07	1.88E+08±6.94E+06
3-Methylcytidine	**4.25E+09** ±1.88E+07	6.64E+07±1.41E+06	n.d.	3.42E+09±1.52E+08	**9.14E+08**±1.12E+07	2.51E+08±6.78E+06	n.d.	3.38E+08±8.02E+06

UDP	3.56E+07±4.44E+05	**6.50E+07**±2.86E+06	4.59E+07±3.32E+06	1.04E+07±2.16E+06	2.48E+07±1.43E+06	**3.05E+07**±2.17E+06	3.03E+07±1.03E+07	6.71E+06±3.14E+05
GMP	3.23E+07±7.70E+05	**7.13E+07**±2.38E+06	5.38E+07±6.34E+06	9.55E+06±9.43E+05	**3.11E+07±**1.23E+06	2.74E+07±3.82E+06	1.98E+07±3.72E+06	3.52E+05±3.24E+05
Citric acid	**7.15E+08**±7.43E+07	4.42E+07±4.76E+07	n.d.	6.22E+08±5.09E+07	6.87E+10±8.73E+07	7.80E+10±5.96E+09	**8.04E+10**±2.19E+09	5.57E+10±3.33E+08
Fumarate	1.02E+08±2.38E+06	**1.55E+08**±8.06E+06	1.12E+08±8.48E+06	6.63E+07±3.27E+06	1.84E+08±5.49E+06	3.06E+08±3.55E+07	**3.32E+08**±1.03E+07	1.63E+08±3.09E+06
Succinate	6.88E+07±8.48E+05	**4.03E+08**±3.60E+07	3.09E+08±1.64E+07	1.82E+08±1.60E+07	5.06E+08±1.28E+07	1.16E+09±4.36E+07	5.94E+08±1.38E+08	**1.43E+09±**2.88E+07
Phosphocreatine	2.21E+07±1.17E+06	2.78E+07±2.65E+06	n.d.	**3.38E+07±**1.78E+06	2.31E+07±2.76E+06	n.d.	n.d.	**4.97E+07±**1.98E+06
Gluconate	**5.27E+08**±2.00E+07	2.52E+08±7.13E+06	1.55E+08±7.10E+06	1.24E+08±2.41E+07	1.33E+10±2.12E+08	8.36E+09±3.68E+08	1.83E+09±1.69E+08	**1.38E+10±**3.66E+08
Ribose	2.27E+06±2.82E+04	4.47E+06±1.08E+06	3.74E+06±1.09E+06	**5.68E+06±**4.45E+05	1.16E+09±3.27E+07	**1.23E+09**±1.04E+08	1.17E+09±2.30E+08	1.12E+09±5.27E+07
Choline chloride	4.42E+09±1.48E+08	n.d.	n.d.	**4.69E+09±**±2.09E+08	**3.87E+10±**3.99E+08	n.d.	n.d.	2.85E+10±4.10E+08
Average	1.17E+09	8.78E+08	9.95E+08	1.04E+09	9.27E+09	9.25E+09	9.42E+09	5.32E+09±

					**Xenobiotics and endogenous estrogen metabolites (RP)**			
Aflatoxin_B1	4.33E+08±1.08E+07	5.04E+08±2.75E+07	5.16E+08±7.54E+06	3.00E+08±1.21E+08	3.56E+08±7.10E+06	2.88E+08±1.50E+07	3.45E+08±1.07E+08	2.11E+08±1.03E+08
Alternariol	2.97E+08±1.47E+07	6.21E+08±2.88E+07	6.72E+08±4.71E+07	2.42E+08±2.01E+07	1.69E+08±3.39E+06	7.55E+08±5.55E+07	6.30E+08±1.52E+08	1.68E+08±6.79E+06
Ochratoxin A	1.08E+07±4.21E+05	1.26E+07±3.62E+05	1.03E+07±1.84E+06	3.42E+06±3.26E+05	4.41E+06±5.90E+05	9.40E+06±9.72E+05	8.64E+06±2.13E+06	4.20E+05±2.15E+05
Toxin T2	1.31E+06±6.78E+05	5.53E+06±6.17E+05	5.54E+06±4.00E+05	3.27E+06±5.26E+05	3.87E+05±1.98E+05	1.23E+07±1.72E+06	4.84E+07±3.19E+07	8.85E+06±8.41E+05
2-Hydroxyestradiol	2.51E+08±2.57E+06	1.50E+09±3.86E+07	1.74E+09±8.13E+07	7.93E+07±9.58E+06	3.22E+08±1.91E+07	1.78E+09±5.30E+07	1.77E+09±1.04E+08	9.44E+07±1.83E+06
2-Methoxyestrone	6.34E+07±8.67E+05	2.57E+08±1.27E+07	3.08E+08±1.89E+07	1.72E+05±1.19E+05	8.48E+07±1.28E+06	5.14E+08±4.34E+07	2.98E+08±2.12E+07	3.26E+05±2.30E+05
2-Naphtol	1.82E+08±1.88E+06	9.22E+08±3.27E+07	1.32E+09±3.29E+07	9.61E+07±2.17E+06	2.64E+08±4.79E+06	1.07E+09±3.56E+07	1.44E+09±5.79E+07	1.12E+08±2.45E+06
8-Prenylnaringenin	3.04E+07±9.83E+05	**7.85E+07**±5.00E+06	7.22E+07±3.81E+06	2.46E+07±2.01E+06	2.70E+07±7.18E+05	**1.55E+08**±1.39E+07	6.89E+07±3.24E+06	2.15E+07±9.92E+05
Alpha-zearalenol	4.16E+08±6.37E+06	**1.81E+09**±1.53E+08	1.47E+09±4.61E+07	6.44E+08±4.39E+07	4.11E+08±7.03E+06	**2.42E+09**±9.54E+08	1.31E+09±1.03E+08	5.45E+08±1.48E+07
Anisodamine	3.55E+09±7.62E+07	3.72E+09±1.67E+08	**4.06E+09±**2.49E+08	2.94E+09±1.44E+09	**1.36E+09±**1.36E+07	9.82E+08±6.19E+07	6.76E+08±3.89E+07	5.59E+08±1.70E+07

Benzophenone 2	3.06E+08±1.02E+07	3.89E+08±1.80E+07	**5.77E+08**±2.81E+08	1.35E+08±1.29E+07	8.87E+07±1.46E+06	1.96E+08±1.79E+07	**4.41E+08**±1.41E+08	1.19E+07±1.06E+06
Bisphenol_A	2.82E+07±8.89E+05	**2.52E+08**±4.77E+06	2.51E+08±6.53E+06	4.23E+05±1.59E+05	3.32E+07±4.08E+05	**2.16E+08**±3.28E+07	1.79E+08±2.09E+07	4.71E+05±3.14E+05
Cotinine	**5.52E+09**±1.05E+08	4.35E+09±1.03E+08	4.09E+09±2.97E+08	3.44E+09±1.58E+08	**1.98E+09**±5.48E+07	1.16E+09±5.34E+07	1.29E+09±4.76E+07	9.20E+08±1.63E+07
Coumestrol	5.88E+08±1.39E+07	8.24E+08±6.39E+07	**1.04E+09**±1.32E+08	3.23E+08±3.24E+07	2.57E+08±2.79E+06	**1.27E+09**±8.26E+07	1.14E+09±3.26E+08	2.18E+08±4.04E+06
Enterolactone	**5.19E+08**±2.37E+07	5.14E+08±2.33E+07	4.97E+08±3.83E+07	1.99E+08±1.30E+07	4.03E+08±4.39E+06	**6.57E+08**±3.48E+07	5.03E+08±	1.57E+08±7.09E+06
Estrone	2.44E+07±1.45E+06	**1.99E+08**±3.00E+06	1.95E+08±4.91E+06	n.d.	2.85E+07±1.45E+06	**4.34E+08**±3.75E+07	2.04E+08±8.12E+06	n.d.
Ethylparaben	3.67E+07±1.26E+06	4.78E+08±1.04E+07	**5.17E+08**±2.36E+07	3.12E+08±1.22E+07	4.46E+07±2.47E+06	**5.46E+08**±2.84E+07	5.42E+08±4.84E+07	2.45E+08±6.24E+06
Fenarimol	3.22E+07±2.09E+06	1.97E+07±6.06E+05	**5.35E+07**±1.65E+07	2.03E+07±5.19E+05	**3.13E+07**±2.30E+06	6.50E+06±2.60E+05	2.60E+07±1.47E+07	1.62E+07±1.15E+06
Jacobine	2.52E+09±5.17E+07	3.17E+09±1.35E+08	**3.31E+09**±2.04E+08	2.33E+09±8.96E+07	2.45E+08±8.78E+06	1.06E+09±1.27E+08	**1.69E+09**±4.01E+08	2.22E+08±1.19E+07
Methiocarb	2.71E+08±9.98E+06	3.96E+08±1.58E+07	**4.20E+08**±3.95E+07	2.19E+07±1.52E+06	2.63E+08±2.59E+06	**2.92E+08**±2.17E+07	2.74E+08±3.28E+07	1.98E+07±3.17E+05
PFOS	**2.37E+09**±3.76E+07	2.11E+09±1.34E+08	1.93E+09±5.63E+07	1.22E+09±4.07E+07	1.01E+09±1.51E+07	**1.20E+09**±3.37E+08	1.12E+09±1.65E+08	7.42E+08±1.43E+07
p-Hydrobenzoic acid	2.52E+09±9.77E+06	**1.94E+10**±9.64E+08	1.71E+10±1.26E+09	7.92E+09±5.41E+08	2.59E+09±8.16E+07	6.37E+09±4.44E+08	**8.13E+09**±2.35E+08	2.16E+09±4.78E+07
Tetrabrombisphenol A	1.87E+06±2.35E+05	**2.70E+07**±2.44E+06	2.70E+07±9.00E+06	1.47E+07±1.05E+06	2.34E+06±1.30E+05	1.67E+07±1.05E+06	**2.29E+07**±6.02E+06	1.29E+07±6.05E+05
Xanthohumol	2.44E+08±6.02E+06	1.46E+08±1.23E+07	**6.62E+08**±4.25E+08	4.60E+08±1.19E+07	2.23E+08±4.13E+06	1.26E+08±3.34E+07	1.29E+08±1.47E+08	**4.12E+08**±1.76E+07
Zearalenon-14-glucuronide	1.40E+07±4.02E+05	**2.70E+07**±9.12E+05	2.01E+07±3.58E+06	6.80E+06±5.58E+05	1.09E+07±4.28E+05	**1.94E+07**±2.08E+06	1.58E+07±2.31E+06	1.45E+06±4.26E+05
Average	8.09E+08	1.67E+09	1.63E+09	8.63E+08	4.09E+08	8.63E+08	8.92E+08	2.86E+08

Table S5 Limit of detection (LOD), retention time and the detected adducts (with the most abundant one in bold) in solvent and two complex biological matrices (urine and plasma) for the xenobiotics and human estrogens measured on the reverse phase column

**Compound**	**Neutral exact mass [m/z]**	**Species**	**Retention time [min]**			**LOD [µg/L]**		
			**Solvent**	**Plasma**	**Urine**	**Solvent**	**Plasma**	**Urine**
1-OH-pyrene	218.0732	**[M-H]$^{-}$**, [M+H]$^{+}$	11.3	11.3	11.3	4.13	4.57	1.89
16-Epiestriol	288.1725	**[M-H]$^{-}$**	7.5	7.5	7.6	0.21	2.08	4.19
16-Hydroxyestrone	286.1569	**[M-H]$^{-}$**	7.6	7.6	7.6	0.36	0.45	2.61
17-Epiestriol	288.1725	**[M-H]$^{-}$**	7.8	7.7	7.7	0.20	0.23	3.40
2-Hydroxyestradiol	288.1725	**[M-H]$^{-}$**	8.2	8.5	8.5	0.88	70.8	12.8
2-Methoxyestrone	300.1725	**[M-H]$^{-}$**, [M+H]$^{+}$	10.4	10.4	10.4	0.16	1.36	0.79
2-Naphtol	302.1882	**[M-H]$^{-}$**	8.7	8.6	8.6	0.21	0.23	5.42
2-Methoxyestradiol	150.1045	**[M-H]$^{-}$**	9.7	9.7	9.7	0.83	2.20	1.22
2-tert-Butylphenol	240.1514	**[M-H]$^{-}$**	11.0	11.0	11	0.08	0.09	1.73
3-Benzylidencampher	144.0575	**[M+H]$^{+}$**	12.5	12.5	12.5	0.20	0.59	2.33
4-Hydroxyestrone	302.1882	**[M-H]$^{-}$**	9.0	7.6	9.0	0.07	31.50	0.08
4-Methoxyestradiol	286.1569	**[M-H]$^{-}$**	9.4	9.4	9.4	2.17	2.39	1.53
4-Methoxyestrone	300.1725	**[M-H]$^{-}$**, [M+H]$^{+}$	10.2	10.2	10.2	0.18	0.19	0.22
4-Methylbenzylidencampher	254.1671	**[M+H]$^{+}$**	12.7	12.7	12.7	0.08	0.45	0.81
4-octylphenol	206.1671	**[M-H]$^{-}$**	12.7	12.7	12.7	0.42	0.50	0.50
4-tert-octylphenol	206.1671	**[M-H]$^{-}$**	12.3	12.3	12.3	0.41	0.46	0.53
8-Prenylnaringenin	340.1311	**[M-H]$^{-}$**, [M+H]$^{+}$	10.0	10.0	10.0	0.04	0.46	0.36
Aflatoxicol	296.0685	**[M+H]$^{+}$**	8.1	8.1	8.1	0.02	0.22	2.64
Aflatoxin B1	312.0634	**[M+H]$^{+}$**, [M-H]$^{-}$	7.8	7.8	7.8	0.04	0.05	3.52
Aflatoxin B2	314.0790	**[M+H]$^{+}$**, [M-H]$^{-}$	7.4	7.4	7.4	0.04	0.05	0.05
Aflatoxin G1	328.0583	**[M+H]$^{+}$**, [M-H]$^{-}$	7.4	7.4	7.4	0.05	0.05	0.05
Aflatoxin G2	330.0740	**[M+H]$^{+}$**, [M-H]$^{-}$	6.9	6.9	6.9	0.004	0.07	0.39
Aflatoxin M1	328.0583	**[M+H]$^{+}$**, [M-H]$^{-}$	6.5	6.4	6.4	0.004	0.01	0.03
Aflatoxin M2	330.0740	**[M+H]$^{+}$**, [M-H]$^{-}$	6.1	6.0	6.0	0.05	0.47	0.38
Aflatoxin P1	298.0477	**[M-H]$^{-}$**, [M+H]$^{+}$	6.2	4.3	6.2	0.04	4.50	0.04
Alpha-zearalanol	322.1780	**[M-H]$^{-}$**, [M+H]$^{+}$	9.1	9.1	9.1	0.04	0.43	0.39
Alpha-zearalenol-14-glucuronide	320.1624	**[M-H]$^{-}$**, [M+H]$^{+}$	5.2	4.7	5.2	4.96	4.91	0.34
Alpha-zearalenol	496.1945	**[M-H]$^{-}$**, [M+H]$^{+}$	9.3	9.3	9.3	0.05	0.50	7.02

Alternariol	258.0528	**[M-H]$^-$**, [M+H]$^+$	8.2	7.2	8.1	0.03	0.05	0.45
Alternariol monomethylether	272.0685	**[M-H]$^-$**, [M+H]$^+$	10.2	10.2	10.2	0.04	0.04	0.06
Anisodamine	305.1627	**[M+H]$^+$**	4.1	5.4	4.2	0.04	0.47	3.98
Beauvericin	783.4095	**[M+NH4]**$^+$	13.6	13.6	13.6	0.23	0.26	1.89
Benzophenone 1	214.0630	**[M-H]$^-$**, [M+H]$^+$	9.4	9.3	9.4	0.04	0.05	0.04
Benzophenone 2	246.0528	**[M-H]$^-$**	7.1	5.6	7.1	0.02	0.25	0.17
Benzylbutylphthalate	312.1362	**[M+H]$^+$**	12.4	12.4	12.4	0.22	1.11	1.93
Benzylparaben	228.0786	**[M-H]$^-$**	9.9	9.9	9.9	0.05	0.05	0.38
Beta-zearalanol	322.1780	**[M-H]$^-$**, [M+H]$^+$	8.5	8.5	8.5	0.04	0.06	0.40
Beta-zearalenol-14-glucuronide	320.1624	**[M-H]$^-$**, [M+H]$^+$	4.8	4.1	4.8	4.92	4.40	0.32
Beta-zearalenol	496.1945	**[M-H]$^-$**, [M+H]$^+$	8.6	8.6	8.6	0.04	0.12	44.37
Bisphenol A	228.1150	**[M-H]$^-$**	8.9	8.9	8.9	0.04	0.44	0.05
Bisphenol AF	336.0585	**[M-H]$^-$**	10.2	10.1	10.2	0.18	0.22	0.25
Bisphenol B	242.1307	**[M-H]$^-$**	9.6	9.6	9.6	0.34	0.45	3.94
Bisphenol C	256.1463	**[M-H]$^-$**	10.3	10.2	10.3	0.10	0.12	0.70
Bisphenol F	200.0837	**[M-H]$^-$**	7.8	7.8	7.8	0.08	0.13	8.19
Bisphenol S	250.0300	**[M-H]$^-$**	6.3	5.8	6.2	0.03	0.13	0.24
Butylparaben	194.0943	**[M-H]$^-$**	9.9	9.8	9.8	0.04	0.05	0.03
Citrinin	250.0841	**[M+H]$^+$**, [M-H]$^-$	5.2	4.6	5.2	0.03	0.47	3.99
Cotinine	176.0950	**[M+H]$^+$**	3.6	3.5	3.5	0.40	5.79	3.37
Coumestrol	268.0372	**[M-H]$^-$**, [M+H]$^+$	7.9	7.2	7.9	0.04	0.44	0.42
Daidzein	254.0579	**[M-H]$^-$**, [M+H]$^+$	6.8	5.8	6.8	0.02	0.02	0.57
Deoxynivalenol	296.1260	**[M-H]$^-$**, [M+H]$^+$	2.8	3.3	3.4	0.21	6.96	48.24
Dibutylphthalate	278.1518	**[M+H]$^+$**	12.5	12.5	12.5	5.74	3.82	5.51
E2-17-GlcA	448.2097	**[M-H]$^-$**	5.2	4.6	5.1	2.25	23.23	5.20
Enterodiol	302.1518	**[M-H]$^-$**	6.8	6.8	6.8	0.02	0.21	0.19
Enterolactone	298.1205	**[M-H]$^-$**, [M+H]$^+$	8.1	8.1	8.1	0.04	0.05	0.34
Equol	242.0943	**[M-H]$^-$**, [M+H]$^+$	8.0	7.9	7.9	0.06	0.43	4.00
Estradiol-3-sulfate	272.1776	**[M-H]$^-$**	6.2	5.8	6.2	0.10	0.84	0.63
Estradiol	278.1518	**[M-H]$^-$**	9.2	9.2	9.2	0.05	0.85	0.73
Estriol	352.1344	**[M-H]$^-$**	6.5	6.5	6.5	0.07	0.98	0.88
Estrone	288.1725	**[M-H]$^-$**	9.9	9.9	9.9	0.34	0.45	0.41
Ethinylestradiol	270.1620	**[M-H]$^-$**	9.7	9.7	9.7	0.23	0.26	2.50
Ethylparaben	296.1776	**[M-H]$^-$**	7.7	7.5	7.6	0.02	0.26	0.07
Fenarimol	330.0327	**[M+H]$^+$**, [M-H]$^-$	10.5	10.4	10.5	0.41	0.48	0.73

Formononetin	268.0736	**$[M+H]^+$**, $[M-H]^-$	8.8	8.7	8.7	0.01	0.23	0.15
Fumonisin B1	721.3885	**$[M+H]^+$**, $[M-H]^-$	6.1	4.3	6.1	0.39	4.47	9.62
Genistein	270.0528	**$[M-H]^-$**, $[M+H]^+$	7.9	7.1	7.9	0.02	0.05	2.10
Glycitein	284.0685	**$[M-H]^-$**, $[M+H]^+$	6.9	6.0	6.9	0.02	2.22	1.93
Isobutylparaben	194.0943	**$[M-H]^-$**	9.8	9.8	9.8	0.02	0.02	0.19
Isoxanthohumol	354.1467	**$[M-H]^-$**, $[M+H]^+$	8.7	8.8	8.8	0.04	0.45	0.45
Jacobine-N-oxide	351.1682	**$[M+H]^+$**, $[M-H]^-$	3.7	3.6	4.2	0.38	0.49	7.30
Jacobine	367.1631	**$[M+H]^+$**	4.6	4.2	3.6	0.04	0.33	3.68
Matairesinol	358.1416	**$[M-H]^-$**, $[M+H]^+$	8.0	7.9	8.0	0.09	0.90	6.69
MEHP	278.1518	**$[M-H]^-$**, $[M+H]^+$	9.1	9.1	9.1	0.82	1.27	11.76
Methiocarb	225.0823	**$[M+H]^+$**	10.2	10.2	10.2	0.17	1.36	0.70
Methylparaben	152.0473	**$[M-H]^-$**	6.5	5.6	6.5	0.06	0.12	0.24
Mono_buty_phthalate	222.0892	**$[M-H]^-$**, $[M+H]^+$	5.2	3.8	5.2	0.10	0.45	0.59
n-Butylbenzolsulfonamid	213.0823	**$[M-H]^-$**, $[M+H]^+$	9.5	9.5	9.5	0.19	0.24	0.23
Nivalenol	312.1209	**$[M-H]^-$**, $[M+H]^+$	1.4	1.8	n.a.	0.67	21.21	>30.00
Nonylphenol	220.1827	**$[M-H]^-$**	12.7	12.7	12.7	0.87	0.70	0.97
Ochratoxin A	403.0823	**$[M-H]^-$**, $[M+H]^+$	7.0	5.9	7.1	0.41	4.50	0.58
Ochratoxin Alpha	369.1212	**$[M-H]^-$**, $[M+H]^+$	4.9	4.2	4.9	0.41	0.73	1.43
Ochratoxin B	256.0139	**$[M-H]^-$**, $[M+H]^+$	6.1	5.0	6.0	0.03	0.46	0.41
Octylmethoxycinnamate	290.1882	**$[M+H]^+$**	13.1	13.1	13.1	2.83	38.78	18.89
p-Hydrobenzoic_acid	138.0317	**$[M-H]^-$**	1.8	1.7	1.8	0.23	0.2	0.15
PFOA	413.9737	**$[M-H]^-$**	8.1	7.9	88.6	0.20	0.23	0.13
PFOS	499.9375	**$[M-H]^-$**	9.6	9.4	9.6	0.09	1.36	0.34
PhIP	224.1062	**$[M+H]^+$**, $[M-H]^-$	6.8	6.8	6.8	0.04	0.38	10.33
Prochloraz	375.0308	**$[M+H]^+$**	11.7	11.7	11.7	0.04	0.46	0.52
Propylparaben	180.0786	**$[M-H]^-$**, $[M+H]^+$	8.9	8.8	8.9	0.01	0.02	0.02
Resveratrol	228.0786	**$[M-H]^-$**, $[M+H]^+$	6.6	6.5	6.6	0.41	2.36	0.55
Riddeliin-N-oxide	349.1525	**$[M+H]^+$**, $[M-H]^-$	3.6	3.5	4.1	0.19	3.18	3.24
Riddeliin	365.1475	**$[M+H]^+$**, $[M-H]^-$	3.9	4.6	3.5	0.05	0.38	1.75
Scolpolamine	303.1471	**$[M+H]^+$**, $[M-H]^-$	4.6	4.6	4.4	0.02	0.23	1.77
Sterigmatocystein	324.0634	**$[M+H]^+$**, $[M-H]^-$	10.8	10.8	10.8	0.04	0.46	0.53

Tentoxin	414.2267	**[M+H]+**, [M-H]-	8.3	8.2	8.3	0.04	0.50	4.06
Tetrabrombisphenol A	539.7571	**[M-H]-**	12.2	12.2	12.2	1.27	8.50	7.32
Toxin T2	466.2203	**[M+H]+**	9.8	9.8	9.8	0.40	4.81	4.55
trans-3-OH-cotinine	192.0899	**[M+H]+**	2.0	2.0	2.0	0.38	3.51	14.01
Triclosan	287.9512	**[M-H]-**	12.2	12.2	12.2	0.05	0.43	0.43
Xanthohumol	354.1467	**[M-H]-**, [M+H]+	11.6	11.6	11.5	0.01	0.20	0.16
Zearalanone	318.1467	**[M-H]-**, [M+H]+	10.2	10.2	10.2	0.04	0.05	0.05
Zearalenone-14-glucuronide	320.1624	**[M-H]-**	5.7	5.2	5.7	0.39	5.01	0.36
Zearalenone-14-sulfate	494.1788	**[M-H]-**	6.9	6.6	6.9	0.08	0.44	21.05
Zearalenone	398.1035	**[M-H]-**, [M+H]+	10.3	10.3	10.3	0.04	0.21	0.42

Table S6 Limit of detection (LOD), retention time and the detected adducts with the most abundant one in bold in solvent and two matrices (urine and plasma) for endogenous metabolites on the HILIC column. Not detected compounds are marked with n.d. (not detected) and bg indicates analytes where the background signal was too high for LOD determination

Compound	Neutral exact mass [m/z]	Species	Retention time [min]			LOD [µM]		
			Solvent	Urine	Plasma	Solvent	Urine	Plasma
1-Methylhydantoin	114.0429	**$[M-H]^-$**	5.2	5.2	5.2	0.09	**bg**	3
1-Methylnicotinamide	136.0637		Not detected					
2-(Carbamoylamino)butanedioic acid	176.0433	**$[M-H]^-$**, $[M+H]^+$	3.9	4.3	4.9	0.2	1	0.3
2'-Deoxyadenosine 5'-monophosphate	331.0682	**$[M+H]^+$**, $[M-H]^-$	2	2.1	2.6	0.003	0.4	0.05
2'-Deoxycytidine	227.0906	**$[M-H]^-$**, $[M+H]^+$	2.7	2.7	2.7	0.004	0.3	0.4
2-Deoxycytidine 5'-Monophosphate	307.0569	**$[M-H]^-$**, $[M+H]^+$	3.3	4	4.6	0.01	0.3	0.3
2'-Deoxyuridine	228.0746	**$[M-H]^-$**, $[M+H]^+$	2	2	2	0.01	0.03	0.03
2-Phosphoglyceric acid + 3-Phosphoglyceric acid	185.9929	**$[M-H]^-$**, $[M+H]^+$	8.0-8.7			0.03	0.3	0.3
3'AMP	347.0631	**$[M+H]^+$**, $[M-H]^-$	2.1	2.2	4.2*	0.01	3	0.3
3-Methyl-2-oxovaleric acid	130.0630	**$[M-H]^-$**	1.1	1.2	1.1	0.02	0.08	bg
3-Methylcytidine	257.1012	**$[M-H]^-$**, $[M+H]^+$	2.4	2.2	2.1	1	bg	1
4-Hydroxy-proline	131.0582	**$[M-H]^-$**, $[M+H]^+$	4.4	4.4	4.3	0.05	0.2	0.05
5'AMP	347.0631	**$[M+H]^+$**, $[M-H]^-$	2.5	2.6	4.2*	0.01	0.3	0.03
5'-Deoxy-5'-Methylthioadenosine	297.0896	**$[M+H]^+$**, $[M-H]^-$	1.6	1.6	1.6	0.004	0.004	0.005
5-Methyluridine	258.0852	**$[M-H]^-$**, $[M+H]^+$	2	2	2	0.004	0.04	0.05
6-Phosphogluconate	276.0246	**$[M-H]^-$**, $[M+H]^+$	8.5-8.7	8-8.4	7.5-8.2	0.1	0.3	0.3
Adenine	135.0545	**$[M+H]^+$**, $[M-H]^-$	2.3	2.3	2.3	0.004	0.01	0.004
Adenosine	267.0968	**$[M+H]^+$**, $[M-H]^-$	2.1	2.2	2.1	0.004	0.01	0.006
Adenosine 3',5'-cyclic monophosphate	329.0525	**$[M-H]^-$**, $[M+H]^+$	1.9	1.9	1.9	0.004	0.3	0.05
Adenosine 5'-triphosphate	506.9957	**$[M+H]^+$**, $[M-H]^-$	9.0-9.4	8.9-9.1	n.d.	0.3	0.3	n.d.
Adenosine diphosphate	427.0294	**$[M-H]^-$**, $[M+H]^+$	8.7-9	8.5-8.7	8.3-8.6	0.3	0.5	0.3
Alanine	89.0477	**$[M+H]^+$**, $[M-H]^-$	4.6	4.5	4.7	0.4	bg	bg
alpha-Aminoadipic acid	161.0688	**$[M+H]^+$**, $[M-H]^-$	4	4	4.8	0.04	bg	bg
alpha-Ketoglutarate	146.0215	**$[M-H]^-$**	2.8	2.9	3.3	0.04	bg	bg
Arginine	174.1117	**$[M-H]^-$**, $[M+H]^+$	No calibration possible					
Argininosuccinic acid	290.1226	**$[M+H]^+$**, $[M-H]^-$	5	5.2	5.2	0.004	0.2	0.02
Asparagine	132.0535	**$[M-H]^-$**, $[M+H]^+$	5.1	5.2	5.2	0.04	bg	0.2

Compound	Neutral exact mass [m/z]	Species	Retention time [min]			LOD [µM]		
			Solvent	Urine	Plasma	Solvent	Urine	Plasma
Aspartate	133.0375	**[M-H]⁻**, [M+H]⁺	4.1	4.2	4.8	0.04	0.3	0.4
Betaine	117.0790	**[M+H]⁺**, [M-H]⁻	2.8	2.7	2.8	0.04	bg	bg
Biotin	244.0882	**[M+H]⁺**, [M-H]⁻	1.5	1.4	1.3	0.002	0.03	0.02
Carnitine	161.1052	**[M+H]⁺**	4.1	4.1	4	0.03	bg	bg
Choline chloride	104.1075		Not detected					
cis-Aconitate	174.0164	**[M-H]⁻**	3.2-3.4	3.3-3.5	5-5.1	0.04	bg	bg
Citrate + Isocitrate	192.0270	**[M-H]⁻**	8.0-8.9	4.9-5	7.2-8.2**	1	bg	1
CMP	323.0519	**[M-H]⁻**, [M+H]⁺	4.2	4.2	4.8	0.04	0.3	0.2
Cysteic acid	169.0045	**[M-H]⁻**, [M+H]⁺	3.9-4.1	4.3	4.8	0.01	0.03	0.1
Cysteine	121.0197	**[M-H]⁻**, [M+H]⁺	6.2	n.d.	n.d.	1	bg	bg
Cysteinyl-glycine	178.0412	**[M-H]⁻**, [M+H]⁺	5.7	5.7	5.7	6	bg	bg
Cystine	240.0238	**[M+H]⁺**, [M-H]⁻	6.4	6.4	6.4	0.4	bg	bg
Cytidine	243.0855	**[M+H]⁺**, [M-H]⁻	3.4	3.3	3.3	0.03	0.03	0.1
Cytidine 5'-triphosphate	482.9845	**[M+H]⁺**, [M-H]⁻	8.7	8.8	n.d.	0.4	2	n.d.
Cytosine	111.0433	**[M+H]⁺**, [M-H]⁻	3.4	3.3	3.3	0.01	1	0.1
Deoxyguanosine triphosphate	506.9957	**[M+H]⁺**, [M-H]⁻	9.1	9.1	n.d	0.3	1	n.d.
Dihydroxyacetonephosphate	169.9980	**[M-H]⁻**, [M+H]⁺	3.6	3.9	4.8	0.4	0.4	2.5
Dihydroxyisovalerate	134.0579	**[M-H]⁻**	1.3	1.4	1.3	0.03	0.03	0.03
Erythritol	122.0579	**[M-H]⁻**	3.1	3.1	3.1	0.04	bg	0.4
Erythrose-4-phosphate	200.0086	**[M-H]⁻**	3.6-4.6	4-4.9	5.1-5.2	0.4	bg	1.7
Flavinadenin dinucleotide	785.1571	**[M+H]⁺**, [M-H]⁻	1.8	2.2	2.2	0.4	0.4	0.5
Hexose (Frucose, Galactose, Mannose, Glucose, Inositol)	180.0634	**[M-H]⁻**	5.5	5.4	5.5	0.5	bg	bg
Fructose-1,6-bisphosphate	339.9961	**[M-H]⁻**, [M+H]⁺	9	8.6	n.d.	0.3	1	n.d.
Hexose-6-phosphate (Fructose-6-phosphate, Glucose-1-phosphate, Glucose-6-phosphate)	260.0297	**[M-H]⁻**, [M+H]⁺	4.5-5	4.9-5	5.1-5.2	0.04	0.01	0.04
Fumarate	116.0110	**[M-H]⁻**	4.3	4.4	5	0.4	0.6	0.04
Gluconate	196.0583	**[M-H]⁻**	3.1-3.4	4	4.7	0.04	bg	0.5
Glutamate	147.0532	**[M-H]⁻**, [M+H]⁺	4	4.1	4.8	0.4	0.4	bg
Glutamine	146.0691	**[M+H]⁺**, [M-H]⁻	4.9	4.9	4.9	0.04	bg	bg
Glutamyl-cysteine	250.0623	**[M+H]⁺**, [M-H]⁻	3.1	3.2	5.5	0.4	bg	bg
Glutathione, oxidized	612.1519	**[M+H]⁺**, [M-H]⁻	5.3	5.3	5.5	0.4	1	0.5
Glutathione, reduced	307.0838	**[M-H]⁻**, [M+H]⁺	3.1-3.7	3.3-4	5.4	0.5	0.5	1
Glycine	75.0320	**[M-H]⁻**, [M+H]⁺	5.1	5.1	5.1	0.4	bg	bg

Compound	Neutral exact mass [m/z]	Species	Retention time [min]			LOD [µM]		
			Solvent	Urine	Plasma	Solvent	Urine	Plasma
Glyoxylic acid	74.0004		Not detected					
GMP	363.0580	**[M-H]$^-$**, [M+H]$^+$	4.7	5	5.1	0.04	0.4	0.05
Guanidineacetic acid	117.0538	**[M+H]$^+$**, [M-H]$^-$	4.8	5	5	0.4	bg	0.4
Guanine	151.0494	**[M+H]$^+$**, [M-H]$^-$	3.5	3.5	3.4	0.004	0.01	0.01
Guanosine + Isoguanosine	283.0917	**[M+H]$^+$**, [M-H]$^-$	3.6-3.8	3.6-3.8	3.6-3.8	0.01	0.30	0.01
Guanosine 3',5'-cyclic monophosphate	345.0474	**[M+H]$^+$**, [M-H]$^-$	2.6	2.6	2.6	0.004	0.3	0.5
Guanosine 5'-diphosphate	443.0243	**[M-H]$^-$**, [M+H]$^+$	8.8-9.0	8.5-8.8	8.8	0.3	0.3	30
Guanosine 5'-triphosphate	522.9907	**[M+H]$^+$**, [M-H]$^-$	8.7	8.7	n.d.	0.4	1.6	n.d.
Histidine	155.0695	**[M+H]$^+$**, [M-H]$^-$	7.6	6.9	7.1	0.05	bg	bg
Homocysteine	135.0354	**[M+H]$^+$**, [M-H]$^-$	4.2-5.9	4.2.59	4.3-5.8	0.3	0.3	1
Homoserine + Threonine	119.0582	**[M+H]$^+$**, [M-H]$^-$	4.8-5	4.8-5	4.7-5	0.04	bg	bg
Hydroxyglutaric acid	148.0372	**[M-H]$^-$**	2.6	2.7	3.5	0.04	bg	0.4
Inosine	268.0808	**[M-H]$^-$**, [M+H]$^+$	2.8	2.7	2.7	0.01	0.3	0.03
Inosine 5'-monophosphate	348.0471	**[M-H]$^-$**, [M+H]$^+$	3.8	4.1	4.8	0.03	0.3	0.4
Isoleucine + Leucine	131.0946	**[M-H]$^-$**, [M+H]$^+$	2.8-3	2.6-2.7	2.8-2.9	0.04	bg	bg
Ketoisovalerate	116.0473	**[M-H]$^-$**	1.2	1.3	1.2	0.02	0.8	0.3
Kynurenine	208.0848	**[M+H]$^+$**, [M-H]$^-$	3	3	2.9	0.004	0.2	0.03
Lactate	90.0317	**[M-H]$^-$**	1.7	1.7	1.7	0.5	0.6	2
L-Citrulline	175.0957	**[M+H]$^+$**, [M-H]$^-$	5.2	5.2	5.2	0.4	bg	3
L-Cystathionine	222.0674	**[M+H]$^+$**, [M-H]$^-$	6.3	6.3	6.3	0.04	0.04	0.004
L-Ornithine	132.0899	**[M-H]$^-$**, [M+H]$^+$	5.2	5.2	5.2	0.007	0.36	bg
Lysine	146.1055	**[M+H]$^+$**, [M-H]$^-$	12.6	12.6	12.1	1	bg	bg
Malate	134.0215	**[M-H]$^-$**	3.2	3.4	4.6	0.003	0.07	0.06
Mannitol	182.0790	**[M-H]$^-$**	4.1	4.1	4.1	0.4	bg	bg
Mannitol 1-phosphate	262.0454	**[M-H]$^-$**, [M+H]$^+$	4.5	4.9	5.1	0.04	0.35	0.4
Melatonine	232.1212	**[M+H]$^+$**, [M-H]$^-$	1.4	1.3	1.3	0.001	0.03	0.1
Methionine	149.0510	**[M+H]$^+$**, [M-H]$^-$	3.3	3.3	3.3	0.006	0.14	bg
Methionine sulfone	181.0409	**[M+H]$^+$**, [M-H]$^-$	4.2	4.3	4.2	0.01	bg	bg
Mevalonic acid	148.0736	**[M-H]$^-$**	1.3	1.3	1.3	0.01	1.00	0.3
N4-Acetylcytidine	285.0961	**[M+H]$^+$**, [M-H]$^-$	2	1.9	1.9	0.004	0.04	0.5
N-Acetyl-Asp-Glu	304.0906	**[M-H]$^-$**, [M+H]$^+$	4.7	4.7	5.2	0.04	1.00	bg
N-Acetyl-L-aspartic acid	175.0482	**[M-H]$^-$**, [M+H]$^+$	2.5	2.6	2.8	0.004	bg	0.08
N-Acetyl-serine	147.0532	**[M-H]$^-$**, [M+H]$^+$	1.9	1.9	2	0.003	0.3	0.05

Compound	Neutral exact mass [m/z]	Species	Retention time [min]			LOD [µM]		
			Solvent	Urine	Plasma	Solvent	Urine	Plasma
NAD+	663.1091	**[M+H]+**, [M-H]-	3.5	4	4.6	0.04	0.4	0.4
NADH	665.1248	**[M+H]+**, [M-H]-	3.1	n.d.	n.d.	0.1	n.d.	n.d.
NADP+	743.0755	**[M+H]+**, [M-H]-	6.3-6.9	6.3-6.9	6.7-7.2	0.1	1.00	1
NADPH	745.0911	**[M+H]+**, [M-H]-	7-7.5	6.6-7.2	7.4	1	3.00	3
Nicotinamide	122.0480	**[M+H]+**, [M-H]-	1.9	1.9	1.9	0.01	0.01	0.01
Octopamine	153.0790		Not detected					
Oxaloacetic acid	132.0059	**[M-H]-**	5.9	n.d.	n.d.	3	n.d.	n.d.
Palmitic acid	256.2402	**[M-H]-**, [M+H]+	1.1	1.1	1.1	n.d.	n.d.	n.d.
Phenylalanine	165.0790	**[M-H]-**, [M+H]+	2.7	2.7	2.7	0.004	0.002	bg
Phosphocreatine	211.0358	**[M+H]+**, [M-H]-	4.3	4.3	5.2	0.03	0.08	0.4
Proline	115.0633	**[M+H]+**, [M-H]-	3.6	3.6	3.6	0.002	0.03	bg
Propionyl-L-carnitine	217.1314	**[M+H]+**, [M-H]-	2.4	2.4	2.4	0.001	0.1	0.04
Pseudouridine	244.0695	**[M-H]-**, [M+H]+	3.3	3.3	3.3	0.05	0.1	0.1
Pyruvate	88.01604	**[M-H]-**	1.4	1.5	1.4	0.04	bg	
Ribose	150.0528	**[M-H]-**	3.2	n.d.	n.d.	1	n.d	n.d.
Ribose-5-phosphate + Ribulose-5-phosphate	230.0192	**[M-H]-**, [M+H]+	3.9-4.1	4.1-4.2	4.9-5	0.04	0.06	0.4
S-(Adenosyl)-methionine	398.1372	**[M+H]+**, [M-H]-	4.3	4.3	4.3	0.3	bg	0.4
Sarcosine	89.04768	**[M+H]+**, [M-H]-	4.2	4.2	4.2	0.1	1	0.1
Sedoheptulose-7-phosphate	290.0403	**[M-H]-**, [M+H]+	4.7	5	5.2	0.01	0.1	0.1
Seleno-methionine	196.9955	**[M+H]+**, [M-H]-	3.3	3.3	3.2	0.004	0.2	0.4
Serine	105.0426	**[M-H]-**, [M+H]+	5.3	5.2	5.3	0.2	bg	bg
Serotonine	176.0950	**[M+H]+**, [M-H]-	1.4	1.3	1.4	0.007	0.04	0.04
Spermidine	145.1579		Not detected					
Spermine	202.2157		Not detected					
Succinate	118.0266	**[M-H]-**	1.9	1.9	2.1	0.04	0.3	0.04
Thiamine hydrochloride	265.1123		Not detected					
Thymidine	242.0903	**[M-H]-**, [M+H]+	1.5-1.9	1.5-1.9	1.5-1.9	0.01	0.1	0.03
Thymidine 5'-monophosphate	322.0566	**[M-H]-**, [M+H]+	2	2.1	2.4	0.04	0.04	0.04
Thymine	126.0429	**[M-H]-**, [M+H]+	1.9	1.9	1.9	0.003	0.06	0.04
Trehalose	342.1162	**[M-H]-**, [M+H]+	5.1	5.1	5.1	0.04	0.2	0.05
Tryptophan	204.0899	**[M-H]-**, [M+H]+	3.3	3.3	3.3	0.01	bg	bg
TTP (Thymidinetriphosphate)	481.9893	**[M-H]-**, [M+H]+	8.8-9	8.7	n.d.	0.3	0.4	n.d.
Tyrosine	181.0739	**[M-H]-**, [M+H]+	4.1	4.1	4.1	0.05	bg	bg

Compound	Neutral exact mass [m/z]	Species	Retention time [min]			LOD [µM]		
			Solvent	Urine	Plasma	Solvent	Urine	Plasma
UMP	324.0359	**[M-H]⁻**, [M+H]⁺	3.3	3.9	4.7	0.04	0.4	0.5
Uracil	112.0273	**[M-H]⁻**, [M+H]⁺	2.2	2.2	2.2	0.1	bg	0.05
Uridine	244.0695	**[M-H]⁻**, [M+H]⁺	2.4	2.4	2.4	0.05	bg	0.5
Uridine 5'-diphosphate	404.0022	**[M-H]⁻**, [M+H]⁺	8.2-8.6	8-8.4	8.2-8.5	0.03	0.4	0.5
Uridine 5'-triphosphate	483.9685	**[M-H]⁻**, [M+H]⁺	9	8.6-8.8	n.d.	1	0.5	n.d.
Valine	117.078979	**[M-H]⁻**, [M+H]⁺	3.6	3.6	3.6	0.04	bg	bg
Xanthine	152.033425	**[M-H]⁻**, [M+H]⁺	2.6	2.7	2.7	0.5	bg	0.4
Xylose	150.052824	**[M-H]⁻**	3.9	n.d.	n.d.	0.01	n.d.	n.d.

Table S7 Calibration parameter of analytes in all three matrices and used normalisation method

Compound	Normalization Method	Solvent			Urine			Plasma		
		Slope	Intercept	R^2	Slope	Intercept	R^2	Slope	Intercept	R^2
**Xenobiotics and human estrogens**										
1-OH-pyrene	Ratio to Zearalenone	1.61E-03	-9.18E-03	0.9586	3.25E-03	-3.21E-03	0.9519	0.001116	-1.85E-03	0.9298
16-Epiestriol	Ratio to Ethylparaben	1.68E-02	-1.18E-03	0.9863	1.95E-02	-1.36E-02	0.8805	0.014192	-4.63E-03	0.9905
16-Hydroxyestrone	Ratio to Ethylparaben	1.47E-02	1.30E-05	0.9926	1.46E-02	-4.86E-03	0.9893	0.012079	-2.85E-03	0.9828
17-Epiestriol	Ratio to Ethylparaben	1.63E-02	-1.87E-03	0.9976	1.28E-02	-1.34E-02	0.993	0.013044	-3.17E-03	0.9914
2-Hydroxyestradiol	Ratio to PFOA	6.81E-03	-4.79E-03	0.9759	5.62E-03	-2.11E-02	0.9889	0.0002	-4.83E-03	0.9972
2-Methoxyestrone	Ratio to Zearalenone	3.39E-03	-6.28E-04	0.9935	4.87E-03	-2.34E-03	0.9852	0.005489	-3.34E-03	0.9809
2-Naphtol	Ratio to Propylparaben	2.19E-02	8.78E-04	0.9954	2.90E-02	-3.40E-04	0.9964	0.024809	4.74E-02	0.9699
2-Methoxyestradiol	Ratio to Butylparaben	3.35E-03	-6.64E-05	0.9333	3.76E-03	4.84E-03	0.9541	0.005253	-6.31E-03	0.9719
2-tert-Butylphenol	Ratio to Zearalenone	2.25E-02	7.43E-05	0.979	3.00E-02	-1.20E-02	0.9939	0.027336	4.27E-03	0.9975
3-Benzylidencampher	Ratio to 4-tert-octylphenol	1.93E-02	-1.27E-04	0.8706	1.14E-02	1.37E-02	0.8856	0.011321	6.61E-02	0.8256
4-Hydroxyestrone	Ratio to PFOS	3.57E-01	-3.82E-02	0.9426	3.72E-01	-4.09E-02	0.9821	0.004316	-8.85E-03	0.9628
4-Methoxyestradiol	Ratio to Bisphenol A	4.19E-03	-2.86E-04	0.9947	4.35E-03	-1.14E-04	0.9912	0.006278	-3.96E-03	0.9956
4-Methoxyestrone	Ratio to Zearalenone	1.56E-02	-2.67E-04	0.9874	2.06E-02	-3.84E-03	0.9948	0.026236	-2.46E-03	0.9816
4-Methylbenzylidencampher	Ratio to 4-tert-ocytlphenol	5.00E-02	-5.72E-04	0.8719	2.23E-02	8.88E-03	0.9134	0.017001	8.94E-03	0.9474
4-octylphenol	Ratio to 4-tert-octylphenol	2.65E-02	2.39E-02	0.9845	2.83E-02	1.29E-02	0.995	0.022614	1.03E-02	0.9099
4-tert-octylphenol	Ratio to Heavy	2.14E-02	1.06E-02	0.9982	2.41E-02	9.47E-03	0.9858	0.021099	3.78E-02	0.9993
8-Prenylnaringenin	Ratio to Zearalenone	3.89E-02	-5.82E-04	0.9851	3.84E-02	-2.46E-03	0.9879	0.036337	-5.39E-04	0.989
Aflatoxicol	Ratio to Methylparaben	5.50E-02	2.75E-04	0.9189	3.77E-02	-5.28E-02	0.9822	0.07386	-1.58E-02	0.9839
Aflatoxin B1	Ratio to Aflatoxin M1	2.02E+00	2.91E-02	0.9187	1.30E+00	-2.46E+00	0.9051	2.1559	-2.10E-02	0.9343
Aflatoxin B2	Ratio to Aflatoxin M1	2.26E+00	9.80E-03	0.9263	2.78E+00	2.81E-02	0.9737	2.175	-6.83E-02	0.9443
Aflatoxin G1	Ratio to Aflatoxin M1	2.00E+00	-8.54E-05	0.9402	2.43E+00	1.76E-02	0.9485	1.2168	-2.91E-02	0.923
Aflatoxin G2	Ratio to Aflatoxin M1	1.94E+00	4.63E-03	0.9757	1.62E+00	-2.66E-02	0.9593	1.3938	-5.56E-02	0.9168
Aflatoxin M1	Ratio to heavy	3.06E+00	-1.20E-02	0.9778	2.74E+00	-1.83E-01	0.9939	3.1011	-9.60E-02	0.9745
Aflatoxin M2	Ratio to Aflatoxin M1	8.75E-01	-7.50E-03	0.8915	4.85E-01	-5.38E-02	0.9825	0.49819	-1.80E-02	0.9324
Aflatoxin P1	Ratio to Methylparaben	2.25E-01	-7.78E-04	0.9923	2.81E-01	-1.26E-02	0.9446	0.08747	1.97E-01	0.9896
Alpha-zearalanol	Ratio to Estradiol	9.08E-02	-1.84E-03	0.9711	7.30E-02	-2.31E-03	0.9915	0.049936	-1.02E-03	0.9818
Alpha-zearalenol-14-glucuronide	Ratio to Methylparaben	8.93E-03	3.57E-03	0.8883	3.14E-03	-1.66E-03	0.9741	0.002296	-8.86E-03	0.9601
Alpha-zearalenol	Ratio to Estradiol	1.08E-01	-4.39E-03	0.9629	9.28E-02	-1.66E-03	0.9916	0.082238	-1.08E-02	0.9177

Alternariol	Ratio to PFOS	2.61E-01	-1.07E-03	0.9824	1.14E-01	-2.36E-03	0.9737	0.25361	-1.16E-02	0.9917
Alternariol monomethylether	Ratio to 4-tert-octylphenol	9.88E-02	-1.67E-03	0.9731	8.61E-02	-1.61E-03	0.9788	0.068212	-1.75E-03	0.9731
Anisodamine	Ratio to Methylparaben	2.55E-01	2.40E-03	0.9722	1.28E-01	-8.90E-02	0.9824	0.38523	-1.33E-02	0.8888
Aristolactam					Calibration not possible					
Beauvericin	Ratio to 4-tert-octylphenol	4.86E-02	-8.25E-03	0.9174	7.19E-02	-2.00E-01	0.98	0.032683	-8.32E-03	0.9538
Benzophenone 1	Ratio to PFOS	2.80E-01	5.32E-04	0.9872	2.67E-01	-8.19E-03	0.9772	0.28372	-2.43E-03	0.9611
Benzophenone 2	Ratio to Methylparaben	1.15E-01	-1.44E-03	0.9551	7.01E-02	-6.87E-03	0.9539	0.14632	-4.51E-02	0.9655
Benzylbutylphthalate	Ratio to 4-tert-ocytlphenol	3.32E-02	8.42E-03	0.9222	9.71E-03	5.39E-03	0.9617	0.008178	-4.19E-04	0.9475
Benzylparaben	Ratio to Butylparaben	6.59E-02	-3.34E-03	0.988	5.06E-02	-5.07E-03	0.9771	0.06375	-1.39E-03	0.9944
Beta-zearalanol	Ratio to Propylparaben	6.28E-02	1.17E-03	0.9024	3.42E-02	-3.48E-03	0.9906	0.051118	1.83E-03	0.9086
Beta-zearalenol-14-glucuronide	Ratio to Methylparaben	7.82E-03	-4.48E-03	0.9064	1.29E-03	1.14E-02	0.9102	0.005261	-1.67E-02	0.9513
Beta-zearalenol	Ratio to Propylparaben	5.99E-02	5.02E-04	0.8991	3.18E-02	-1.96E-03	0.9833	0.034194	-1.80E-04	0.9575
Bisphenol A	Ratio to Heavy	1.19E-02	-2.23E-05	0.9999	1.12E-02	-1.30E-03	0.9888	0.012178	4.00E-02	0.9993
Bisphenol AF	Ratio to Bisphenol A	2.80E-02	2.71E-04	0.9668	2.54E-02	-1.46E-03	0.9827	0.032505	-8.16E-04	0.9954
Bisphenol B	Ratio to PFOS	3.57E-02	-4.03E-03	0.9618	2.91E-02	-6.75E-03	0.9712	0.045717	-1.89E-03	0.9934
Bisphenol C	Ratio to Zearalenone	1.22E-02	-9.99E-04	0.9923	1.31E-02	-5.10E-03	0.9956	0.020067	-2.55E-03	0.9942
Bisphenol F	Ratio to Ethylparaben	3.88E-02	-9.08E-04	0.9974	2.28E-02	-2.88E-02	0.9821	0.031502	8.22E-04	0.9869
Bisphenol S	Ratio to Methylparaben	2.05E-01	3.24E-03	0.9775	1.75E-01	3.94E-02	0.9759	0.1763	9.02E-03	0.9721
Butylparaben	Ratio to Heavy	6.83E-02	2.36E-03	0.9969	7.09E-02	-3.09E-03	0.9899	0.064319	2.93E-03	0.9966
Citrinin	Ratio to Monobutyl phthalate	1.70E-01	-3.33E-04	0.8203	1.71E-02	8.18E-02	0.9	0.1489	-1.21E-01	0.9711
Cotinine	Ratio to Aflatoxin M1	2.82E+00	1.36E+00	0.9122	2.01E+00	-7.97E-01	0.9232	0.34778	7.65E+01	0.9799
Coumestrol	Ratio to Ethylparaben	1.28E-01	4.36E-04	0.9941	5.43E-02	-5.45E-04	0.9962	0.09237	-7.51E-03	0.9992
Daidzein	Ratio to Methylparaben	1.96E-01	-4.38E-04	0.9852	1.19E-01	2.41E-03	0.9676	0.16985	1.86E-03	0.94
Deoxynivalenol	Ratio to heavy	1.98E-02	-6.10E-03	0.9976	4.94E-02	-1.32E+00	0.9669	0.040926	-1.44E-01	0.9385
Dibutylphthalate	Ratio to 4-tert-ocytlphenol	2.81E-02	5.55E-01	0.9008	1.41E-02	2.14E-01	0.9747	0.01098	1.62E-01	0.9694
E2-17-GlcA	Ratio to Methylparaben	1.11E-02	-2.52E-03	0.8939	5.38E-03	2.73E-02	0.9569	0.004422	-9.96E-03	0.9526
Enterodiol	Ratio to Methylparaben	1.00E-01	-9.10E-04	0.9312	7.52E-02	7.87E-04	0.9773	0.033148	-4.59E-04	0.9893
Enterolactone	Ratio to PFOA	1.61E-01	2.50E-03	0.908	5.38E-02	1.89E-02	0.9609	0.10994	1.06E-03	0.9786
Equol	Ratio to Ethylparaben	4.79E-02	-2.89E-03	0.991	2.09E-02	-1.36E-02	0.9878	0.034082	-2.45E-03	0.9615
Estradiol-3-sulfate	Ratio to Methylparaben	1.45E-02	-1.89E-04	0.9938	1.63E-02	1.40E-03	0.9576	0.015993	3.34E-03	0.9926
Estradiol	Ratio to heavy	2.20E-02	-6.40E-04	0.9982	2.27E-02	-5.02E-03	0.9944	0.022114	-2.03E-03	0.9955
Estriol	Ratio to Methylparaben	1.47E-02	9.33E-04	0.9745	1.35E-02	-6.54E-03	0.9882	0.013999	-1.41E-03	0.9716

Estrone	Ratio to Butylparaben	1.85E-02	-7.76E-04	0.9658	1.74E-02	-8.67E-03	0.9808	0.021578	-4.28E-03	0.9933
Ethinylestradiol	Ratio to Butylparaben	8.89E-03	-1.50E-03	0.9397	7.67E-03	-6.77E-03	0.9944	0.010913	-1.32E-03	0.9781
Ethylparaben	Ratio to Heavy	1.10E-01	6.77E-03	0.9984	1.17E-01	3.69E-03	0.9891	0.10319	5.92E-02	0.9958
Fenarimol	Ratio to Zearalenone	2.14E-02	-1.83E-04	0.9866	8.28E-03	-2.05E-03	0.9846	0.003512	-2.98E-03	0.9787
Formononetin	Ratio to Propylparaben	1.80E-01	-8.03E-04	0.9815	8.18E-02	-1.89E-02	0.9485	0.038494	-1.15E-02	0.9413
Fumonisin B1	Ratio to Aflatoxin M1	1.91E-01	-2.80E-02	0.8005	1.48E-01	4.04E-01	0.9337	No calibration possible		
Genistein	Ratio to Ethylparaben	1.54E-01	-6.08E-04	0.9859	5.72E-02	1.95E-02	0.9893	0.12658	-1.28E-02	0.9832
Glycitein	Ratio to Methylparaben	8.32E-02	-2.24E-03	0.9688	3.48E-02	-1.03E-02	0.9772	0.12332	-1.98E-01	0.9906
Isobutylparaben	Ratio to Butylparaben	1.22E-01	1.01E-03	0.9935	1.11E-01	-3.43E-04	0.9973	0.1271	-1.60E-03	0.9792
Isoxanthohumol	Ratio to Propylparaben	2.86E-02	-1.37E-03	0.9948	1.73E-02	-1.00E-02	0.9761	0.034054	-1.96E-02	0.8796
Jacobine-N-oxide	Ratio to Monobutyl phthalate	8.49E-02	-1.77E-02	0.978	7.99E-02	-4.18E-01	0.9838	0.09504	-8.18E-02	0.9166
Jacobine	Ratio to Monobutyl phthalate	2.75E-01	-1.35E-02	0.9297	2.44E-02	1.92E-01	1	0.33514	-1.88E-01	0.9975
Matairesinol	Ratio to PFOA	2.54E-02	-2.59E-03	0.9606	1.27E-02	-1.50E-02	0.988	0.022283	-1.25E-02	0.9585
MEHP	Ratio to Heavy	1.22E-02	3.32E-03	0.9983	1.20E-02	-1.10E-01	0.9921	No calibration possible		
Methiocarb	Ratio to Zearalenone	8.69E-03	-4.78E-04	0.9276	4.68E-03	4.12E-03	0.8552	0.008178	-1.37E-03	0.9892
Methylparaben	Ratio to Heavy	1.15E-01	5.44E-03	0.9982	1.22E-01	3.10E-02	0.996	0.11344	1.97E-01	0.9991
Monobuty phthalate	Ratio to Heavy	1.45E-02	3.04E-04	0.9985	1.40E-02	-6.46E-03	0.9855	0.016108	8.49E-02	0.9731
n-Butylbenzolsulfonamid	Ratio to PFOS	3.03E-02	1.75E-02	0.9912	3.22E-02	1.95E-02	0.9914	0.048941	8.67E-02	0.9909
Nivalenol	Ratio to Methylparaben	7.89E-03	-1.18E-02	0.9924	No calibration possible			0.005436	-1.19E-01	0.9989
Nonylphenol	Ratio to 4-tert-ocytlphenol	2.20E-02	1.16E-01	0.9624	2.78E-02	1.07E-01	0.9973	0.023142	1.95E-01	0.9829
Ochratoxin A	Ratio to Methylparaben	7.66E-03	-8.02E-04	0.9761	8.01E-03	-4.28E-03	0.9834	0.007521	-3.63E-04	0.9895
Ochratoxin Alpha	Ratio to Methylparaben	5.57E-02	-5.61E-04	0.9135	6.36E-02	-6.35E-04	0.8304	0.016293	-1.08E-02	0.9777
Ochratoxin B	Ratio to Methylparaben	2.74E-02	-9.63E-04	0.9847	2.10E-02	-1.38E-04	0.9466	0.015959	-4.81E-03	0.9017
Octylmethoxycinnamate	Ratio to Aflatoxin M1	3.38E-02	9.80E-02	0.8399	2.45E-02	8.46E-02	0.9442	0.032943	-2.67E+00	0.938
p-Hydrobenzoicacid	Ratio to Heavy	1.23E-03	6.69E-04	0.9962	1.16E-03	2.06E-01	0.9928	0.001174	4.25E-02	0.9969
PFOA	Ratio to Heavy	1.03E-01	1.64E-03	0.9814	1.13E-01	-1.96E-02	0.9957	0.10455	1.97E-01	0.9744
PFOS	Ratio to Heavy	5.08E-02	-1.73E-03	0.9974	4.81E-02	2.47E-03	0.9944	0.045655	5.01E-01	0.9958
PhIP	Ratio to Methylparaben	3.14E-01	1.01E-02	0.9805	2.83E-01	-6.58E-03	0.9878	0.093316	-1.57E-02	0.9552
Prochloraz	Ratio to Zearalenone	4.37E-02	-1.78E-03	0.9687	3.29E-02	-1.91E-03	0.9556	0.024809	-4.13E-03	0.9711
Propylparaben	Ratio to Heavy	1.17E-01	2.85E-03	0.9996	1.19E-01	7.19E-03	0.9985	0.11788	7.97E-03	0.9939
Resveratrol	Ratio to Methylparaben	2.05E-02	-1.38E-02	0.943	2.13E-02	-1.17E-02	0.9956	0.003321	-6.01E-03	0.9348

Riddeliin-N-oxide	Ratio to Monobutyl phthalate	6.15E-02	-1.90E-02	0.9759	3.24E-02	3.01E-02	0.9742	0.056873	-3.41E-02	0.942
Riddeliin	Ratio to Monobutyl phthalate	1.99E-01	6.96E-03	0.9161	3.52E-02	-1.44E-01	0.9772	0.19144	-6.45E-02	0.97
Scolpolamine	Ratio to Monobutyl phthalate	1.62E-01	-4.27E-03	0.9476	2.62E-02	-7.62E-02	0.9437	0.21479	-3.92E-02	0.9534
Sterigmatocystein	Ratio to Zearalenone	5.89E-02	-1.30E-03	0.9365	2.78E-02	-2.34E-03	0.9969	0.010396	-2.67E-03	0.9931
Tentoxin	Ratio to Ethylparaben	5.22E-02	-3.63E-04	0.9587	3.74E-02	4.35E-03	0.9627	0.04868	-9.52E-03	0.9899
Tetrabrombisphenol A	Ratio to 4-tert-ocytlphenol	1.17E-03	-3.13E-04	0.9739	1.99E-03	1.06E-03	0.9954	0.001311	9.25E-03	0.9127
Toxin T2	Ratio to Butylparaben	1.93E-02	-6.80E-03	0.9552	6.96E-03	-4.90E-03	0.9872	0.003815	-4.54E-03	0.9499
trans-3-OH-cotinine	Ratio to Aflatoxin M1	9.87E-01	3.79E-01	0.968	8.89E-02	2.64E+01	0.9847	0.050591	4.82E+00	0.9462
Triclosan	Ratio to 4-tert-ocytlphenol	2.83E-02	1.10E-04	0.9927	2.29E-02	4.03E-04	0.9972	0.024914	3.03E-04	0.9883
Xanthohumol	Ratio to Zearalenone	4.44E-02	8.03E-04	0.928	4.91E-02	1.05E-03	0.9494	0.047208	-1.35E-03	0.9845
Zearalanone	Ratio to Zearalenone	4.36E-02	-5.21E-04	0.992	4.06E-02	-8.47E-04	0.9914	0.041506	-1.12E-03	0.9932
Zearalenone	Ratio to Heavy	3.93E-02	-6.69E-04	0.9993	3.99E-02	-5.56E-03	0.9986	0.039154	-2.78E-03	0.9988
Zearalenone-14-glucuronide	Ratio to Methylparaben	4.80E-03	-8.55E-04	0.9061	1.73E-03	-2.95E-03	0.9066	0.001513	-9.97E-03	0.9403
Zearalenone-14-sulfate	Ratio to Methylparaben	3.09E-02	-1.62E-03	0.9686	2.86E-02	-1.11E-02	0.998	3.39E-02	-1.66E-03	0.9921
**Endogenous metabolites excluding human estrogens**										
1-Methylhydantoin	Ratio to Heavy	3.96E-01	6.84E-02	0.9977	No calibration possible			6.67E-01	1.30E+01	0.9337
1-Methylnicotinamide	No calibration possible									
2-(Carbamoylamino)butanedioic acid	Ratio to surrogate Aspartic acid	3.45E-02	1.86E-03	0.9698	1.41E-02	7.45E-03	0.9172	No calibration possible		
2’-Deoxyadenosine 5’-monophosphate	Ratio to surrogate GMP	8.43E+00	-3.66E-03	0.99	1.30E+01	8.78E-01	0.9487	2.76E-01	-1.38E-02	0.9851
2'-Deoxycytidine	Ratio to surrogate Phenylalanine	6.78E-01	-1.30E-04	0.9976	1.25E-01	1.68E-01	0.9542	2.11E-01	1.60E-02	0.9639
2-Deoxycytidine 5'-Monophosphate	Ratio to surrogate UMP	9.07E-01	3.85E-03	0.9945	1.19E+00	9.72E-03	0.9446	4.24E-01	-7.73E-02	0.992
2'-Deoxyuridine	Ratio to surrogate Succinic acid	5.18E-01	1.13E-03	0.9768	3.34E-01	1.39E-02	0.9745	1.40E-01	-2.55E-03	0.9761
2-Phosphoglyceric acid + 3-Phosphoglyceric acid	Ratio to surrogate ADP	6.05E-01	3.42E-02	0.9901	7.92E+00	2.18E+00	0.9769	1.10E+00	-9.19E-02	0.9777
3'AMP	Ratio to surrogate 5'-AMP	1.49E-01	6.36E-05	0.9401	1.95E-01	2.89E-01	0.9035	2.09E-01	-1.11E-03	0.9673

3-Methyl-2-oxovaleric acid	Ratio to Heavy	6.15E+00	2.05E-01	0.994	1.03E+01	2.72E+01	0.968	No calibration possible		
3-Methylcytidine	Ratio to surrogate Adenine	1.35E-03	-9.80E-04	0.9717	No calibration possible			No calibration possible		
4-Hydroxy-proline	Ratio to surrogate Homoserin	6.94E-01	-2.97E-04	0.9423	3.76E+00	3.30E+00	0.9172	No calibration possible		
5'AMP	Ratio to Heavy	2.37E-01	4.27E-04	0.9928	3.79E-01	1.04E-02	0.9682	2.30E-01	-4.68E-03	0.9922
5'-Deoxy-5'-Methylthioadenosine	Ratio to Heavy	4.66E+00	2.65E-03	0.9976	5.21E+00	5.70E-01	0.9968	4.98E+00	1.62E-02	0.9991
5-Methyluridine	Ratio to surrogate Succinic acid	3.79E-01	3.42E-03	0.9967	1.75E+01	8.35E+00	0.9583	1.14E+01	2.14E-02	0.9915
6-Phosphogluconate	Ratio to surrogate ADP	1.14E-01	-8.96E-03	0.989	1.49E+00	-3.33E-01	0.9957	2.93E-01	-7.07E-02	0.9676
Adenine	Ratio to Heavy	2.48E+01	6.06E-02	0.9964	2.60E+01	3.31E+00	0.9733	2.93E+01	2.77E-01	0.9944
Adenosine	Ratio to Heavy	1.52E+01	3.97E-02	0.9983	4.10E-01	1.25E-01	0.9526	2.11E-01	3.45E-02	0.9727
Adenosine 3',5'-cyclic monophosphate	Ratio to surrogate Succinic acid	2.19E-01	-6.20E-04	0.9858	2.44E-01	3.55E-01	0.927	1.46E-01	-1.19E-02	0.9777
Adenosine 5'-triphosphate	Ratio to Heavy	6.64E-01	1.50E-01	0.9804	1.06E+00	-2.79E-01	0.9034	No calibration possible		
Adenosine diphosphate	Ratio to Heavy	3.36E-01	9.98E-02	0.995	3.80E-01	-1.53E-02	0.9898	2.96E-01	-9.85E-02	0.9718
Alanine	Ratio to Heavy	1.75E-02	2.56E-03	0.9939	No calibration possible			No calibration possible		
alpha-Aminoadipic acid	Ratio to surrogate Proline	1.32E-02	2.81E-04	0.9256	No calibration possible			No calibration possible		
alpha-Ketoglutarate	Ratio to Heavy	4.03E-01	-7.87E-04	0.994	No calibration possible			No calibration possible		
Arginine	Ratio to Heavy	No calibration possible			No calibration possible			No calibration possible		
Argininosuccinic acid	Ratio to Heavy	5.70E-01	-1.87E-03	0.9982	6.98E-01	3.12E+00	0.9657	5.85E-01	2.59E-02	0.9712
Asparagine	Ratio to Heavy	3.67E-01	-1.45E-03	0.9948	3.79E-01	9.05E+00	0.945	4.15E-01	9.91E+00	0.9629
Aspartate	Ratio to Heavy	4.63E-02	3.98E-04	0.9771	3.11E-02	6.26E-02	0.9629	4.70E-02	2.64E-01	0.9742
Betaine	Ratio to surrogate Phenylalanine	5.73E+01	6.33E-01	0.9859	No calibration possible			No calibration possible		
Biotin	Ratio to surrogate Pyruvic acid	4.67E+00	6.26E-02	0.9714	2.92E+00	3.68E+00	0.9098	9.97E-01	3.74E-02	0.934
Carnitine	Ratio to surrogate Glutamate	7.63E-01	2.90E-03	0.9949	No calibration possible			No calibration possible		
Choline chloride	No calibration possible									
cis-Aconitate	Ratio to Heavy	5.04E+01	-1.14E+00	0.9976	No calibration possible			3.70E+01	4.56E+00	0.9672
Citrate + Isocitrate	Ratio to Heavy	1.27E+00	2.02E-01	0.968	No calibration possible			1.74E+00	2.68E+00	0.9818
CMP	Ratio to surrogate Aspartic acid	1.82E-02	-2.80E-04	0.9884	1.38E-02	-8.56E-04	0.9748	9.23E-03	-9.04E-04	0.9767

Cysteic acid	Ratio to surrogate Aspartic acid	3.34E-02	3.77E-05	0.9803	2.56E-02	1.21E-04	0.9683	1.38E-02	-6.62E-04	0.9864
Cysteine	Ratio to surrogate Cystathionine	2.27E-03	-5.34E-04	0.9788	No calibration possible			No calibration possible		
Cysteinyl-glycine	Ratio to surrogate Serine	3.67E-03	-8.00E-03	0.906	No calibration possible			No calibration possible		
Cystine	Ratio to surrogate Cystathionine	3.97E-02	-1.95E-03	0.9958	No calibration possible			No calibration possible		
Cytidine	Ratio to surrogate Proline	4.09E-02	1.99E-04	0.9725	3.30E-02	8.76E-04	0.9946	9.70E-02	9.72E-04	0.9927
Cytidine 5'-triphosphate	Ratio to surrogate ATP	1.53E-01	-2.59E-02	0.9725	2.71E-01	-5.77E-01	0.9161	No calibration possible		
Cytosine	Ratio to surrogate Glutamate_pos	3.60E-01	3.32E-03	0.9733	1.63E-01	-4.70E-03	0.99	8.96E-01	1.77E-02	0.9801
Deoxyguanosine triphosphate	Ratio to surrogate ATP	6.64E-01	1.50E-01	0.9804	1.07E+00	-2.85E-01	0.9007	No calibration possible		
Dihydroxyacetonephosphate	Ratio to surrogate Valine	2.87E-02	-1.18E-03	0.9882	1.23E-02	4.95E-03	0.9755	1.25E-02	-2.49E-02	0.9665
Dihydroxyisovalerate	Ratio to surrogate 3-Methyl-2-oxovaleric acid	9.37E+00	-1.44E-01	0.989	1.34E+01	1.03E+01	0.9679	2.06E+01	1.56E+01	0.9718
Erythritol	Ratio to surrogate Valine	2.02E-01	6.66E-03	0.9928	No calibration possible			1.05E-01	1.01E+00	0.9255
Erythrose-4-phosphate	Ratio to surrogate Aspartic acid	4.78E-03	-1.11E-03	0.9605	6.00E-04	-1.00E-05	0.913	9.10E-04	-8.30E-04	0.9366
Flavinadenin dinucleotide	Ratio to surrogate Nicotinamide	3.76E-02	-9.45E-04	0.9269	1.34E-01	-3.53E-02	0.9293	1.27E-02	-9.59E-03	0.957
Hexose (Frucose, Galactose, Mannose, Glucose, Inositol)	Ratio to surrogate Trehalose	9.7598E-1	2.4878E-2	0.9882	No calibration possible			No calibration possible		
Fructose-1,6-bisphosphate	Ratio to surrogate ADP	7.08E-02	-3.17E-03	0.9852	1.03E-01	-1.46E-03	0.9335	No calibration possible		
Hexose-6-phosphate (Fructose-6-phosphate, Glucose-1-phosphate, Glucose-6-phosphate)	Ratio to surrogate Trehalose	3.36E+00	-2.21E-02	0.9792	2.81E+00	9.51E-01	0.991	1.65E+00	1.12E-01	0.9736
Fumarate	Ratio to Heavy	2.56E-01	-7.82E-04	0.9987	3.05E-01	6.44E-01	0.9566	2.58E-01	3.63E-01	0.9957
Gluconate	Ratio to surrogate Valine	1.06E+00	5.90E-02	0.9709	No calibration possible			4.52E+00	6.85E+00	0.9216
Glutamate	Ratio to Heavy	7.53E-03	9.71E-05	0.9993	No calibration possible			No calibration possible		
Glutamine	Ratio to Heavy	3.18E-02	4.29E-04	0.9991	No calibration possible			No calibration possible		
Glutamyl-cysteine	Ratio to surrogate Methionine	2.74E-01	-3.81E-03	0.9176	8.60E-02	-1.62E-02	0.8527	No calibration possible		
Glutathione, oxidized	Ratio to Heavy	1.84E-01	-1.12E-02	0.9939	2.33E-01	-1.35E-01	0.9786	1.64E-01	-1.40E-02	0.928

Glutathione, reduced	Ratio to surrogate Aspartic acid	1.09E-02	-4.97E-05	0.9233	6.31E-02	-3.31E-03	0.9361	6.64E-03	-2.67E-04	0.9824
Glycine	Ratio to Heavy	2.60E-01	1.21E-01	0.9925	No calibration possible			No calibration possible		
Glyoxylic acid	No calibration possible									
GMP	Ratio to Heavy	1.85E+00	-2.60E-02	0.9952	2.62E+00	-3.96E-01	0.9521	1.77E+00	-7.84E-02	0.9768
Guanidineacetic acid	Ratio to surrogate Threonine	1.64E+00	1.05E-01	0.9794	No calibration possible			2.31E+00	6.36E+00	0.9207
Guanine	Ratio to surrogate Glutamate_pos	1.07E-01	2.10E-03	0.9804	4.56E-02	1.91E-02	0.9148	1.77E-01	-4.35E-03	0.9507
Guanosine + Isoguanosine	Ratio to surrogate Proline	1.59E-01	2.14E-04	0.9715	5.35E-02	9.11E-03	0.9552	1.92E+00	1.45E-03	0.9934
Guanosine 3',5'-cyclic monophosphate	Ratio to surrogate 5'-AMP	2.57E-01	-1.22E-04	0.9827	2.56E-01	8.42E-03	0.9374	1.19E-01	-7.78E-02	0.9686
Guanosine 5'-diphosphate	Ratio to surrogate ADP	1.37E-01	1.93E-02	0.992	1.80E-01	-2.62E-02	0.9533	2.82E-02	-7.39E-02	0.9785
Guanosine 5'-triphosphate	Ratio to surrogate ATP	9.17E-02	-1.99E-02	0.9662	1.65E-01	-4.33E-01	0.9071	No calibration possible		
Histidine	Ratio to Heavy	1.81E-01	2.49E-02	0.9992	No calibration possible			No calibration possible		
Homocysteine	Ratio to surrogate S-Adenosylhomocysteine	8.40E-02	1.52E-02	0.9094	1.83E-01	-1.38E-02	0.9687	7.84E-02	-2.89E-02	0.9713
Homoserine + Threonine	Ratio to Heavy	3.74E-01	3.33E-03	0.9987	No calibration possible			No calibration possible		
Hydroxyglutaric acid	Ratio to Heavy	7.61E-01	9.21E-03	0.9937	9.37E-01	7.40E+00	0.9826	5.54E-01	3.73E-01	0.9462
Inosine	Ratio to surrogate Malic acid	9.05E-02	2.30E-05	0.9821	3.59E-02	3.31E-02	0.9088	5.46E-02	5.91E-04	0.987
Inosine 5'-monophosphate	Ratio to surrogate Aspartic acid	2.11E-02	-1.71E-04	0.9803	1.39E-02	-7.41E-04	0.9845	1.09E-02	-1.94E-03	0.9918
Isoleucine + Leucine	Ratio to Heavy	2.09E+00	4.24E-02	0.999	No calibration possible			No calibration possible		
Ketoisovalerate	Ratio to surrogate Pyruvic acid	1.10E+00	1.80E-01	0.983	9.95E-01	6.12E+00	0.9563	4.16E+00	2.31E+01	0.9961
Kynurenine	Ratio to surrogate Methionine	1.66E+00	1.22E-02	0.9668	3.30E-01	-1.27E-02	0.9585	1.58E+00	1.04E+00	0.9829
Lactate	Ratio to surrogate Pyruvic acid	8.68E-01	2.45E+00	0.9586	9.37E-01	1.94E+01	0.9238	1.77E+02	5.29E+02	0.9938
L-Citrulline	Ratio to Heavy	1.32E-01	2.71E-03	0.9934	No calibration possible			1.50E-01	5.35E+00	0.9629
L-Cystathionine	Ratio to Heavy	2.42E-01	-1.71E-03	0.9927	3.43E-01	1.59E+00	0.9675	2.78E-01	5.21E-02	0.9985
L-Ornithine	Ratio to surrogate Serine	1.53E-01	4.37E-03	0.9976	8.94E-03	1.72E-02	0.9554	No calibration possible		
Lysine	Ratio to Heavy	4.47E-02	1.98E-02	0.9797	No calibration possible			No calibration possible		

Malate	Ratio to Heavy	1.84E-01	2.35E-03	0.9984	2.33E-01	9.07E-01	0.9837	2.36E-01	3.44E-01	0.9289
Mannitol	Ratio to surrogate Tyrosine	1.82E+00	6.62E-02	0.9853	No calibration possible			No calibration possible		
Mannitol 1-phosphate	Ratio to surrogate Trehalose	1.02E+00	-2.27E-02	0.9731	1.00E+00	-2.16E-01	0.984	4.56E-01	-2.37E-02	0.9299
Melatonine	Ratio to surrogate Adenosine	2.02E+01	6.96E-03	0.948	2.12E+01	-2.63E-01	0.9221	3.38E+01	-1.44E+00	0.9317
Methionine	Ratio to Heavy	1.05E+00	7.05E-04	0.9937	1.14E+00	3.42E+00	0.9855	No calibration possible		
Methionine sulfone	Ratio to surrogate Tyrosine	3.21E+00	4.75E-03	0.9317	2.42E+00	1.78E+00	0.9608	2.75E+00	3.77E+00	0.9755
Mevalonic acid	Ratio to surrogate 3-Methyl-2-oxovaleric acid	4.98E+00	9.78E-02	0.987	8.85E+00	2.63E+01	0.9765	6.86E+00	7.42E+00	0.9898
N4-Acetylcytidine	Ratio to surrogate Adenosine	2.92E+00	-6.08E-03	0.9796	4.12E+00	5.92E+00	0.948	1.37E+00	-1.39E-01	0.9256
N-Acetyl-Asp-Glu	Ratio to surrogate Aspartic acid	6.62E-02	-2.67E-04	0.9636	5.58E-01	1.55E+00	0.9822	No calibration possible		
N-Acetyl-L-aspartic acid	Ratio to surrogate Hydroxyglutaric acid	5.51E-01	5.28E-03	0.9955	No calibration possible			1.11E+00	2.23E-01	0.9898
N-Acetyl-serine	Ratio to surrogate Succinic acid	3.52E-01	2.20E-03	0.9732	4.07E-01	2.07E+00	0.9893	1.72E-01	2.54E-01	0.9807
NAD+	Ratio to Heavy	8.04E-01	-3.43E-03	0.9473	1.14E+00	-2.09E-01	0.9675	1.39E+00	-3.41E-01	0.9226
NADH	Ratio to surrogate NAD+	9.70E-02	-3.49E-03	0.9771	No calibration possible			No calibration possible		
NADP+	Ratio to surrogate ADP	1.28E-01	-1.19E-02	0.9885	8.98E-02	-5.78E-02	0.9261	8.38E-01	-5.83E-01	0.9703
NADPH	Ratio to surrogate ADP	1.62E-02	-8.42E-03	0.9752	1.03E-01	-2.51E-01	0.9653	2.03E-02	2.72E-02	0.9133
Nicotinamide	Ratio to Heavy	2.50E+00	5.26E-03	0.9961	2.47E+00	6.24E-01	0.9852	2.37E+00	1.40E-01	0.9811
Octopamine	No calibration possible									
Oxaloacetic acid	Ratio to surrogate Citric acid	4.18E-04	-2.78E-04	0.9573	No calibration possible			No calibration possible		
Palmitic acid	No calibration possible	No calibration possible								
Phenylalanine	Ratio to Heavy	1.49E+00	2.57E-02	0.98	No calibration possible			No calibration possible		
Phosphocreatine	Ratio to surrogate Argininosuccinic acid	1.81E-01	-2.23E-03	0.9754	3.65E-01	4.61E-01	0.9598	2.23E-01	-1.25E-02	0.9948
Proline	Ratio to Heavy	6.05E-02	1.27E-03	0.9989	5.26E-02	8.98E-02	0.9781	-4.71E+00	1.29E+01	-447.114
Propionyl-L-carnitine	Ratio to surrogate Adenine	1.50E+01	5.44E-03	0.9835	7.64E+01	6.94E+01	0.9223	2.59E+01	2.35E+01	0.9623
Pseudouridine	Ratio to surrogate Valine	7.14E-01	4.75E-03	0.9814	6.55E-01	2.80E-01	0.9614	2.35E+00	3.35E+00	0.9829
Pyruvate	Ratio to Heavy	3.95E-01	1.46E-02	0.9921	4.93E-01	6.90E+00	0.9591	1.00E+00	2.28E+01	0.9375
Ribose	Ratio to surrogate Valine	5.46E-03	-3.72E-03	0.9633	No calibration possible			No calibration possible		

Ribose-5-phosphate + Ribulose-5-phosphate	Ratio to surrogate Aspartic acid	3.36E-02	-6.56E-04	0.9812	3.36E-02	3.06E-02	0.9814	1.44E-02	1.02E-02	0.9687
S-(Adenosyl)-methionine	Ratio to Heavy	8.56E-02	-6.27E-03	0.9987	No calibration possible			1.02E-01	4.11E-01	0.9751
Sarcosine	Ratio to surrogate S-Adenosylhomocysteine	1.68E+00	8.52E-02	0.9666	2.73E+00	3.50E+00	0.9443	2.54E+00	3.40E+00	0.9564
Sedoheptulose-7-phosphate	Ratio to surrogate ADP	2.05E-01	-1.53E-03	0.9428	4.29E-02	-8.91E-04	0.9687	4.26E-01	-2.04E-02	1
Seleno-methionine	Ratio to surrogate Methionine	7.36E-01	-1.86E-03	0.9542	7.54E-01	-5.83E-02	0.9911	8.36E-01	-1.87E-02	1
Serine	Ratio to Heavy	8.85E-02	8.53E-03	0.9978	No calibration possible			No calibration possible		
Serotonine	Ratio to surrogate Nicotinamide	3.51E-01	-2.43E-04	0.9812	6.75E-01	-1.34E-05	0.9631	6.64E-01	8.37E-01	1
Spermidine	No calibration possible									
Spermine	No calibration possible									
Succinate	Ratio to Heavy	3.46E-01	2.42E-02	0.9992	6.12E-01	3.19E+00	0.9906	7.01E-01	1.29E+00	1
Thiamine hydrochloride	No calibration possible									
Thymidine	Ratio to surrogate Pyruvic acid	3.31E+00	9.33E-03	0.9811	1.31E+00	1.98E-01	0.9282	2.01E+00	-1.79E-02	1
Thymidine 5'-monophosphate	Ratio to surrogate Hydroxyglutaric acid	1.24E-01	-3.00E-05	0.9963	1.16E-01	-8.02E-03	0.9196	6.19E-02	8.12E-04	1
Thymine	Ratio to surrogate Succinic acid	3.49E-01	-1.47E-03	0.9731	3.13E-01	1.05E-01	0.959	1.15E-01	1.27E-02	1
Trehalose	Ratio to Heavy	7.46E-01	-2.76E-03	0.9961	8.59E-01	1.06E+00	0.957	6.30E-01	2.66E-01	1
Tryptophan	Ratio to surrogate Valine	5.16E-01	-9.61E-06	0.9859	1.21E+00	1.10E+01	0.8534	No calibration possible		
TTP (Thymidinetriphosphate)	Ratio to surrogate ATP	5.48E-01	-1.62E-02	0.986	1.01E+00	-1.99E-01	0.9268	NaN	NaN	NaN
Tyrosine	Ratio to Heavy	1.78E+00	1.83E-02	0.9986	No calibration possible			No calibration possible		
UMP	Ratio to Heavy	1.01E+00	-1.61E-03	0.9974	1.17E+00	-2.56E-02	0.9887	1.18E+00	-1.66E-01	1
Uracil	Ratio to surrogate Hydroxyglutaric acid	1.96E-01	3.05E-03	0.9822	-9.54E+00	2.16E+00	-2193.68	1.40E-01	5.56E-03	1
Uridine	Ratio to surrogate Hydroxyglutaric acid	1.41E-01	8.86E-03	0.958	No calibration possible			7.01E-02	2.55E-01	1
Uridine 5'-diphosphate	Ratio to Heavy	1.65E+00	-8.23E-03	0.9927	2.64E+00	-4.62E-01	0.9815	2.55E+00	9.94E-01	1
Uridine 5'-triphosphate	Ratio to surrogate ATP	1.18E-01	-2.90E-02	0.98	2.07E-01	-8.44E-02	0.9264	No calibration possible		
Valine	Ratio to Heavy	3.08E-01	3.56E-03	0.9917	No calibration possible			No calibration possible		

Xanthine	Ratio to surrogate Hydroxyglutaric acid	4.78E-01	9.26E-03	0.9836	No calibration possible	2.58E-01	6.69E-01	1
Xylose	Ratio to surrogate Aspartic acid	1.03E-04	-8.02E-05	0.9633	No calibration possible	No calibration possible		

Table S19 Linear dynamic range of solvent standards and matrix-matched standards, recovery of solvent standards and extraction recovery of spiked matrix standards (urine and plasma) of the most abundant species for the xenobiotics and human estrogens measured on the reversed phase column. Not detected compounds are marked with n.d. (not detected) and bg indicates analytes where the background signal was too high to calculate analytical figures of merit

		Linear working range [ng/mL]			Recovery [%]		
**Compound**	**Species**	**Urine**	**Plasma**	**Solvent**	**Solvent**	**Urine**	**Plasma**
1-OH-pyrene	**$[M-H]^{-}$**, $[M+H]^{+}$	3 - 100	3 - 30	3 - 100	103±13	104±12	104±30
16-Epiestriol	**$[M-H]^{-}$**	1.5 - 500	0.5 - 500	0.5 - 500	99±11	96±6	114±4
16-Hydroxyestrone	**$[M-H]^{-}$**	1 - 100	1 - 100	0.1 - 100	105±18	96±30	103±10
17-Epiestriol	**$[M-H]^{-}$**	5 - 500	0.5 - 500	0.5 - 500	106±15	102±	106±8
2-Hydroxyestradiol	**$[M-H]^{-}$**	10 - 1000	100 - 1000	3 - 1000	112±15	99±8	n.d.
2-Methoxyestrone	**$[M-H]^{-}$**, $[M+H]^{+}$	3 - 300	0.9 - 300	0.9 - 300	111±5	97±9	104±12
2-Naphtol	**$[M-H]^{-}$**	5 - 500	0.5 - 500	0.5 - 500	108±5	80±3	65±2
2-Methoxyestradiol	**$[M-H]^{-}$**	3 - 300	3 - 300	0.3 - 300	113±11	101±13	101±9
2-tert-Butylphenol	**$[M-H]^{-}$**	2 - 2000	0.2 - 2000	0.2 - 2000	110±7	n.d.	6±5
3-Benzylidencampher	**$[M+H]^{+}$**	5 - 5000	15 - 1500	0.5 - 1500	83±6	70±2	21±6
4-Hydroxyestrone	**$[M-H]^{-}$**	0.5 - 500	5 - 500	0.5 - 500	109±10	97±6	7±9
4-Methoxyestradiol	**$[M-H]^{-}$**	1.5 - 50	1.5 - 50	0.2 - 50	104±7	91±9	80±7
4-Methoxyestrone	**$[M-H]^{-}$**, $[M+H]^{+}$	0.5 - 50	0.2 - 50	0.1 - 50	116±7	102±8	108±6
4-Methylbenzylidencampher	**$[M+H]^{+}$**	2 - 2000	2 - 2000	2 - 2000	88±3	93±5	58±15
4-octylphenol	**$[M-H]^{-}$**	1 - 1000	1 - 300	1 - 300	89±9	106±200	110±7
4-tert-octylphenol	**$[M-H]^{-}$**	1 - 1000	1 - 1000	1 - 1000	104±2	95±2	99±3
8-Prenylnaringenin	**$[M-H]^{-}$**, $[M+H]^{+}$	1 - 10	0.1 - 10	0.03 - 10	98±8	93±13	98±7
Aflatoxicol	**$[M+H]^{+}$**	5 - 50	0.5 - 50	0.01 - 50	92±7	111±10	110±5
Aflatoxin B1	**$[M+H]^{+}$**, $[M-H]^{-}$	0.1 - 100	0.1 - 30	0.01 - 100	95±11	102±14	113±33
Aflatoxin B2	**$[M+H]^{+}$**, $[M-H]^{-}$	0.1 - 100	0.1 - 30	0.01 - 100	98±7	111±18	114±5
Aflatoxin G1	**$[M+H]^{+}$**, $[M-H]^{-}$	0.1 - 100	0.1 - 30	0.01 - 100	99±9	93±15	102±88
Aflatoxin G2	**$[M+H]^{+}$**, $[M-H]^{-}$	1 - 100	0.1 - 30	0.01 - 100	97±5	107±14	101±10
Aflatoxin M1	**$[M+H]^{+}$**, $[M-H]^{-}$	1 - 100	0.1 - 100	0.01 - 100	98±4	99±9	96±10

Aflatoxin M2	**[M+H]**$^+$, [M-H]$^-$	1 - 100	1 - 30	0.3 - 100	113±7	104±9	105±15
Aflatoxin P1	**[M-H]**$^-$, [M+H]$^+$	0.1 - 100	10 - 100	0.01 - 100	107±7	95±22	91±8
Alpha-zearalanol	**[M-H]**$^-$, [M+H]$^+$	0.1 - 100	1 - 100	0.1 - 100	98±6	97±12	105±9
Alpha-zearalenol-14-glucuronide	**[M-H]**$^-$, [M+H]$^+$	10 - 100	0.1 - 100	3 - 100	120±26	85±27	n.d.
Alpha-zearalenol	**[M-H]**$^-$, [M+H]$^+$	0.1 - 100	0.3 - 100	0.1 - 100	102±5	100±7	97±8
Alternariol	**[M-H]**$^-$, [M+H]$^+$	1 - 100	0.1 - 100	0.01 - 100	117±22	99±2	101±2
Alternariol monomethylether	**[M-H]**$^-$, [M+H]$^+$	0.1 - 100	0.1 - 100	0.1 - 100	93±8	99±15	102±5
Anisodamine	**[M+H]**$^+$	3 - 100	3 - 100	0.1 - 100	88±11	104±8	84±2
Beauvericin	**[M+NH4**]$^+$	5 - 50	0.5 - 50	0.5 - 50	87±14	118±12	104±9
Benzophenone 1	**[M-H]**$^-$, [M+H]$^+$	0.1 - 10	0.1 - 10	0.01 - 10	114±15	108±5	86±3
Benzophenone 2	**[M-H]**$^-$	0.5 - 50	0.5 - 50	0.1 - 50	99±9	97±14	101±1
Benzylbutylphthalate	**[M+H]**$^+$	1.5 - 150	1.5 - 150	0.5 - 150	95±4	92±19	113±4
Benzylparaben	**[M-H]**$^-$	0.2 - 5	0.1 - 5	0.1- 5	104±16	100±8	99±8
Beta-zearalanol	**[M-H]**$^-$, [M+H]$^+$	0.3 - 100	0.1 - 100	0.1 - 100	122±4	96±7	78±
Beta-zearalenol-14-glucuronide	**[M-H]**$^-$, [M+H]$^+$	30 - 100	10 - 100	3 - 100	113±34	n.d.	110±7
Beta-zearalenol	**[M-H]**$^-$, [M+H]$^+$	0.3 - 100	0.3 - 30	0.1 - 100	123±7	99±7	106±8
Bisphenol A	**[M-H]**$^-$	1 - 100	1 - 100	1 - 100	103±3	105±7	106±3
Bisphenol AF	**[M-H]**$^-$	0.2 - 50	0.1 - 50	0.1 - 50	107±10	91±9	86±10
Bisphenol B	**[M-H]**$^-$	1 - 100	1 - 100	0.3 - 100	111±7	86±9	101±4
Bisphenol C	**[M-H]**$^-$	2 - 200	0.2 - 200	0.2 - 200	108±3	101±5	104±12
Bisphenol F	**[M-H]**$^-$	20 - 200	0.2 - 200	0.2 - 200	106±14	96±14	103±7
Bisphenol S	**[M-H]**$^-$	0.3 - 10	0.1 - 10	0.03 - 10	103±10	97±8	104±1
Butylparaben	**[M-H]**$^-$	0.1 - 50	0.1 - 50	0.1 - 50	101±7	106±2	1065±
Citrinin	**[M+H]**$^+$, [M-H]$^-$	10 - 100	1 - 100	0.1 - 30	91±7	40±7	96±4
Cotinine	**[M+H]**$^+$	3 - 100	3 - 100	1 - 100	107±7	98±8	Bg
Coumestrol	**[M-H]**$^-$, [M+H]$^+$	0.3 - 100	0.1 - 100	0.1 - 100	109±9	97±9	101±5
Daidzein	**[M-H]**$^-$, [M+H]$^+$	0.5 - 50	0.1 - 50	0.1 - 50	100±11	98±10	108±6
Deoxynivalenol	**[M-H]**$^-$, [M+H]$^+$	30 - 100	30 - 100	3 - 100	101±10	n.d.	n.d.
Dibutylphthalate	**[M+H]**$^+$	3 - 1000	3 - 1000	10 - 1000	100±4	105±8	55±8

E2-17-GlcA	**[M-H]**$^{-}$	15 - 150	15 - 150	1.5 - 150	103±20	97±18	77±18
Enterodiol	**[M-H]**$^{-}$	0.2 - 50	0.1 - 50	0.1 - 50	110±10	72±13	109±12
Enterolactone	**[M-H]**$^{-}$, [M+H]$^{+}$	0.3 - 100	0.3 - 100	0.1 - 100	117±7	106±1	100±2
Equol	**[M-H]**$^{-}$, [M+H]$^{+}$	3 - 100	0.3 - 100	0.1 - 100	117±21	102±16	110±7
Estradiol-3-sulfate	**[M-H]**$^{-}$	0.6 - 200	0.2 - 200	0.2 - 200	109±9	106±33	103±10
Estradiol	**[M-H]**$^{-}$	0.6 - 200	0.6 - 200	0.6 - 200	103±3	108±9	106±8
Estriol	**[M-H]**$^{-}$	2 - 200	2 - 200	0.2 - 200	114±8	95±14	104±9
Estrone	**[M-H]**$^{-}$	1 - 100	1 - 100	0.1 - 100	105±11	95±10	105±10
Ethinylestradiol	**[M-H]**$^{-}$	5 - 500	0.5 - 500	0.5 - 500	104±18	103±16	97±8
Ethylparaben	**[M-H]**$^{-}$	0.5 - 50	0.5 - 50	0.2 - 50	102±2	95±2	106±7
Fenarimol	**[M+H]**$^{+}$, [M-H]$^{-}$	0.5 - 5	0.5 - 5	0.02 - 5	102±9	n.d.	117±6
Formononetin	**[M+H]**$^{+}$, [M-H]$^{-}$	0.5 - 50	0.5 - 50	0.1 - 50	103±7	102±3	121±6
Fumonisin B1	**[M+H]**$^{+}$, [M-H]$^{-}$	3 - 100	3 - 100	1 - 100	94±17	85±21	79±19
Genistein	**[M-H]**$^{-}$, [M+H]$^{+}$	5 - 50	0.2 - 50	0.1 - 50	105±10	102±8	102±6
Glycitein	**[M-H]**$^{-}$, [M+H]$^{+}$	0.5 - 50	5 - 50	0.1 - 50	90±6	96±9	108±2
Isobutylparaben	**[M-H]**$^{-}$	0.5 - 50	0.1 - 50	0.1 - 50	106±7	96±4	104±9
Isoxanthohumol	**[M-H]**$^{-}$, [M+H]$^{+}$	0.5 - 5	0.5 - 5	0.1 - 5	98±6	99±12	104±6
Jacobine-N-oxide	**[M+H]**$^{+}$, [M-H]$^{-}$	10 -100	3 - 100	0.3 - 100	104±23	85±12	106±3
Jacobine	**[M+H]**$^{+}$	30 - 100	1 - 100	0.1 - 100	101±7	90±5	97±2
Matairesinol	**[M-H]**$^{-}$, [M+H]$^{+}$	6 - 200	2 - 200	0.2 - 200	104±2	85±15	103±4
MEHP	**[M-H]**$^{-}$, [M+H]$^{+}$	20 - 200	bg	0.6 - 200	98±3	96±16	bg
Methiocarb	**[M+H]**$^{+}$	9 - 300	0.3 - 300	0.3 - 300	109±9	107±8	92±10
Methylparaben	**[M-H]**$^{-}$	0.1 - 100	1 - 100	0.3 - 100	104±2	107±2	112±14
Mono_buty_phthalate	**[M-H]**$^{-}$, [M+H]$^{+}$	6 - 200	2 - 200	0.2 - 200	98±2	113±5	130±7
n-Butylbenzolsulfonamid	**[M-H]**$^{-}$, [M+H]$^{+}$	2 - 200	0.6 - 200	0.2 - 200	112±18	97±19	103±4
Nivalenol	**[M-H]**$^{-}$, [M+H]$^{+}$	-	30 - 100	3 - 100	105±14	n.d.	n.d.
Nonylphenol	**[M-H]**$^{-}$	0.5 - 500	1.5 - 500	0.5 - 500	102±7	103±8	104±13

Ochratoxin A	**[M-H]**$^{-}$, [M+H]$^{+}$	1 - 100	10 - 100	1 - 100	111±11	86±13	n.d.
Ochratoxin Alpha	**[M-H]**$^{-}$, [M+H]$^{+}$	3 - 100	1 - 30	1 - 100	107±13	18±7	47±7
Ochratoxin B	**[M-H]**$^{-}$, [M+H]$^{+}$	1 - 100	1 - 100	1 - 100	102±5	90±10	80±2
Octylmethoxycinnamate	**[M+H]**$^{+}$	50 - 5000	50 - 5000	5 - 5000	99±18	94±28	111±20
p-Hydrobenzoic_acid	**[M-H]**$^{-}$	9 - 3000	300 - 3000	3 - 3000	98±13	120±11	113±110
PFOA	**[M-H]**$^{-}$	0.5 - 50	0.5 - 50	0.2 - 50	100±7	94±2	90±44
PFOS	**[M-H]**$^{-}$	0.3 - 30	0.3 - 30	0.9 - 30	103±4	94±3	90±88
PhIP	**[M+H]**$^{+}$, [M-H]$^{-}$	0.1 - 100	0.3 - 30	0.01 - 100	109±13	97±1	98±2
Prochloraz	**[M+H]**$^{+}$	0.3 - 10	1 - 10	0.1 - 10	109±8	107±8	98±8
Propylparaben	**[M-H]**$^{-}$, [M+H]$^{+}$	0.1 - 50	0.2 - 50	0.01 - 50	104±2	112±1	110±2
Resveratrol	**[M-H]**$^{-}$, [M+H]$^{+}$	1.5 - 500	5 - 500	1.5 - 500	112±14	111±12	101±5
Riddeliin-N-oxide	**[M+H]**$^{+}$, [M-H]$^{-}$	2.4 - 800	0.8 - 800	0.8 - 800	88±30	97±10	96±14
Riddeliin	**[M+H]**$^{+}$, [M-H]$^{-}$	8 - 800	0.8 - 800	0.8 - 800	96±13	97±18	102±13
Scolpolamine	**[M+H]**$^{+}$, [M-H]$^{-}$	5 - 50	0.5 - 50	0.1 - 50	105±13	90±8	103±3
Sterigmatocystein	**[M+H]**$^{+}$, [M-H]$^{-}$	0.3 - 100	1 - 100	0.1 - 100	108±12	92±9	109±10
Tentoxin	**[M+H]**$^{+}$, [M-H]$^{-}$	3 - 100	1 - 100	0.1 - 100	95±10	111±5	108±9
Tetrabrombisphenol A	**[M-H]**$^{-}$	0.6 - 200	6 - 200	2 - 200	94±7	53±7	15±10
Toxin T2	**[M+H]**$^{+}$	3 - 100	3 - 100	1 - 100	92±9	72±10	77±6
trans-3-OH-cotinine	**[M+H]**$^{+}$	24 - 800	24 - 800	0.8 - 800	114±30	96±19	bg±17
Triclosan	**[M-H]**$^{-}$	0.3 - 100	0.3 - 100	0.1 - 100	100±8	81±25	106±10
Xanthohumol	**[M-H]**$^{-}$, [M+H]$^{+}$	0. 1 - 15	0.1 - 50	0.1 - 15	112±11	88±17	102±17
Zearalanone	**[M-H]**$^{-}$, [M+H]$^{+}$	0.1 - 100	0.1 - 100	0.1 - 100	106±9	102±7	105±5
Zearalenone-14-glucuronide	**[M-H]**$^{-}$	10 - 100	10 - 100	1 - 100	104±22	n.d	106±17
Zearalenone-14-sulfate	**[M-H]**$^{-}$	1 - 100	0.1 - 100	0.3 - 100	109±111	83±7	91±8
Zearalenone	**[M-H]**$^{-}$, [M+H]$^{+}$	0.3 - 100	0.3 - 100	0.1 - 100	101±2	110±2	103±3

Table S20 Linear dynamic range of solvent standards and matrix-matched standards, recovery of solvent standards and extraction recovery of spiked matrix standards (urine and plasma) of the most abundant species for endogenous metabolites on the HILIC column. Not detected compounds are marked with n.d. (not detected) and 'bg' indicates analytes where the background signal was too high to calculate analytical figures of merit

Compound	Species	Linear dynamic range [µM]			Recovery [%]		
		Solvent	Urine	Plasma	Solvent	Urine	Plasma
1-Methylhydantoin	**[M-H]⁻**	1 - 10	**bg**	3 - 10	99±4	bg	bg
1-Methylnicotinamide		Not detected					
2-(Carbamoylamino)butanedioic acid	**[M-H]⁻**, [M+H]⁺	0.1 - 10	1 - 10	0.1 - 3	98±18	94±6	33±4
2'-Deoxyadenosine 5'-monophosphate	**[M+H]⁺**, [M-H]⁻	0.01 - 10	0.3 - 10	0.1 - 10	115±7	109±12	110±19
2'-Deoxycytidine	**[M-H]⁻**, [M+H]⁺	0.01 - 3	0.3 - 10	0.3 - 10	91±7	42±5	107±9
2-Deoxycytidine 5'-Monophosphate	**[M-H]⁻**, [M+H]⁺	0.01 - 10	0.1 - 10	0.3 - 10	104±9	57±8	n.d.
2'-Deoxyuridine	**[M-H]⁻**, [M+H]⁺	0.001 - 10	0.03 - 10	0.03 - 10	108±5	50±9	38±7
2-Phosphoglyceric acid + 3-Phosphoglyceric acid	**[M-H]⁻**, [M+H]⁺	0.06 - 20	0.2 - 20	0.6 - 20	118±13	92±14	n.d.
3'AMP	**[M+H]⁺**, [M-H]⁻	0.01 - 10	3 - 10	0.03 - 10	98±4	n.d.	n.d.
3-Methyl-2-oxovaleric acid	**[M-H]⁻**	0.03 - 10	0.3 - 10	bg	100±1	117±16	bg
3-Methylcytidine	**[M-H]⁻**, [M+H]⁺	0.3 - 10	bg	0.3 - 10	89±18	bg	bg
4-Hydroxy-proline	**[M-H]⁻**, [M+H]⁺	0.03 - 10	1 - 10	bg	98±7	bg	114±12
5'AMP	**[M+H]⁺**, [M-H]⁻	0.01 - 10	0.3 - 10	0.03 - 10	95±6	102±4	112±10
5'-Deoxy-5'-Methylthioadenosine	**[M+H]⁺**, [M-H]⁻	0.01 - 10	0.01 - 10	0.01 - 10	101±1	116±25	105±15
5-Methyluridine	**[M-H]⁻**, [M+H]⁺	0.01 - 10	0.1 - 10	0.3 - 10	103±5	100±5	74±4
6-Phosphogluconate	**[M-H]⁻**, [M+H]⁺	0.1 - 10	0.3 - 10	0.3 - 10	107±7	n.d.	n.d.
Adenine	**[M+H]⁺**, [M-H]⁻	0.1 - 10	0.3 - 10	0.1 - 10	98±2	116±12	129±1
Adenosine	**[M+H]⁺**, [M-H]⁻	0.01 - 10	0.1 - 10	0.01 - 10	101±1	92±20	92±3
Adenosine 3',5'-cyclic monophosphate	**[M-H]⁻**, [M+H]⁺	0.01 - 10	0.3 - 10	0.1 - 10	103±7	100±4	70±2
Adenosine 5'-triphosphate	**[M+H]⁺**, [M-H]⁻	0.3 - 10	0.3 - 10	n.d.	113±13	n.d.	n.d.
Adenosine diphosphate	**[M-H]⁻**, [M+H]⁺	0.3 - 10	0.1 - 10	0.3 - 10	108±10	121±15	72±14
Alanine	**[M+H]⁺**, [M-H]⁻	0.3 - 10	bg	bg	101±4	bg	bg
alpha-Aminoadipic acid	**[M+H]⁺**, [M-H]⁻	0.03 - 10	bg	bg	107±9	bg	bg
alpha-Ketoglutarate	**[M-H]⁻**	0.03 - 10	bg	bg	99±3	bg	bg
Arginine	**[M-H]⁻**, [M+H]⁺	No calibration possible					
Argininosuccinic acid	**[M+H]⁺**,	0.01 - 10	0.3 - 10	0.03 - 10	102±1	107±2	105±6

Compound	Species	Linear dynamic range [µM]			Recovery [%]		
		Solvent	Urine	Plasma	Solvent	Urine	Plasma
	$[M-H]^-$						
Asparagine	**$[M-H]^-$**, $[M+H]^+$	0.03 - 10	3 - 10	0.3 - 10	101±1	bg	bg
Aspartate	**$[M-H]^-$**, $[M+H]^+$	0.03 -10	1 - 10	0.3 -10	97±8	92±16	114±5
Betaine	**$[M+H]^+$**, $[M-H]^-$	0.03 - 10	bg	bg	107±8	bg	bg
Biotin	**$[M+H]^+$**, $[M-H]^-$	0.01 - 10	0.3 - 10	0.1 - 10	88±14	97±22	21±2
Carnitine	**$[M+H]^+$**	0.03 - 10	bg	bg	95±13	bg	bg
Choline chloride			Not detected				
cis-Aconitate	**$[M-H]^-$**	0.1 - 10	bg	bg	95±1	bg	bg
Citrate + Isocitrate	**$[M-H]^-$**	0.6 - 20	bg	0.2 - 20	92±10	bg	bg
CMP	**$[M-H]^-$**, $[M+H]^+$	0.1 - 10	0.3 - 10	0.3 - 10	95±10	74±5	51±6
Cysteic acid	**$[M-H]^-$**, $[M+H]^+$	0.01 - 10	0.03 - 10	0.1 -10	101±10	91±10	40±2
Cysteine	**$[M-H]^-$**, $[M+H]^+$	1 - 10	n.d.	n.d.	97±14	n.d.	n.d.
Cysteinyl-glycine	**$[M-H]^-$**, $[M+H]^+$	1 – 10	n.d	n.d	n.d.	n.d.	n.d.
Cystine	**$[M+H]^+$**, $[M-H]^-$	0.1 - 10	bg	bg	94±1	bg	bg
Cytidine	**$[M+H]^+$**, $[M-H]^-$	0.03 - 10	0.03 - 10	0.01 - 10	91±6	77±5	108±14
Cytidine 5'-triphosphate	**$[M+H]^+$**, $[M-H]^-$	1 - 10	3 - 10	n.d.	114±10	n.d.	n.d.
Cytosine	**$[M+H]^+$**, $[M-H]^-$	0.01 - 10	1 - 10	0.1 10	107±5	50±12	133±35
Deoxyguanosine triphosphate	**$[M+H]^+$**, $[M-H]^-$	0.3 -10	1 - 10	n.d	113±13	n.d.	n.d.
Dihydroxyacetonephosphate	**$[M-H]^-$**, $[M+H]^+$	1 - 10	3 - 10	3 -10	91±19	n.d.	n.d.
Dihydroxyisovalerate	**$[M-H]^-$**	0.03 - 10	0.1 -10	0.3 - 10	104±16	97±22	110±6
Erythritol	**$[M-H]^-$**	0.03 - 10	bg	1 - 10	95±5	bg	91±8
Erythrose-4-phosphate	**$[M-H]^-$**	0.3 - 10	3 - 10	3 - 10	95±23	n.d.	n.d.
Flavinadenin dinucleotide	**$[M+H]^+$**, $[M-H]^-$	0.1 - 10	1 - 10	1 - 10	102±3	66±8	14±4
Hexose (Frucose, Galactose, Mannose, Glucose, Inositol)	**$[M-H]^-$**	0.5 - 5	bg	bg	113±3	bg	bg
Fructose-1,6-bisphosphate	**$[M-H]^-$**, $[M+H]^+$	0.3 - 10	1 - 10	n.d.	108±19	105±15	n.d.
Hexose-6-phosphate (Fructose-6-phosphate, Glucose-1-phosphate, Glucose-6-phosphate)	**$[M-H]^-$**, $[M+H]^+$	0.03 - 30	0.09 - 30	0.3 - 30	95±10	n.d.	n.d.
Fumarate	**$[M-H]^-$**	0.1 - 10	3 - 10	0.3 -10	98±4	93±7	134±4
Gluconate	**$[M-H]^-$**	0.1 - 10	bg	1 - 10	106±18	bg	93±5
Glutamate	**$[M-H]^-$**, $[M+H]^+$	0.01 -10	0.3 - 10	bg	100±2	106±27	bg
Glutamine	**$[M+H]^+$**, $[M-H]^-$	0.03 -10	bg	bg	98±1	bg	bg
Glutamyl-cysteine	**$[M+H]^+$**, $[M-H]^-$	0.1 -3	n.d.	n.d.	112±21	n.d.	n.d.
Glutathione, oxidized	**$[M+H]^+$**, $[M-H]^-$	0.1 - 10	1 - 10	0.3 - 10	95±3	27±15	n.d.

Compound	Species	Linear dynamic range [µM]			Recovery [%]		
		Solvent	Urine	Plasma	Solvent	Urine	Plasma
Glutathione, reduced	**[M-H]⁻**, [M+H]⁺	0.1 - 10	0.1 - 10	1 - 10	n.d.	n.d.	n.d.
Glycine	**[M-H]⁻**, [M+H]⁺	0.3 - 10	bg	bg	102±7	Bg	bg
Glyoxylic acid		Not detected					
GMP	**[M-H]⁻**, [M+H]⁺	0.01 - 10	0.3 - 10	0.1 - 10	102±4	123±6	119±27
Guanidineacetic acid	**[M+H]⁺**, [M-H]⁻	0.3 - 10	bg	1 -10	104±16	bg	93±9
Guanine	**[M+H]⁺**, [M-H]⁻	0.01 - 10	0.03 - 10	0.03 - 10	93±10	108±13	51±4
Guanosine + Isoguanosine	**[M+H]⁺**, [M-H]⁻	0.2 - 6	0.6 - 20	0.02 - 6	99±12	32±3	101±2
Guanosine 3',5'-cyclic monophosphate	**[M+H]⁺**, [M-H]⁻	0.01 - 10	0.1 - 10	1 - 10	107±8	71±11	32±8
Guanosine 5'-diphosphate	**[M-H]⁻**, [M+H]⁺	0.3 - 10	0.3 - 10	3 - 10	107±17	71±26	n.d.
Guanosine 5'-triphosphate	**[M+H]⁺**, [M-H]⁻	0.3 - 10	3 - 10	n.d.	108±21	n.d.	n.d.
Histidine	**[M+H]⁺**, [M-H]⁻	0.3 - 10	bg	bg	98±3	bg	bg
Homocysteine	**[M+H]⁺**, [M-H]⁻	0.3 - 10	0.03 - 10	1 - 10	84±9	n.d.	n.d.
Homoserine + Threonine	**[M+H]⁺**, [M-H]⁻	0.03 - 10	bg	bg	102±2	bg	bg
Hydroxyglutaric acid	**[M-H]⁻**	0.01 -10	bg	1 - 10	103±3	bg	115±5
Inosine	**[M-H]⁻**, [M+H]⁺	0.01 - 10	0.3 - 10	0.03 - 10	101±5	74±6	115±9
Inosine 5'-monophosphate	**[M-H]⁻**, [M+H]⁺	0.1 - 10	0.1 - 10	0.3 - 10	47±7	70±8	47±4
Isoleucine + Leucine	**[M-H]⁻**, [M+H]⁺	0.06 - 20	bg	bg	Bg	bg	bg
Ketoisovalerate	**[M-H]⁻**	0.1 - 10	0.3 - 10	3 -10	94±5	bg	94±9
Kynurenine	**[M+H]⁺**, [M-H]⁻	0.01 - 10	0.3 - 10	0.03 - 10	24±5	54±13	24±11
Lactate	**[M-H]⁻**	0.3 - 10	1 - 10	1 - 10	Bg	bg	bg
L-Citrulline	**[M+H]⁺**, [M-H]⁻	0.03 - 10	bg	3 - 10	bg	bg	bg
L-Cystathionine	**[M+H]⁺**, [M-H]⁻	0.03 - 10	1 - 10	0.03 - 10	94±1	107±3	94±13
L-Ornithine	**[M-H]⁻**, [M+H]⁺	0.1 - 10	0.3 -10	bg	104±5	96±11	bg
Lysine	**[M+H]⁺**, [M-H]⁻	0.3 - 10	bg	bg	Bg	bg	bg
Malate	**[M-H]⁻**	0.01 - 10	0.1 - 10	0.03 – 10	111±1	115±1	111±4
Mannitol	**[M-H]⁻**	0.03 -10	bg	bg	Bg	bg	bg
Mannitol 1-phosphate	**[M-H]⁻**, [M+H]⁺	0.1 - 10	1 - 10	0.3 - 10	68±9	n.d.	68±12
Melatonine	**[M+H]⁺**, [M-H]⁻	0.001 - 10	0.03 - 10	0.1 - 10	119±15	115±9	119±3
Methionine	**[M+H]⁺**, [M-H]⁻	0.03 - 10	0.3 - 10	0.1 - 10	110±1	bg	110±4
Methionine sulfone	**[M+H]⁺**, [M-H]⁻	0.01 - 10	bg	bg	100±28	bg	bg
Mevalonic acid	**[M-H]⁻**	0.01 -10	1 - 10	0.3 - 10	124±11	101±10	124±7

Compound	Species	Linear dynamic range [µM]			Recovery [%]		
		Solvent	Urine	Plasma	Solvent	Urine	Plasma
N4-Acetylcytidine	**[M+H]⁺**, [M-H]⁻	0.01 - 10	0.3 - 10	1 - 10	100±4	95±17	100±9
N-Acetyl-Asp-Glu	**[M-H]⁻**, [M+H]⁺	0.01 - 10	0.3 - 10	bg	93±5	61±5	bg
N-Acetyl-L-aspartic acid	**[M-H]⁻**, [M+H]⁺	0.03 – 10	bg	0.1 -10	96±4	bg	96±3
N-Acetyl-serine	**[M-H]⁻**, [M+H]⁺	0.03 - 10	0.3 - 10	0.3 - 10	92±4	99±6	92±12
NAD+	**[M+H]⁺**, [M-H]⁻	0.01 - 10	0.3 - 10	0.3 - 10	111±2	97±20	111±21
NADH	**[M+H]⁺**, [M-H]⁻	0.1 - 10	n.d.	n.d.	n.d.	n.d.	n.d.
NADP+	**[M+H]⁺**, [M-H]⁻	0.1 - 10	1 - 10	1 - 10	n.d.	n.d.	n.d.
NADPH	**[M+H]⁺**, [M-H]⁻	1 - 10	3 - 10	3 - 10	n.d.	n.d.	n.d.
Nicotinamide	**[M+H]⁺**, [M-H]⁻	0.01 - 10	0.01 - 10	0.01 - 10	105±1	90±5	105±3
Octopamine		Not detected					
Oxaloacetic acid	**[M-H]⁻**	1 - 10	n.d.	n.d.	97±18	n.d.	n.d.
Palmitic acid	**[M-H]⁻**, [M+H]⁺	n.d.	n.d.	n.d.	n.d.	n.d.	n.d.
Phenylalanine	**[M-H]⁻**, [M+H]⁺	0.03 - 10	0.3 - 10	bg	99±1	103±9	Bg
Phosphocreatine	**[M+H]⁺**, [M-H]⁻	0.1 - 10	1 - 10	0.1 10	110±9	103±12	n.d.
Proline	**[M+H]⁺**, [M-H]⁻	0.03 - 10	1 - 10	bg	102±1	115±12	Bg
Propionyl-L-carnitine	**[M+H]⁺**, [M-H]⁻	0.01 - 10	1 - 10	0.3 - 10	100±8	121±16	99±18
Pseudouridine	**[M-H]⁻**, [M+H]⁺	0.01 - 10	0.1 - 10	0.1 - 10	102±2	100±23	111±10
Pyruvate	**[M-H]⁻**	0.1 - 10	bg	1 - 10	101±4	bg	Bg
Ribose	**[M-H]⁻**	1 - 10	n.d.	n.d.	105±6	n.d.	n.d.
Ribose-5-phosphate + Ribulose-5-phosphate	**[M-H]⁻**, [M+H]⁺	0.06 - 20	0.6 - 20	0.6 - 20	98±8	26±5	10±3
S-(Adenosyl)-methionine	**[M+H]⁺**, [M-H]⁻	0.1 - 10	1 - 10	n.d.	119±6	n.d.	n.d
Sarcosine	**[M+H]⁺**, [M-H]⁻	0.1 - 10	1 - 10	0.1 - 10	102±22	96±9	102±11
Sedoheptulose-7-phosphate	**[M-H]⁻**, [M+H]⁺	0.01 - 10	0.1 - 10	0.1 - 10	93±16	112±33	n.d.
Seleno-methionine	**[M+H]⁺**, [M-H]⁻	0.03 - 10	0.3 - 10	0.1 - 10	102±3	107±8	114±5
Serine	**[M-H]⁻**, [M+H]⁺	0.1 - 10	bg	bg	100±7	Bg	Bg
Serotonine	**[M+H]⁺**, [M-H]⁻	0.03 - 10	0.03 - 10	0.03 - 10	98±3	114±17	99±5
Spermidine		Not detected					
Spermine		Not detected					
Succinate	**[M-H]⁻**	0.1 - 10	1 - 10	0.1 - 10	98±2	115±2	151±6
Thiamine hydrochloride		Not detected					
Thymidine	**[M-H]⁻**, [M+H]⁺	0.01 - 10	0.1 - 10	0.03 -10	99±2	57±13	55±8
Thymidine 5'-monophosphate	**[M-H]⁻**, [M+H]⁺	0.01 - 10	0.1 - 10	0.03 - 10	109±7	60±4	33±2

Compound	Species	Linear dynamic range [µM]			Recovery [%]		
		Solvent	Urine	Plasma	Solvent	Urine	Plasma
Thymine	**[M-H]**$^-$, [M+H]$^+$	0.03 - 10	0.3 - 10	0.1 - 10	99±7	98±15	45±4
Trehalose	**[M-H]**$^-$, [M+H]$^+$	0.01 - 10	0.3 - 10	0.1 - 10	98±1	95±12	94±6
Tryptophan	**[M-H]**$^-$, [M+H]$^+$	0.01 - 10	bg	bg	93±3	Bg	Bg
TTP (Thymidinetriphosphate)	**[M-H]**$^-$, [M+H]$^+$	0.3 - 10	0.3 – 10	n.d.	110±8	106±6	n.d.
Tyrosine	**[M-H]**$^-$, [M+H]$^+$	0.03 - 10	bg	bg	98±3	Bg	Bg
UMP	**[M-H]**$^-$, [M+H]$^+$	0.01 - 10	1 - 10	0.3 - 10	98±7	114±9	n.d.
Uracil	**[M-H]**$^-$, [M+H]$^+$	0.1 - 10	bg	0.03 - 10	99±3	Bg	68±5
Uridine	**[M-H]**$^-$, [M+H]$^+$	0.01 - 10	bg	1 - 10	105±5	Bg	111±4
Uridine 5'-diphosphate	**[M-H]**$^-$, [M+H]$^+$	0.3 - 10	0.3 - 10	1 - 10	105±8	95±7	n.d.
Uridine 5'-triphosphate	**[M-H]**$^-$, [M+H]$^+$	1 - 10	3 – 10	n.d.	109±15	n.d.	n.d.
Valine	**[M-H]**$^-$, [M+H]$^+$	0.03 – 10	bg	bg	97±3	bg	bg
Xanthine	**[M-H]**$^-$, [M+H]$^+$	0.01 - 10	bg	0.3 - 10	103±6	bg	90±2
Xylose	**[M-H]**$^-$	1 - 10	n.d.	n.d.	91±16	n.d.	n.d.

Table S21 Limit of detection (LOD), retention time and the detected adducts with the most abundant one in bold in solvent and two matrices (urine and plasma) for endogenous metabolites on the HILIC column in ng/mL. Not detected compounds are marked with n.d. (not detected) and bg indicates analytes where the background signal was too high for LOD determination

Compound	Neutral exact mass [m/z]	Species	Retention time [min]			LOD [ng/mL]		
			Solvent	Urine	Plasma	Solvent	Urine	Plasma
1-Methylhydantoin	114.0429	**[M-H]$^-$**	5.2	5.2	5.2	10	bg	342
1-Methylnicotinamide	136.0637	Not detected						
2-(Carbamoylamino)butanedioic acid	176.0433	**[M-H]$^-$**, [M+H]$^+$	3.9	4.3	4.9	35	176	53
2’-Deoxyadenosine 5’-monophosphate	331.0682	**[M+H]$^+$**, [M-H]$^-$	2	2.1	2.6	0.99	132	17
2'-Deoxycytidine	227.0906	**[M-H]$^-$**, [M+H]$^+$	2.7	2.7	2.7	0.91	68.1	91
2-Deoxycytidine 5'-Monophosphate	307.0569	**[M-H]$^-$**, [M+H]$^+$	3.3	4	4.6	3.1	92	92
2'-Deoxyuridine	228.0746	**[M-H]$^-$**, [M+H]$^+$	2	2	2	2.3	6.8	6.8
2-Phosphoglyceric acid + 3-Phosphoglyceric acid	185.9929	**[M-H]$^-$**, [M+H]$^+$		8.0-8.7		5.6	56	56
3'AMP	347.0631	**[M+H]$^+$**, [M-H]$^-$	2.1	2.2	4.2*	3.5	1041	104
3-Methyl-2-oxovaleric acid	130.0630	**[M-H]$^-$**	1.1	1.2	1.1	2.6	10	bg
3-Methylcytidine	257.1012	**[M-H]$^-$**, [M+H]$^+$	2.4	2.2	2.1	257	bg	257
4-Hydroxy-proline	131.0582	**[M-H]$^-$**, [M+H]$^+$	4.4	4.4	4.3	6.6	26.2	6.6
5'AMP	347.0631	**[M+H]$^+$**, [M-H]$^-$	2.5	2.6	4.2*	3.5	104	10.4
5'-Deoxy-5'-Methylthioadenosine	297.0896	**[M+H]$^+$**, [M-H]$^-$	1.6	1.6	1.6	1.2	1.2	1.5
5-Methyluridine	258.0852	**[M-H]$^-$**, [M+H]$^+$	2	2	2	1.0	10	13
6-Phosphogluconate	276.0246	**[M-H]$^-$**, [M+H]$^+$	8.5-8.7	8-8.4	7.5-8.2	28	83	83
Adenine	135.0545	**[M+H]$^+$**, [M-H]$^-$	2.3	2.3	2.3	0.54	1.4	0.54
Adenosine	267.0968	**[M+H]$^+$**, [M-H]$^-$	2.1	2.2	2.1	1.1	2.7	1.6
Adenosine 3',5'-cyclic monophosphate	329.0525	**[M-H]$^-$**, [M+H]$^+$	1.9	1.9	1.9	1.3	99	16
Adenosine 5'-triphosphate	506.9957	**[M+H]$^+$**, [M-H]$^-$	9.0-9.4	8.9-9.1	n.d.	152	152	bg
Adenosine diphosphate	427.0294	**[M-H]$^-$**, [M+H]$^+$	8.7-9	8.5-8.7	8.3-8.6	128	214	128
Alanine	89.0477	**[M+H]$^+$**, [M-H]$^-$	4.6	4.5	4.7	36	bg	bg
alpha-Aminoadipic acid	161.0688	**[M+H]$^+$**, [M-H]$^-$	4	4	4.8	6.4	bg	bg
alpha-Ketoglutarate	146.0215	**[M-H]$^-$**	2.8	2.9	3.3	5.8	bg	bg

Compound	Neutral exact mass [m/z]	Species	Retention time [min]			LOD [ng/mL]		
			Solvent	Urine	Plasma	Solvent	Urine	Plasma
Arginine	174.1117	**[M-H]$^-$**, [M+H]$^+$	No calibration possible					
Argininosuccinic acid	290.1226	**[M+H]$^+$**, [M-H]$^-$	5	5.2	5.2	1.2	58	5.8
Asparagine	132.0535	**[M-H]$^-$**, [M+H]$^+$	5.1	5.2	5.2	5.3	bg	26
Aspartate	133.0375	**[M-H]$^-$**, [M+H]$^+$	4.1	4.2	4.8	5.3	39.91125	53
Betaine	117.0790	**[M+H]$^+$**, [M-H]$^-$	2.8	2.7	2.8	4.7	bg	bg
Biotin	244.0882	**[M+H]$^+$**, [M-H]$^-$	1.5	1.4	1.3	0.49	7.3	4.9
Carnitine	161.1052	**[M+H]$^+$**	4.1	4.1	4	4.8	bg	bg
Choline chloride	104.1075		Not detected					
cis-Aconitate	174.0164	**[M-H]$^-$**	3.2-3.4	3.3-3.5	5-5.1	7.0	bg	bg
Citrate + Isocitrate	192.0270	**[M-H]$^-$**	8.0-8.9	4.9-5	7.2-8.2**	192	bg	192.027
CMP	323.0519	**[M-H]$^-$**, [M+H]$^+$	4.2	4.2	4.8	13	97	65
Cysteic acid	169.0045	**[M-H]$^-$**, [M+H]$^+$	3.9-4.1	4.3	4.8	1.7	5.1	17
Cysteine	121.0197	**[M-H]$^-$**, [M+H]$^+$	6.2	n.d.	n.d.	121	bg	bg
Cysteinyl-glycine	178.0412	**[M-H]$^-$**, [M+H]$^+$	5.7	5.7	5.7	1068	bg	bg
Cystine	240.0238	**[M+H]$^+$**, [M-H]$^-$	6.4	6.4	6.4	96	bg	bg
Cytidine	243.0855	**[M+H]$^+$**, [M-H]$^-$	3.4	3.3	3.3	7.3	7.3	24
Cytidine 5'-triphosphate	482.9845	**[M+H]$^+$**, [M-H]$^-$	8.7	8.8	n.d.	193	966	bg
Cytosine	111.0433	**[M+H]$^+$**, [M-H]$^-$	3.4	3.3	3.3	1.1	111	11
Deoxyguanosine triphosphate	506.9957	**[M+H]$^+$**, [M-H]$^-$	9.1	9.1	n.d	152	507	bg
Dihydroxyacetonephosphate	169.9980	**[M-H]$^-$**, [M+H]$^+$	3.6	3.9	4.8	68	68	425
Dihydroxyisovalerate	134.0579	**[M-H]$^-$**	1.3	1.4	1.3	4.0	4.0	4.0
Erythritol	122.0579	**[M-H]$^-$**	3.1	3.1	3.1	4.9	bg	49
Erythrose-4-phosphate	200.0086	**[M-H]$^-$**	3.6-4.6	4-4.9	5.1-5.2	80	bg	340
Flavinadenin dinucleotide	785.1571	**[M+H]$^+$**, [M-H]$^-$	1.8	2.2	2.2	314	314	393
Hexose (Frucose, Galactose, Mannose, Glucose, Inositol)	180.0634	**[M-H]$^-$**	5.5	5.4	5.5	90	bg	bg
Fructose-1,6-bisphosphate	339.9961	**[M-H]$^-$**, [M+H]$^+$	9	8.6	n.d.	102	340	bg
Hexose-6-phosphate (Fructose-6-phosphate, Glucose-1-phosphate, Glucose-6-phosphate)	260.0297	**[M-H]$^-$**, [M+H]$^+$	4.5-5	4.9-5	5.1-5.2	10	2.6	10
Fumarate	116.0110	**[M-H]$^-$**	4.3	4.4	5	46	70	4.6
Gluconate	196.0583	**[M-H]$^-$**	3.1-3.4	4	4.7	7.8	bg	98
Glutamate	147.0532	**[M-H]$^-$**, [M+H]$^+$	4	4.1	4.8	59	59	bg

Compound	Neutral exact mass [m/z]	Species	Retention time [min]			LOD [ng/mL]		
			Solvent	Urine	Plasma	Solvent	Urine	Plasma
Glutamine	146.0691	**[M+H]⁺**, [M-H]⁻	4.9	4.9	4.9	5.8	bg	bg
Glutamyl-cysteine	250.0623	**[M+H]⁺**, [M-H]⁻	3.1	3.2	5.5	100	bg	bg
Glutathione, oxidized	612.1519	**[M+H]⁺**, [M-H]⁻	5.3	5.3	5.5	245	612	306
Glutathione, reduced	307.0838	**[M-H]⁻**, [M+H]⁺	3.1-3.7	3.3-4	5.4	154	154	307
Glycine	75.0320	**[M-H]⁻**, [M+H]⁺	5.1	5.1	5.1	30	bg	bg
Glyoxylic acid	74.0004		Not detected					
GMP	363.0580	**[M-H]⁻**, [M+H]⁺	4.7	5	5.1	15	145	18
Guanidineacetic acid	117.0538	**[M+H]⁺**, [M-H]⁻	4.8	5	5	47	bg	47
Guanine	151.0494	**[M+H]⁺**, [M-H]⁻	3.5	3.5	3.4	0.60	1.5	1.5
Guanosine + Isoguanosine	283.0917	**[M+H]⁺**, [M-H]⁻	3.6-3.8	3.6-3.8	3.6-3.8	2.8	85	2.8
Guanosine 3',5'-cyclic monophosphate	345.0474	**[M+H]⁺**, [M-H]⁻	2.6	2.6	2.6	1.4	104	173
Guanosine 5'-diphosphate	443.0243	**[M-H]⁻**, [M+H]⁺	8.8-9.0	8.5-8.8	8.8	133	133	13291
Guanosine 5'-triphosphate	522.9907	**[M+H]⁺**, [M-H]⁻	8.7	8.7	n.d.	209	837	bg
Histidine	155.0695	**[M+H]⁺**, [M-H]⁻	7.6	6.9	7.1	7.8	bg	bg
Homocysteine	135.0354	**[M+H]⁺**, [M-H]⁻	4.2-5.9	4.2.59	4.3-5.8	41	41	135
Homoserine + Threonine	119.0582	**[M+H]⁺**, [M-H]⁻	4.8-5	4.8-5	4.7-5	4.8	bg	bg
Hydroxyglutaric acid	148.0372	**[M-H]⁻**	2.6	2.7	3.5	5.9	bg	59
Inosine	268.0808	**[M-H]⁻**, [M+H]⁺	2.8	2.7	2.7	2.7	80	8.0
Inosine 5'-monophosphate	348.0471	**[M-H]⁻**, [M+H]⁺	3.8	4.1	4.8	10	104	139
Isoleucine + Leucine	131.0946	**[M-H]⁻**, [M+H]⁺	2.8-3	2.6-2.7	2.8-2.9	5.2	bg	bg
Ketoisovalerate	116.0473	**[M-H]⁻**	1.2	1.3	1.2	2.3	93	35
Kynurenine	208.0848	**[M+H]⁺**, [M-H]⁻	3	3	2.9	0.83	42	6.2
Lactate	90.0317	**[M-H]⁻**	1.7	1.7	1.7	45	54	180
L-Citrulline	175.0957	**[M+H]⁺**, [M-H]⁻	5.2	5.2	5.2	70	bg	525
L-Cystathionine	222.0674	**[M+H]⁺**, [M-H]⁻	6.3	6.3	6.3	8.9	8.9	0.89
L-Ornithine	132.0899	**[M-H]⁻**, [M+H]⁺	5.2	5.2	5.2	0.92	48	bg
Lysine	146.1055	**[M+H]⁺**, [M-H]⁻	12.6	12.6	12.1	146	bg	bg
Malate	134.0215	**[M-H]⁻**	3.2	3.4	4.6	0.40	9.4	8.0
Mannitol	182.0790	**[M-H]⁻**	4.1	4.1	4.1	73	bg	bg
Mannitol 1-phosphate	262.0454	**[M-H]⁻**, [M+H]⁺	4.5	4.9	5.1	10	92	105

Compound	Neutral exact mass [m/z]	Species	Retention time [min]			LOD [ng/mL]		
			Solvent	Urine	Plasma	Solvent	Urine	Plasma
Melatonine	232.1212	**$[M+H]^+$**, $[M-H]^-$	1.4	1.3	1.3	0.23	7.0	23
Methionine	149.0510	**$[M+H]^+$**, $[M-H]^-$	3.3	3.3	3.3	0.89	21	bg
Methionine sulfone	181.0409	**$[M+H]^+$**, $[M-H]^-$	4.2	4.3	4.2	1.8	bg	bg
Mevalonic acid	148.0736	**$[M-H]^-$**	1.3	1.3	1.3	1.5	148	44
N4-Acetylcytidine	285.0961	**$[M+H]^+$**, $[M-H]^-$	2	1.9	1.9	1.1	11	143
N-Acetyl-Asp-Glu	304.0906	**$[M-H]^-$**, $[M+H]^+$	4.7	4.7	5.2	12	304	bg
N-Acetyl-L-aspartic acid	175.0482	**$[M-H]^-$**, $[M+H]^+$	2.5	2.6	2.8	0.70	bg	14
N-Acetyl-serine	147.0532	**$[M-H]^-$**, $[M+H]^+$	1.9	1.9	2	0.44	44	7.4
NAD+	663.1091	**$[M+H]^+$**, $[M-H]^-$	3.5	4	4.6	27	265	265
NADH	665.1248	**$[M+H]^+$**, $[M-H]^-$	3.1	n.d.	n.d.	67	bg	bg
NADP+	743.0755	**$[M+H]^+$**, $[M-H]^-$	6.3-6.9	6.3-6.9	6.7-7.2	74	743	743
NADPH	745.0911	**$[M+H]^+$**, $[M-H]^-$	7-7.5	6.6-7.2	7.4	745	2235	2235
Nicotinamide	122.0480	**$[M+H]^+$**, $[M-H]^-$	1.9	1.9	1.9	1.2	1.2	1.2
Octopamine	153.0790	Not detected						
Oxaloacetic acid	132.0059	**$[M-H]^-$**	5.9	n.d.	n.d.	396.0	bg	bg
Palmitic acid	256.2402	**$[M-H]^-$**, $[M+H]^+$	1.1	1.1	1.1	bg	bg	bg
Phenylalanine	165.0790	**$[M-H]^-$**, $[M+H]^+$	2.7	2.7	2.7	0.66	0.33	bg
Phosphocreatine	211.0358	**$[M+H]^+$**, $[M-H]^-$	4.3	4.3	5.2	6.3	17	84
Proline	115.0633	**$[M+H]^+$**, $[M-H]^-$	3.6	3.6	3.6	0.23	3.5	bg
Propionyl-L-carnitine	217.1314	**$[M+H]^+$**, $[M-H]^-$	2.4	2.4	2.4	0.22	22	8.7
Pseudouridine	244.0695	**$[M-H]^-$**, $[M+H]^+$	3.3	3.3	3.3	12	24	24
Pyruvate	88.01604	**$[M-H]^-$**	1.4	1.5	1.4	3.5	bg	0
Ribose	150.0528	**$[M-H]^-$**	3.2	n.d.	n.d.	150	bg	bg
Ribose-5-phosphate + Ribulose-5-phosphate	230.0192	**$[M-H]^-$**, $[M+H]^+$	3.9-4.1	4.1-4.2	4.9-5	9.2	14	92
S-(Adenosyl)-methionine	398.1372	**$[M+H]^+$**, $[M-H]^-$	4.3	4.3	4.3	119	bg	159
Sarcosine	89.04768	**$[M+H]^+$**, $[M-H]^-$	4.2	4.2	4.2	8.9	89	9
Sedoheptulose-7-phosphate	290.0403	**$[M-H]^-$**, $[M+H]^+$	4.7	5	5.2	2.9	29	29
Seleno-methionine	196.9955	**$[M+H]^+$**, $[M-H]^-$	3.3	3.3	3.2	0.79	39	79
Serine	105.0426	**$[M-H]^-$**, $[M+H]^+$	5.3	5.2	5.3	21	bg	bg
Serotonine	176.0950	**$[M+H]^+$**, $[M-H]^-$	1.4	1.3	1.4	1.2	7.0	7.0

Compound	Neutral exact mass [m/z]	Species	Retention time [min]			LOD [ng/mL]		
			Solvent	Urine	Plasma	Solvent	Urine	Plasma
Spermidine	145.1579		Not detected					
Spermine	202.2157		Not detected					
Succinate	118.0266	**[M-H]**⁻	1.9	1.9	2.1	4.7	35	4.7
Thiamine hydrochloride	265.1123		Not detected					
Thymidine	242.0903	**[M-H]**⁻, [M+H]⁺	1.5-1.9	1.5-1.9	1.5-1.9	2.4	24	7.3
Thymidine 5'-monophosphate	322.0566	**[M-H]**⁻, [M+H]⁺	2	2.1	2.4	13	13	13
Thymine	126.0429	**[M-H]**⁻, [M+H]⁺	1.9	1.9	1.9	0.38	7.6	5.0
Trehalose	342.1162	**[M-H]**⁻, [M+H]⁺	5.1	5.1	5.1	14	68	17
Tryptophan	204.0899	**[M-H]**⁻, [M+H]⁺	3.3	3.3	3.3	2.0	bg	bg
TTP (Thymidinetriphosphate)	481.9893	**[M-H]**⁻, [M+H]⁺	8.8-9	8.7	n.d.	145	193	bg
Tyrosine	181.0739	**[M-H]**⁻, [M+H]⁺	4.1	4.1	4.1	9.1	bg	bg
UMP	324.0359	**[M-H]**⁻, [M+H]⁺	3.3	3.9	4.7	13	130	162
Uracil	112.0273	**[M-H]**⁻, [M+H]⁺	2.2	2.2	2.2	11	bg	5.6
Uridine	244.0695	**[M-H]**⁻, [M+H]⁺	2.4	2.4	2.4	12	bg	122
Uridine 5'-diphosphate	404.0022	**[M-H]**⁻, [M+H]⁺	8.2-8.6	8-8.4	8.2-8.5	12	162	202
Uridine 5'-triphosphate	483.9685	**[M-H]**⁻, [M+H]⁺	9	8.6-8.8	n.d.	484	242	bg
Valine	117.078979	**[M-H]**⁻, [M+H]⁺	3.6	3.6	3.6	5	bg	bg
Xanthine	152.033425	**[M-H]**⁻, [M+H]⁺	2.6	2.7	2.7	76	bg	61
Xylose	150.052824	**[M-H]**⁻	3.9	n.d.	n.d.	1.5	bg	bg

References

1. Pang Z, Chong J, Zhou G et al. MetaboAnalyst 5.0: narrowing the gap between raw spectra and functional insights. Nucleic Acids Res 2021;49:W388-W396.
